# Effectiveness of a digital clinical decision support algorithm for guiding antibiotic prescribing in pediatric outpatient care in Rwanda: A pragmatic cluster non-randomized controlled trial

**DOI:** 10.1371/journal.pmed.1004692

**Published:** 2026-02-26

**Authors:** Alexandra V. Kulinkina, Victor P. Rwandarwacu, Joseph Habakurama, Ludovico Cobuccio, Martin Norris, Emmanuel Kalisa, Cassien Havugimana, Angelique Ingabire, Gillian A. Levine, Rainer Tan, Vincent Faivre, Alan Vonlanthen, Marie-Annick Le Pogam, Kaspar Wyss, Lisine Tuyisenge, Jean Claude S. Ngabonziza, Valérie D’Acremont

**Affiliations:** 1 Swiss Tropical and Public Health Institute, Allschwil, Switzerland; 2 University of Basel, Basel, Switzerland; 3 Swiss Tropical and Public Health Institute, Kigali, Rwanda; 4 Centre for Primary Care and Public Health (Unisanté), University of Lausanne, Lausanne, Switzerland; 5 Rwanda Pediatric Association, Kigali, Rwanda; 6 Rwanda Biomedical Center, Kigali, Rwanda; 7 University of Rwanda, Kigali, Rwanda; Makerere University Medical School, UGANDA

## Abstract

**Background:**

Poor adherence to clinical guidelines and diagnostic uncertainty are key contributors to antibiotic overprescription, accelerating antimicrobial resistance. We developed ePOCT+, a digital clinical decision support algorithm (previously published) that integrated oxygen saturation, hemoglobin measurements, and C-reactive protein testing to assist primary care clinicians in managing acutely ill children aged 14 years or below. The goal was to reduce antibiotic prescriptions without compromising clinical recovery.

**Methods and findings:**

We conducted a pragmatic, open-label cluster non-randomized controlled trial in 32 Rwandan health centers allocated to two groups by geography, balancing patient volume and infrastructure. Sixteen sites used ePOCT+ throughout; the other 16 provided standard care for five months before crossing over. This design supported three comparisons: intervention–control (months 1–5), before–after (control sites pre- versus post-crossover), and longitudinal assessment (intervention sites over 10 months). ePOCT+ was deployed in a digital application; the same application (without the algorithm) served as an electronic case report form in the control arm. Outcomes were analyzed using mixed-effects logistic regression, adjusted for age, sex, district, and presenting complaints. Superiority in antibiotic prescription (primary outcome) was defined as ≥25% reduction versus routine care. Non-inferiority for clinical failure (main secondary outcome) was defined as adjusted risk ratio upper 95% CI <1.3. Several other secondary outcomes were analyzed and reported. Between December 2021 and April 2023, 59,921 consultations were enrolled and 47,822 new consultations were analyzed. ePOCT+ uptake averaged 70% across all intervention periods. In the primary per-protocol analysis, antibiotic prescriptions declined from 70.5% to 24.5% in the intervention–control comparison (absolute difference −46.0, 95% CI [−52.5, −39.5]) and to 27.5% in the before–after comparison (absolute difference −43.0, 95% CI [−49.7, −36.4]). Reductions in the intention-to-treat sensitivity analysis were −36.7 (95% CI [−42.1, −31.3]) in the intervention–control comparison and −26.7 (95% CI [−32.2, −21.4]) in the before–after comparison, respectively. Prescription rates remained low (25%–40%) throughout the longitudinal assessment. Clinical failure rates were non-inferior (per-protocol aRR 1.07, 95% CI [0.97, 1.18] and 1.00, 95% CI [0.92, 1.10] in intervention–control and before–after analyses, respectively). In the intervention–control comparison, referral rates (aRR 2.11, 95% CI [1.20, 3.70]), primary hospitalizations (aRR 2.02, 95% CI [1.21, 3.38]), secondary hospitalizations (aRR 1.93, 95% CI [1.17, 3.16]), and severe outcomes (comprised primarily of non-referred secondary hospitalizations; aRR 1.88, 95% CI [1.11, 3.15]) were higher in the intervention arm; no differences were observed in the before–after comparison, suggesting possible baseline differences in patient severity rather than intervention effects. Re-attendance rates did not differ between arms. Malaria testing among febrile cases improved (aRR 1.27, 95% CI [1.08, 1.41]), while treatment adherence remained high. Study limitations included non-randomized design and self-reported outcomes.

**Conclusions:**

ePOCT+ substantially reduced antibiotic prescribing in Rwandan pediatric primary care without compromising clinical recovery. Integration into Rwanda’s national electronic medical record platform is critical to ensure unified clinical and reporting functions. This could streamline service delivery, improve data quality, and promote evidence-based care at scale.

**Trial registration:**

Clinicaltrials.gov NCT05108831

## Author summary

**Why was this study done?:**

- Antibiotics are overprescribed to children in primary care settings across sub-Saharan Africa, accelerating the global threat of antimicrobial resistance.
- Healthcare providers often prescribe antibiotics as a precaution when they are uncertain about diagnoses due to limited time, overwhelming workloads, and lack of access to simple diagnostic tests.
- Digital tools that guide healthcare providers through clinical assessments and recommend appropriate treatments based on guidelines have shown promise in improving care quality, but their impact on reducing unnecessary antibiotic use has been inconsistent.

**What did the researchers do and find?:**

- We conducted a study in 32 health centers in rural Rwanda involving nearly 60,000 consultations with sick children aged 14 years and below to test whether a digital clinical decision support tool called ePOCT+ could safely reduce antibiotic prescriptions.
- ePOCT+ guided healthcare providers through patient assessments and incorporated simple tests (measuring oxygen levels, hemoglobin, and inflammation markers) to guide in antibiotic prescribing.
- Antibiotic prescription rates dropped dramatically from 71% to 25% in health centers using ePOCT+ compared to those providing standard care, and children recovered at similar rates in both groups, demonstrating that fewer antibiotics did not compromise their outcomes.

**What do these findings mean?:**

- This study demonstrates that a comprehensive digital tool combining clinical guidance with simple diagnostic tests and clinical mentorship can substantially reduce unnecessary antibiotic use in resource-limited primary care settings without harming children’s recovery.
- Scaling this approach across Rwanda and similar countries could significantly contribute to global efforts to combat antimicrobial resistance while maintaining quality care for children.
- Study limitations include lack of randomization of health facilities to the intervention and the nature of antibiotic prescription outcome being self-reported by treating clinicians.

## Abstract 3

In a pragmatic cluster non-randomized trial, Alexandra Kulinkina and colleagues investigate the effectiveness of a clinical decision support algorithm for guiding antibiotic prescribing in pediatric outpatient care in Rwanda.

## Introduction

Children in sub-Saharan Africa (SSA) continue to suffer from significant morbidity and mortality [1], with the majority of their medical needs being addressed at the primary care level [2]. Despite widespread adoption of the Integrated Management of Childhood Illness (IMCI) guidelines, their implementation has fallen short of expectations, largely due to poor adherence [3,4]. On average, only half of the recommended clinical assessments are performed during consultations [5]. Contributing factors include insufficient staffing [6,7], physical or cognitive overload and low motivation among healthcare workers [4], and limited access to essential diagnostic tools [8].

Diagnostic uncertainty, time constraints, and decision fatigue lead clinicians to frequently prescribe antibiotics as a precaution [9]. Over 60% of pediatric outpatient consultations in SSA result in antibiotic use [10], often unnecessarily [11]. In addition to short-term side effects, altered microbiota, and compromised immunity [12], exposure to antibiotics in childhood can have long-term health consequences, such as allergies, obesity, and neurodevelopmental disorders [13]. Inappropriate antibiotic use also contributes to antimicrobial resistance (AMR) [14,15], a pressing global health threat now responsible for more deaths than malaria and HIV [16].

Digital clinical decision support algorithms (CDSAs) have been identified by the World Health Organization (WHO) as a strategy to strengthen adherence to evidence-based guidelines and improve the quality of care [17]. While CDSAs have shown promise in enhancing IMCI adherence [18–21], their impact on antibiotic prescribing remains inconsistent, with some studies showing a significant reduction [22–24], but not others [18,20]. Limitations of existing tools include restricted diagnostic scope [25], limited integration of point-of-care testing (e.g., for fever without source) [26], and poor uptake due to low digital literacy [27], lack of supervision, and inadequate clinical mentorship.

To address these gaps, we developed **ePOCT+** (electronic point-of-care tests plus), an advanced digital CDSA tailored to the Rwandan context [28]. Embedded in the **medAL-reader** tablet-based application developed for this study [29], ePOCT+ supports the diagnosis and management of children across the full pediatric age range (1 day to 14 years) and a broad spectrum of acute conditions seen in outpatient primary care settings. The system integrates oxygen saturation and hemoglobin measurements (to help detect severe illnesses) and C-reactive protein (CRP) rapid testing, a biomarker of inflammation that can help guide antibiotic decision-making when interpreted alongside clinical and epidemiological context [30]. The system also supports clinical mentorship via a real-time dashboard providing feedback on antibiotic prescription rates, among other indicators.

This study, which represents the main component of the DYNAMIC Rwanda project, aimed to assess the effectiveness of ePOCT+ in reducing antibiotic prescribing for acutely ill children in Rwandan primary care settings. The project aligns with national priorities to strengthen pediatric care, promote rational antibiotic use [31,32], and advance the digital transformation of the health sector [33].

## Methods

### Study design, setting, and participants

#### Study design.

**Fig 1:**
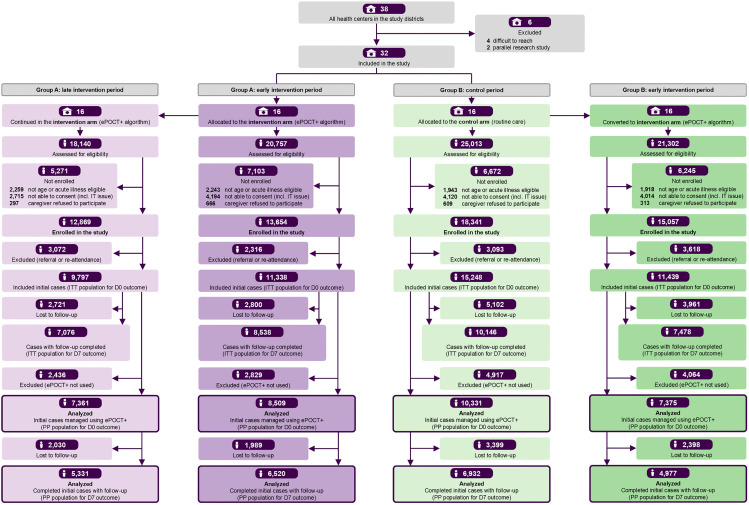
CONSORT flow diagram of participant inclusion by trial phase. Comparison 1: Intervention–control analysis compares Group A: early intervention period (first 5 months) to Group B: control period (first 5 months). Comparison 2: Before–after analysis compares Group B: control period (first 5 months) to Group B: early intervention period (second 5 months). Comparison 3: Longitudinal analysis (exploratory) compares Group A: early intervention period (first 5 months) to Group A: late intervention period (second 5 months). ITT, intention to treat; PP per protocol; D0 day 0 or initial consultation; D7 day 7 after initial consultation; ePOCT+, electronic point of care tests +. Icons were drawn by the authors.

**Fig 2:**
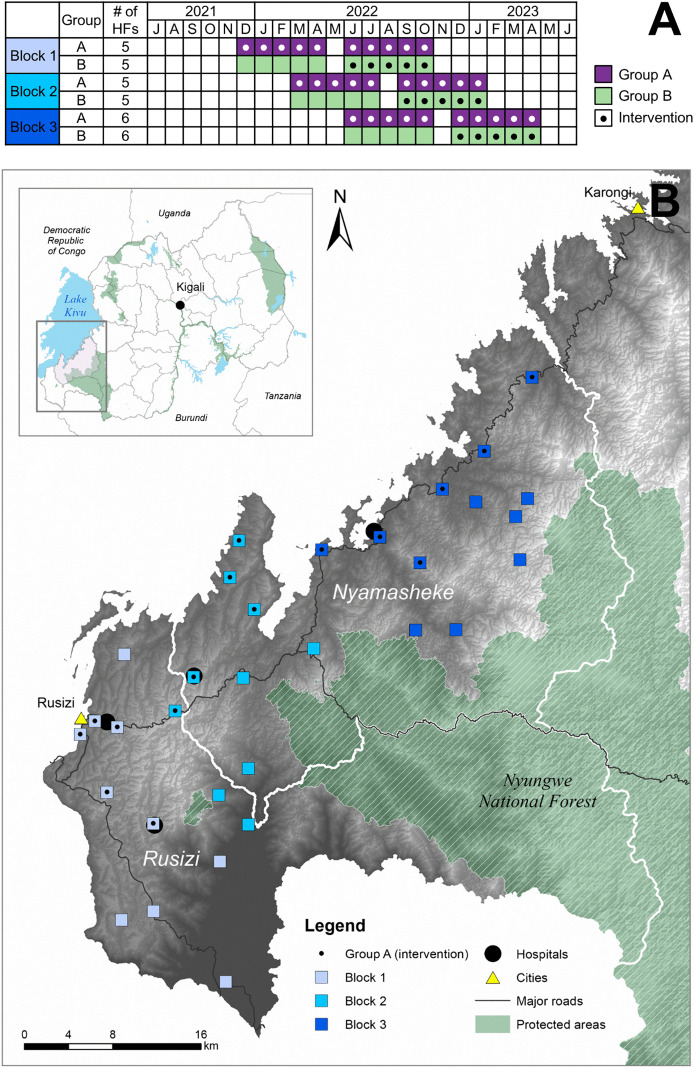
Study design timeline and map of the study area. Panel **A** shows staggered rollout of the intervention across three blocks of geographically allocated health centers between 2021 and 2023 (block colors correspond across panels). Group A (purple in 2A) received the intervention from the beginning (first 5 months representing early and second five months representing late intervention periods), while group B (green) crossed over from control to intervention after 5 months. The three comparisons are: intervention–control (Group A vs. Group B, first 5 months), before–after (Group B pre- vs. post-crossover), and longitudinal (Group A early vs. late intervention). Panel **B** shows a map of the study area created by A.V. Kulinkina in ArcGIS software (version 10.8.2) using the following data sources: country boundaries [Natural Earth]; lakes and waterbodies [RCMRD]; protected areas [WDPA]; district boundaries [Humanitarian Data Exchange]; roads [Humanitarian Data Exchange]; cities [Humanitarian Data Exchange]; elevation [USGS Earth Explorer]; health facilities were mapped by the study team. In the map, group A contains the dot in the health facility symbol for ‘intervention’.

The study was a pragmatic, open-label, two-arm, parallel-group, cluster non-randomized controlled superiority trial, conducted in 32 public health centers in Rusizi and Nyamasheke districts, Western Province, Rwanda. The CONSORT flowchart is presented in Fig 1. The trial employed a hybrid implementation design to support three distinct comparisons: (1) an intervention–control comparison; (2) a before–after comparison; and (3) a 10-month longitudinal assessment (Fig 2A). The intervention–control comparison was the primary analysis; before–after comparison and longitudinal analysis were secondary. Health centers were grouped geographically into three sequentially rolled out implementation blocks of 10–12 centers per block (Fig 2B), with half of the health centers within each block allocated to the intervention (Group A) and half to the control (Group B) arm, enabling the intervention–control comparison. After five months, the intervention was extended to Group B, facilitating the before–after comparison. Group A continued using the intervention, enabling a longitudinal assessment (Fig 2A).

#### Setting.

The study area borders Lake Kivu and the Democratic Republic of Congo to the north-east and Nyungwe National Forest and Burundi to the south-west (Fig 2B). This mountainous region experiences some of the highest precipitation rates in Rwanda [34]. The combined population of the two study districts is approximately 920,000, with 40% being children under the age of 15 years [35]. Nyamasheke district is more rural and remote, with 92% of the population residing in rural areas, as compared to Rusizi (67%) [35], which has substantial commercial and cross-border activity in and around Rusizi city. Rusizi has slightly higher prevalence of electricity coverage (67% versus 62%), while mobile phone access is similar in both districts (around 80%) [35]. Both districts are highly endemic for malaria [36] and experience high prevalence of malnutrition and anemia, while HIV among children is rare, with a prevalence of 0.2% [37]. Up to 90% of the disease burden in Rwanda is treated at the primary care level, comprised of health centers, health posts, and community health workers (CHWs) [36]. The majority of healthcare costs in the public sector are covered by health insurance, most often the government community-based insurance scheme Mutuelle de Santé, with high coverage (>90%) reported by the Rwanda Social Security Board [38].

#### Participants.

Eligible facilities included public health centers, operated either by the government alone or in partnership with faith-based organizations. Health posts (lower-level facilities) were excluded due to limited patient volumes and inconsistent staffing. Children aged 1 day to 14 years (inclusive) presenting with any acute medical or surgical conditions were eligible for inclusion. Children presenting for routine preventive care or chronic disease follow-up, or those for whom informed consent could not be obtained, were excluded. In Rwandan health centers, pediatric consultations are conducted by nurses with A2, A1, or A0 qualifications (representing basic, intermediate, and advanced nursing education levels, respectively) [39]—these were the target users of the CDSA. Consultations for children under five are conducted in dedicated rooms, while older children are typically seen in general consultation areas together with adults.

### Sampling, allocation, and masking

Out of 38 eligible health centers in the two study districts, 32 were selected, after excluding four that were difficult to reach and two that were involved in other research studies. Health centers were grouped geographically into three implementation blocks (Fig 2). Within each block, allocation to intervention (Group A) or control (Group B) was performed to ensure balance across key operational characteristics including average monthly patient volume for children under 5 years, availability of electricity, and use of limited electronic medical records in outpatient consultations. Health facility utilization data were obtained from the national health management information system. Other health facility characteristics were collected by the study team using WHO Service Availability and Readiness Assessment tool, which evaluates availability of basic amenities, equipment, diagnostic capacity, and essential medicines, generating a readiness score ranging from 0%–100% [40]. The allocation process involved reviewing baseline facility data and assigning sites to achieve comparable groups while maintaining geographic separation to limit contamination (Fig 2). A non-randomized study design was chosen so that the intervention could be rolled out gradually to mimic how it would be done in government programs. Research assistants assessing clinical outcome on day 7 were blinded to the intervention allocation. Masking of clinicians, other study staff, and participants was not possible due to the nature of the intervention.

### Procedures

#### Intervention.

**Fig 3:**
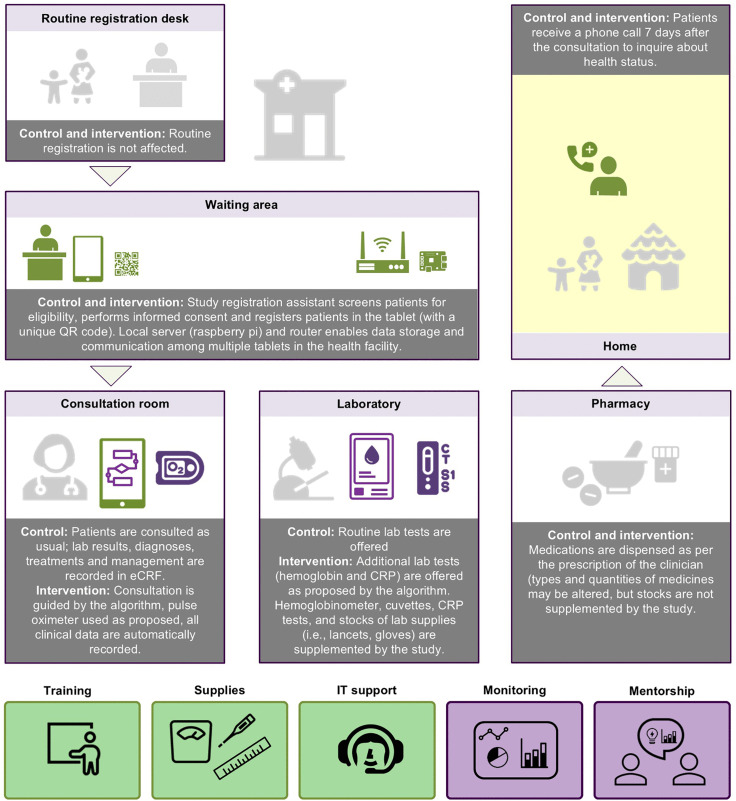
Clinical workflow in the intervention and control arms. The figure illustrates the clinical workflow for intervention and control study arms. In both arms, trained study registration assistants screened patients for eligibility, obtained informed consent, and registered patients using the tablet-based medAL-reader application. In the control arm, consultations followed routine clinical practice, and data were entered into the eCRF. In the intervention arm, consultations were guided by the ePOCT+ algorithm, which integrated clinical assessment with pulse oximetry and additional point-of-care tests (hemoglobin and CRP), where indicated. Laboratory and pharmacy procedures remained similar across groups, though laboratory supplies were supplemented for the intervention. All participants received a follow-up call on day 7 to assess clinical status. Items in light gray are routine and are not affected by the intervention; items in green are provided to both intervention and control arms, items in purple are provided only to the intervention arm. eCRF, electronic case report form; CRP, C-reactive protein. Icons were drawn by the authors or obtained from open sources: https://healthicons.org/ and https://openclipart.org/.

The ePOCT+ algorithm [28], implemented on tablets in the medAL-*reader* application [29], was integrated into the routine outpatient workflow in the participating health centers (Fig 3). In the intervention arm, clinicians used the algorithm to guide history taking, examination, and diagnostic decisions, including point-of-care testing not normally offered to children in the Rwandan primary care setting (hemoglobin, CRP, and pulse oximetry). All lab tests (routine and additional) were performed in accordance with the algorithm recommendations by the laboratory technician, while oxygen saturation was measured by the clinicians in the consultation room. Based on the information entered in medAL-*reader*, the algorithm proposed diagnoses and corresponding treatment options aligned with national pediatric guidelines [28]. Clinicians could accept or override suggestions. A demo of the application is available in S1 Video.

In the control arm, clinicians provided routine care following standard IMCI guidelines and national protocols, with access only to standard lab tests available in primary care (e.g., HIV rapid test, malaria microscopy or rapid test, urine dipstick, stool microscopy). For study purposes, clinicians used the same medAL-*reader* application (without the CDSA) to enter lab test results, diagnoses, treatments, and managements into an electronic case report form (eCRF). The medAL-*reader* application, developed specifically for deploying the ePOCT+ algorithm [29], was fully functional offline (in both arms); however, intermittent internet access was required to send cases to the study database and occasionally update the application or algorithm version.

Both arms received equivalent two-day clinical refresher training; the intervention group received an additional one day of instruction on ePOCT+. Supplies such as thermometers, weighing scales, and MUAC bands, as well as IT support, were provided to all sites, with additional supplementation of lab consumables (e.g., gloves, lancets, alcohol swabs) for point-of-care tests in the intervention arm. A real-time monitoring dashboard provided data for intervention health facilities on key clinical indicators such as antibiotic prescribing, malaria management, and taking of anthropometric measurements. Data was presented in a way that allowed comparison of health facilities to each other (benchmarking). The implementation team used the dashboards to conduct clinical mentorship, which included reviewing the trends together with health facility clinical teams.

#### Eligibility screening and registration.

Trained registration assistants co-hired by the study team and the health centers screened children for eligibility (age 14 years or below and presence of an acute illness). Eligible children whose caregiver provided consent were registered in the medAL-*reader* application, creating a medical case for the clinician in the consultation room to complete. Registration data included demographics and contact information for collection of outcomes. Aggregated screening summaries, including notes on study-specific issues, were submitted daily to the study team, who monitored data quality and provided feedback.

#### Medical consultation (on day 0).

In the intervention arm, all clinical consultation data were captured through the real-time use of the algorithm. In the control arm, lab tests, diagnoses, and treatments were recorded in the eCRF. In addition, on the last page in the medAL-*reader* application, the clinicians in both arms reported whether they prescribed an oral or parenteral antibiotic, and whether they referred the patient for inpatient admission (including overnight observation in the health center or referral to the hospital) or recommended a follow-up consultation. Filling this last page defined a completed case, or adherence to the intervention for per-protocol (PP) analysis.

#### Follow-up phone call (on day 7).

Independent research assistants conducted telephone follow-up with caregivers on day 7 (range 6–14 days) to assess the clinical outcome and treatment-seeking behavior since the initial consultation. Outcome information was recorded in standardized REDCap web forms by five callers who independently selected patients from a shared list. Callers were blinded to the intervention status. In a minority of cases where caregivers were unreachable by phone, CHWs were engaged to visit the household. However, data from these CHW visits were excluded from primary analyses due to systematic differences identified during data quality assessment.

### Outcomes

Clinical adverse events (i.e., unexpected harms potentially attributable to the intervention) were not systematically monitored. Instead, safety was evaluated through pre-specified secondary outcomes as described below.

The primary outcome was the proportion of children prescribed oral or parenteral antibiotics at initial consultation (as reported by clinicians on day 0). Secondary outcomes assessed on day 0 (as reported by clinicians in the application) included: referral or re-attendance visit recommendation; and malaria testing and treatment among febrile cases. Secondary outcomes assessed at day 7 (as reported by caregivers during follow-up calls) included: clinical failure, defined as “not cured” and “not improved,” or occurrence of non-referred secondary hospitalization; hospitalization; severe outcomes, defined as non-referred secondary hospitalization or death; and re-attendance for the same illness; Exploratory outcomes included: taking of additional medications by day 7 that were not prescribed on day 0; and completion of day 0 referral or re-attendance recommendations. Assessment of completion of referral or re-attendance recommendations required matching day 0 provider-reported data with day 7 caregiver-reported data. Complete outcome definitions and specifications of relevant analysis populations are provided in Table 2 and the statistical analysis plan (SAP) (S2 Annex).

**Table 2:** Adjusted outcomes comparing ePOCT+ and routine care across trial phases. Adjusted mixed-effects logistic regression results for primary, secondary, and exploratory outcomes comparing ePOCT+ (intervention) to routine care (control), across the intervention–control and before–after analyses. Results are shown for intention-to-treat (ITT) and per-protocol (PP) populations on day 0 and day 7. Estimates are presented as adjusted relative risks (aRRs) with 95% confidence intervals (CIs), with variables that the model was adjusted for noted. Statistically significant results are bolded. ICC denotes intraclass correlation coefficient. Due to rare events or singularity issues, random effects were excluded from selected models. Severe outcomes comprise non-referred secondary hospitalizations and deaths. Sample sizes vary by outcome and population. Additional footnotes provide detail on specifications and subgroup definitions.

	A: early intervention	A: late intervention	B: control	B: early intervention	Intervention–control^a^			Before–after^a^		
% (*n*/*N*)	% (*n*/*N*)	% (*n*/*N*)	% (*n*/*N*)	ICC	aRR (95% CI)	*p*-value^b^	ICC	aRR (95% CI)	*p*-value^b^
**Outcomes based on all enrolled cases (ITT for day 0)**										
aRR is adjusted for district, monthly enrollment quintile, child age and sex, five complaint categories^c^, and health facility random effect										
Antibiotic prescribed at day 0 [P]	43.3% (4,914/11,338)	46.1% (4,521/9,797)	80.0% (12,205/15,248)	53.3% (6,092/11,439)	0.29	**0.64 (0.49, 0.78)**	**<0.001**	0.09	**0.83 (0.81, 0.84)**	**<0.001**
**Outcomes based on cases completed in the application by the healthcare provider (PP for day 0)**										
aRR is adjusted for district, monthly enrollment quintile, child age, sex, five complaint categories^c^, and health facility random effect										
Antibiotic prescribed at day 0 [P]	24.5% (2085/8,509)	28.3% (2085/7,361)	70.5% (7,288/10,331)	27.5% (2028/7,375)	0.37	**0.28 (0.17, 0.43)**	**<0.001**	0.09	**0.41 (0.39, 0.43)**	**<0.001**
Referral recommended at day 0 [S]	6.55% (557/8,509)	5.15% (379/7,361)	2.79% (288/10,331)	2.55% (188/7,375)	0.20	**2.11 (1.20, 3.70)**	**0.010**	0.13	0.92 (0.76, 1.11)	0.377
Follow-up visit recommended at day 0 [S]	37.5% (3,195/8,509)	21.4% (1,572/7,361)	43.8% (4,521/10,331)	17.1% (1,258/7,375)	0.50	1.25 (0.43, 3.26)	0.669	0.39	**0.23 (0.21, 0.25)**	**<0.001**
**Outcomes based on cases with follow-up done (regardless of application use) (ITT for day 7)**										
aRR is adjusted for district, days to outcome, caller, child age and sex, five complaint categories^c^, and health facility random effect										
Clinical failure by day 7 [S]	13.7% (1,166/8,538)	12.6% (890/7,076)	12.9% (1,305/10,146)	15.1% (1,127/7,478)	0.01	**1.14 (1.02, 1.26)**	**0.017**	0.02	**1.09 (1.02, 1.18)**	**0.016**
Hospitalization by day 7 [S]	1.94% (166/8,538)	2.25% (159/7,076)	1.05% (107/10,146)	1.47% (110/7,478)	0.07	**1.98 (1.40, 2.80)**	**<0.001**	0.01	**1.32 (1.01, 1.73)**	**0.045**
Re-attendace visit by day 7 [S]	5.35% (457/8,538)	5.43% (384/7,076)	4.96% (503/10,146)	5.18% (387/7,478)	0.02	1.15 (0.95, 1.38)	0.159	0.01	0.99 (0.87, 1.13)	0.894
Additional medications taken by day 7^d^ [E]	6.37% (533/8,372)	5.41% (383/7,076)	6.01% (603/10,039)	6.24% (460/7,368)	0.02	1.15 (0.95, 1.39)	0.140	0.01	0.98 (0.87, 1.10)	0.748
**Outcomes based on cases completed in the application and with follow-up done (PP for day 7)**										
aRR is adjusted for district, days to outcome, caller, child age and sex, five complaint categories^c^, and health facility random effect (except secondary hospitalization, severe outcome, and completed re-attendance visit)										
Clinical failure by day 7 [S]	14.0% (914/6,520)	12.4% (661/5,331)	12.8% (889/6,932)	14.5% (724/4,977)	0.01	1.07 (0.97, 1.18)	0.154	0.02	1.00 (0.92, 1.10)	0.917
Primary hospitalization (day 0 or day 1) [S]	1.30% (85/6,520)	1.56% (83/5,331)	0.58% (40/6,932)	0.94% (47/4,977)	0.08	**2.02 (1.21, 3.38)**	**0.007**	0.01	1.29 (0.83, 1.99)	0.261
Secondary hospitalization (day 2+) [S]	0.74% (48/6,520)	0.71% (38/5,331)	0.39% (27/6,932)	0.64% (32/4,977)	0.03	**1.93 (1.17, 3.16)**	**0.010**	--	1.66 (0.97, 2.82)^e^	0.062
Severe outcome by day 7^f^ [S]	0.66% (43/6,520)	0.66% (35/5,331)	0.36% (25/6,932)	0.58% (29/4,977)	--	**1.88 (1.11, 3.15)** ^e^	**0.018**	--	1.64 (0.94, 2.85)^e^	0.081
Non-scheduled re-attendance visit by day 7 [S]	3.85% (251/6,520)	4.37% (233/5,331)	2.70% (187/6,932)	4.22% (210/4,977)	0.14	1.12 (0.70, 1.77)	0.633	0.04	**1.32 (1.08, 1.60)**	**0.006**
Completed referral^g^ [E]	9.76% (45/461)	13.7% (41/300)	9.00% (18/200)	14.0% (20/143)	0.09	1.19 (0.54, 2.44)	0.653	0.07	0.80 (0.35, 1.79)	0.596
Completed re-attendance visit^g^ [E]	4.95% (120/2,425)	5.36% (62/1,156)	4.31% (131/3,036)	6.50% (61/939)	0.02	1.04 (0.79, 1.36)	0.762	--	1.28 (0.95, 1.72)^e^	0.108
**Malaria outcomes based on febrile cases completed in the application**										
aRR is adjusted for district, child age and sex, and health facility random effect (except untested treated with antimalarials and positive treated with antibiotics)										
Tested for malaria^h^ [S]	81.3% (2,932/3,606)	83.2% (2,490/2,994)	65.4% (2,912/4,453)	76.2% (2,248/2,948)	0.21	**1.27 (1.08, 1.41)**	**0.008**	0.15	**1.19 (1.15, 1.23)**	**<0.001**
Positive treated with antimalarials [S]	95.8% (91/95)	94.2% (65/69)	88.1% (74/84)	88.4% (38/43)	0.80	1.22 (0.91, 1.23)	0.084	0.65	1.01 (0.32, 1.54)	0.974
Negative treated with antimalarials^i^ [S]	0.00% (0/2,837)	0.00% (0/2,421)	0.04% (1/2,828)	0.18% (4/2,205)	--	--	--	--	--	--
Untested treated with antimalarials [S]	0.74% (5/674)	0.20% (1/504)	0.32% (3/1,541)	0.43% (3/700)	0.39	3.31 (0.57, 18.7)	0.180	--	2.46 (0.49, 12.0)^e^	0.273
Positive treated with antibiotics [E]	16.8% (16/95)	27.5% (19/69)	13.1% (11/84)	6.98% (3/43)	0.17	0.84 (0.21, 2.66)	0.790	--	0.45 (0.11, 1.19)^e^	0.134

[P] Primary outcome; [S] Secondary outcome; [E] Exploratory outcome.

^a^Intervention–control analysis compares health facilities in Group A: early intervention to Group B: control; Before–after analysis compares health facilities in Group B: control to Group B: early intervention.

^b^*P*-values from Wald *z*-tests.

^c^Complaint categories: fever, respiratory, gastrointestinal, ear nose throat mouth, skin.

^d^Question about additional medicines was not asked of children who were hospitalized, hence the smaller sample size.

^e^Due to singularity issues, health facility random effect was excluded from some of the models (estimates and confidence intervals were nearly identitical).

^f^Severe outcomes included non-referred secondary hospitalizations and deaths (3 in A: early intervention, 3 in A: late intervention, 1 in B: control, 1 in B: early intervention).

^g^Sample size reduced because only cases that were referred or for which re-attendance visit was recommended are considered.

^h^Malaria positivity rates were similar in the four groups: A: early intervention (3.2%); A: late intervention (2.8%); B: control (2.9%); B: early intervention (1.9%).

^i^Outcome was too rare to model.

### Statistical analysis

#### Sample size.

The sample size was calculated to detect a ≥25% relative reduction in antibiotic prescribing in the intervention–control comparison with 80% power and *α* = 0.05, assuming an ICC of 0.025, cluster size of 660 patients over an initial planned period of three months, and a conservative baseline antibiotic prescription rate estimate of 35%. A minimum of 12 centers per arm was required; all 32 eligible centers were enrolled to enhance geographic coverage and improve estimates of secondary outcomes. Due to lower-than-expected enrollment rates, the intervention–control comparison period was extended from three to five months (December 2021 to April 2022 for Block 1, March to July 2022 for Block 2, June to October 2022 for Block 3; see Fig 2A). The final PP sample for the intervention–control comparison averaged 589 patients per cluster.

#### Analysis populations.

For day 0 outcomes, intention-to-treat (ITT) analyses included all new consultations (excluding referrals and re-attendance visits) regardless of completion status in the medAL-*reader* application. PP analyses included only completed consultations, i.e., those for which the clinician filled out the final page of the application where antibiotic prescription and referral decisions were reported. For day 7 outcomes, the ITT population comprised all new consultations with available follow-up data, while the PP population comprised completed cases with follow-up data. For analysis of the primary outcome (antibiotic prescription), there is a discrepancy between the protocol and the SAP. The SAP correctly states that the primary analysis is PP. For the ITT population, antibiotic prescription was missing for cases that were not completed in the application; thus, ITT analysis conservatively assumed that an antibiotic was prescribed in all incomplete cases in both study arms.

#### Hypothesis testing.

Superiority in antibiotic prescription was defined as a 25% or more relative reduction between routine care and ePOCT+. Noninferiority for clinical failure was defined as the upper limit of the 95% confidence interval (CI) of the adjusted risk ratio (aRR) <1.3. Prespecified subgroup analyses stratified the results by sex, age group, and presenting complaint and explored effect differences across these variables.

#### Statistical modeling.

Mixed-effects logistic regression models were used for all outcomes, with health center as a random effect. In models where singularity was observed, the random effect was removed without affecting estimates. Patient random effects were not accounted for due to relatively few patients having multiple consultations and resulting model convergence issues. Separate mixed-effects logistic regression models were fit for each comparison (intervention–control, before–after) using the relevant data subsets, as each comparison addressed a distinct research question. All data fields were mandatory in the medAL-*reader* application—thus, there were no missing data except in cases where the application was not used or if the patient was lost to follow-up. Missing observations were excluded from analysis in the definitions of the PP and ITT populations.

All models were adjusted for age, sex, district, and five presenting complaint categories (fever, respiratory, gastrointestinal, ear nose throat and mouth, skin). Models for day 0 outcomes were additionally adjusted for monthly patient enrollment volume (categorized in quintiles) as a proxy for facility workload; day 7 models were additionally adjusted for time to follow-up (continuous, days from initial consultation to follow-up call) and caller identity (categorical). These additional covariates were not specified in the study protocol but were identified as important in exploratory analyses.

Relative risks and absolute differences were estimated using marginal probabilities [41]; confidence intervals were derived via the Delta method [42]. Although the SAP specified reporting odds ratios, we report relative risks instead because prevalences of antibiotic prescription and clinical failure outcomes exceeded 10% at baseline, and odds ratios substantially overestimate effect sizes for common outcomes [43]. Relative risks also provide more accurate and interpretable estimates of intervention effects for clinicians and policymakers.

#### Sensitivity analyses.

To assess robustness of findings to potential temporal confounding, we conducted sensitivity analyses for the primary outcome, additionally adjusting for calendar month (continuous) and implementation block (categorical) across all comparisons and analysis populations. Sensitivity analyses were not specified in the protocol and were done post-hoc.

#### Exploratory analyses.

Primary analyses were complemented by exploratory analyses of the impact of increased frequency of anthropometric measurements and availability of rapid diagnostic tests on diagnosis and treatment decisions, and acceptance patterns of algorithm-proposed diagnoses and treatments by healthcare providers.

#### Software and data availability.

All analyses were conducted in R software (version 4.2.2) [44] using the CRTSize package (version 1.2) [45] for sample size estimation and custom scripts for statistical modeling. De-identified data used in the analysis can be found at https://doi.org/10.5281/zenodo.15870974.

### Ethics and inclusion

The study was approved by the Rwanda National Ethics Committee (original protocol 752/RNEC/2020; amendment 975/RNEC/2021) and the Swiss Vaud Cantonal Ethics Commission (CER-VD 2020-02799). The amendment included updates to secondary outcome definitions, health facility selection, and sample size/number of beneficiaries calculations. These changes were approved and implemented before the start of the trial. The trial was registered on ClinicalTrials.gov (NCT05108831; https://clinicaltrials.gov/study/NCT05108831 posted on 05.11.2021). The amended protocol (S1 Annex) and SAP (S2 Annex) are linked to the trial registry. After the conclusion of the trial, the protocol was amended again (246/RNEC/2023) for routine implementation of the intervention in the health system by shortening the consent process and removing day 7 follow-up.

The study was also reviewed and approved by the National Health Research Committee (NHRC/2020/Prot/031) and the National Institute of Statistics of Rwanda (0654/2020/10/NISR). Subsequently, the Ministry of Local Government authorized the study team to work in Rusizi and Nyamasheke districts. Upon introduction of the project to the mayors’ offices and district hospitals, letters of collaboration were signed with each participating health center. Finally, the study was introduced to the Sector Council, and community engagement events were held in the catchment villages of each health center, with over 7,000 participants. Written informed consent was provided by parents/legal guardians); children aged 12 years and above also provided verbal assent. This study is reported as per CONSORT guideline (S1 CONSORT Checklist).

From the conceptualization stage, the study team worked with the Ministry of Health (MoH; via a Memorandum of Understanding) and the Rwanda Biomedical Center (RBC; via a collaboration agreement). A steering committee, with representatives from MoH, RBC, WHO, UNICEF, Rwanda Food and Drug Administration, Rwanda Pediatric Association, Rusizi and Nyamasheke districts, oversaw the study. Study approaches were regularly discussed with the national Maternal and Child Health Technical Working Group. At district level, the study team participated in quarterly meetings of the Joint Action Development Forum where activities of partner institutions are coordinated. Upon conclusion of the study, the findings were presented to the participating health centers, district hospitals, District Health Management Teams, relevant national programs at RBC and MoH, and finally to a broader audience of stakeholders in a dissemination event held in Kigali in March 2024.

## Results

### Baseline characteristics of health facilities and patients

A total of 32 health centers were included in the trial (Fig 1): 16 in the intervention group (Group A, ePOCT+) and 16 in the control group (Group B, routine care). The phased rollout (Fig 2A) embedded ePOCT+ into the routine consultation workflow (Fig 3). Health centers were balanced in terms of district (Rusizi versus Nyamasheke), ownership (public versus faith-based partnership), and service readiness scores. Power supply and use of computers in the consultation rooms were also similar. Group A had slightly more outpatient staff (mean 9.3 versus 7.8) and lower median monthly consultation volumes for children under five (236 versus 286), resulting in marginally lower patient recruitment (Table 1).

**Table 1:** Characteristics of participating health facilities and enrolled pediatric patients. The table shows that health facilities were well balanced between groups A and B in terms of district, ownership, service availability scores, power supply, and computer use during consultations. Group A had slightly higher staffing and lower patient volume. Patients were comparable across the four study periods in terms of sex and age distribution, history of hospitalization, and presenting complaints. There were slightly more new consultations in the first half of the study and phone ownership was slightly lower in group B.

	A: early intervention^a^	A: late intervention	B: control^a^^,^^b^	B: early intervention^b^
	(ePOCT+)	(ePOCT+)	(routine care)	(ePOCT+)
**Health centers**	***n* = 16**	***n* = 16**	***n* = 16**	***n* = 16**
District				
Rusizi	6	6	7	7
Nyamasheke	10	10	9	9
Ownership				
Public	7	7	7	7
Faith-based partnership	9	9	9	9
Service Availability and Readiness Assessment (SARA) score^c^				
General services score, % (mean, SD)	71.5 (7.0)	71.5 (7.0)	74.3 (6.7)	74.3 (6.7)
Pediatric services score, % (mean, SD)	74.7 (8.2)	74.7 (8.2)	76.1 (8.1)	76.1 (8.1)
Power supply				
Uninterrupted	9	9	7	7
Outages <2 hours per day	6	6	9	9
Outages ≥2 hours per day	1	1	0	0
Using computer in consultation room for clinical data entry^d^	4	4	4	4
Staffing: # of OPD clinicians (mean, SD)^d^	9.3 (2.4)	9.3 (2.4)	7.8 (2.3)	7.8 (2.3)
Patient load: # of <5 patients per facility per month (median, IQR)^e^	236 (159, 349)	236 (159, 349)	286 (225, 347)	286 (225, 347)
Enrollment: # of <15 patients per facility per month (median, IQR)	151 (124, 222)	155 (117, 194)	207 (130, 294)	173 (125, 231)
Number of health facility days when the study was operating^f^	1,639	1,639	1,639	1,639
Patients enrolled	1,515	1,508	1,541	1,541
Patients not enrolled: registration assistant off duty	43	54	46	37
Patients not enrolled: no trained health worker to use the app	67	62	37	38
Patients not enrolled: power or IT issue	11	15	10	17
Patinets not enrolled: another issue	3	0	5	6
**Patients (enrolled; intention-to-treat)**	***n* = 13,654**	***n* = 12,869**	***n* = 18,341**	***n* = 15,057**
Sex (*n*, %)				
Female	7,067 (51.8%)	5,998 (46.6%)	9,344 (50.9%)	8,061 (53.5%)
Male	6,587 (48.2%)	6,871 (53.4%)	8,997 (49.1%)	6,996 (46.5%)
Age group (*n*, %)				
<2 months	611 (4.5%)	535 (4.2%)	946 (5.2%)	699 (4.6%)
2–59 months	8,852 (64.8%)	8,376 (65.1%)	11,678 (63.7%)	10,324 (68.6%)
5–14 years	4,191 (30.7%)	3,958 (30.7%)	5,717 (31.2%)	4,034 (26.8%)
Consultation type (*n*, %)				
New	11,338 (83.0%)	9,797 (76.1%)	15,248 (83.1%)	11,439 (76.0%)
Follow-up	2,292 (16.8%)	3,066 (23.8%)	2,909 (15.8%)	3,552 (23.6%)
Referral from another health facility or community health worker (CHW)	24 (0.2%)	6 (0.1%)	184 (1.1%)	66 (0.4%)
Hospitalized in the last 14 days (*n*, %)				
Yes	99 (0.7%)	35 (0.3%)	145 (0.8%)	48 (0.3%)
No	13,555 (99.3%)	12,834 (99.7%)	18,196 (99.2%)	15,009 (99.7%)
Phone ownership (*n*, %)				
Primary caregiver or person inside household	12,053 (88.3%)	11,025 (85.7%)	14,936 (81.4%)	12,057 (80.1%)
None or person outside household (including CHW)	1,601 (11.7%)	1,844 (14.3%)	3,405 (18.6%)	3,000 (19.9%)
**Patients (case completed electronically; per-protocol)**	***n* = 8,509**	***n* = 7,361**	***n* = 10,331**	***n* = 7,375**
Complaint categories (*n*, %)				
Respiratory	4,639 (54.5%)	3,642 (49.5%)	5,946 (57.6%)	4,080 (55.3%)
Fever	3,606 (42.4%)	2,994 (40.7%)	4,453 (43.1%)	2,949 (40.0%)
Gastrointestinal	2,530 (29.7%)	2,215 (30.1%)	2,499 (24.2%)	2,308 (31.3%)
Ear nose throat mouth	2,360 (27.7%)	1,891 (25.7%)	935 (9.1%)	1,164 (15.8%)
Skin	975 (11.5%)	874 (11.9%)	883 (8.5%)	706 (9.6%)
Eye	881 (10.4%)	826 (11.2%)	846 (8.2%)	718 (9.7%)
Neurological	973 (11.4%)	646 (8.8%)	745 (7.2%)	636 (8.6%)
Others	898 (10.6%)	714 (9.7%)	1,039 (10.1%)	492 (6.7%)
Injury/musculoskeletal	240 (2.8%)	221 (3.0%)	261 (2.5%)	194 (2.6%)

^a^Intervention–control analysis compares Group A Early intervention period (first 5 months) to Group B Control period (first 5 months).

^b^Before–after analysis compares Group B Control period (first 5 months) to Group B Early intervention period (second five months).

^c^Score (%) was calculated as the number of available commodities/services divided by the total number of commodities/services assessed per SARA methodology.

^d^Source: Service Availability and Readiness Assessment (SARA) conducted in December 2020.

^e^Source: Health Management Information System (HMIS) data for 2019–2020.

^f^Sum of health facilities multiplied by number of days (only Monday-Friday are considered because registration assistants did not work on weekends).

The study operated over 6,556 health facility days between 1 December 2021 and 30 April 2023, not counting weekends or public holidays. Recruitment occurred on 6,105 (93%) days, with missed days primarily due to absences of registration assistants (3%) or clinicians trained on the use of the application (3%), and rarely due to IT, power, and other issues (1%) (Table 1). Of 85,212 patient consultations screened, 59,921 (70%) were enrolled. Non-enrollment was attributed to ineligibility (10%); logistical barriers to consenting or enrolling patients (18%)—such as IT issues, lack of trained clinicians, adolescents coming unaccompanied to the health center, lack of witnesses—and rarely caregiver refusal (2%) (Fig 1).

The enrolled cohort (*n* = 59,921) was balanced between groups (A and B) in terms of sex (52% versus 51% female) and age (65% versus 64% aged 2–59 months) distribution. Most consultations were for new illnesses (80%), and fewer than 1% of patients had been recently hospitalized. Household access to a phone was higher in Group A (88% versus 81%); those without a phone in the household reported access via neighbors, friends, or CHWs (Table 1).

Excluding 12,099 referral or re-attendance visits, 47,822 new consultations were eligible for the ITT analysis. Among these, 70% of cases were completed in medAL-*reader* by the healthcare providers (i.e., antibiotic outcome data were entered), yielding 33,576 consultations for the PP analysis of day 0 outcomes. Case completion rates were lower in Group B (68% in control and 64% in early intervention period) than in Group A (75% in early and late intervention periods) (Figs 1 and S1). Case completion corresponds to ePOCT+ uptake during the intervention periods. Follow-up data were available for 33,238 consultations (ITT) and 23,760 consultations (PP) for day 7 outcomes. Loss to follow-up was 30% overall, and higher in Group B (34%) than in Group A (26%), consistent with phone access profile. Approximately 66% of the lost to follow-up cases had been attempted by CHWs; however, this data was ultimately omitted from analysis due to systematically much lower clinical failure frequency than among phone calls, suspecting significant data quality issues (S2 Fig).

The most common presenting complaints were respiratory symptoms, fever and gastrointestinal issues (Table 1). The distribution of complaints was similar across study groups, although skin and ear nose mouth and throat problems were more frequently reported in Group A.

### Primary outcome: Antibiotic prescription

**Fig 4:**
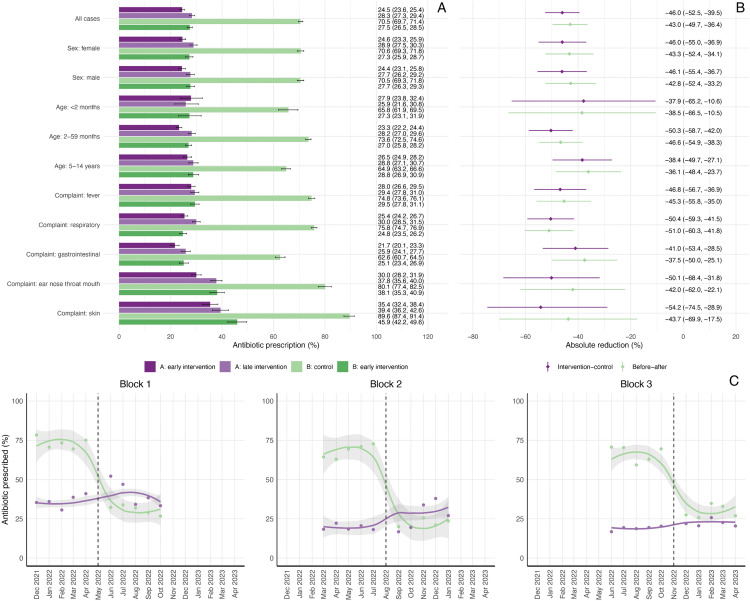
Antibiotic prescription proportions and differences across trial periods and subgroups in the per-protocol population. Panel A shows the proportion of children prescribed antibiotics on day 0 across four study periods—control (Group B), early and late intervention (Group A), and early intervention post-crossover (Group B)—overall and stratified by sex, age group, and presenting complaint. Panel B presents absolute reductions in antibiotic prescription between groups, comparing the early intervention phase of Group A to the control phase of Group B (intervention–control comparison), and the pre- and post-intervention phases of Group B (before–after comparison). Use of ePOCT+ was associated with substantial reductions in antibiotic prescriptions in all subgroups, with the largest reductions observed for skin, respiratory, and ear nose throat and mouth complaints. Panel C illustrates the monthly trends in antibiotic prescribing over time in each implementation block, highlighting drastic reduction in Group B (green) post-crossover (vertical dashed line) and consistency in sites using ePOCT+ (purple).

Under routine care (Group B), antibiotics were prescribed in 70.5% of consultations for acute illness (Table 2 and Fig 4A), with highest proportions in children aged 2–59 months (73.6%) and for skin complaints (89.6%) (Fig 4A). In the primary PP analysis, use of ePOCT+ reduced antibiotic prescribing to 24.5% (Group A) and 27.5% (Group B), corresponding to absolute reductions of 46.0% (95% CI [−52.2, −39.5]) in the intervention–control comparison and 43.0% (95% CI [−49.7, −36.4]) in the before–after comparison. The largest reductions were observed in the 2–59 age group and for respiratory, ear nose throat and mouth, and skin complaints (Fig 4B).

In the ITT sensitivity analysis, antibiotic prescription decreased from 80.0% to 43.3% (intervention–control) and to 53.3% (before–after), corresponding to the absolute reductions of 36.7% (95% CI [−42.1, −31.3]) and 26.7% (95% CI [−32.2, −21.4]), respectively (Table 2 and S3 Fig). In the longitudinal analysis of Group A health facilities, antibiotic prescriptions ranged from 20% to 40%, remaining similar throughout the 10 months of the intervention period in blocks 1 and 3, and increasing slightly in the second half of the intervention period in block 2 (Fig 4C). Facility-level variation was marked, with some ePOCT+ sites maintaining high prescription proportions and some control sites demonstrating low baseline antibiotic use (S4 Fig).

Sensitivity analyses showed that effect estimates were robust to temporal adjustment (S1 Table). In the intervention–control comparison, adjusted relative risks changed minimally from the main analysis (PP: 0.28 to 0.31; ITT: 0.64 to 0.68). The before–after PP comparison showed moderate attenuation (0.41 to 0.50), consistent with greater susceptibility to temporal confounding in this design, though the ITT analysis remained stable (0.83 to 0.85). Calendar month was significant only in the intervention–control PP model. Implementation block was not significantly associated with antibiotic prescribing in any analysis.

### Secondary and exploratory outcomes: clinical failure, referral, hospitalization, malaria management, additional medications

In the adjusted PP multivariable analysis, clinical failure did not differ significantly among children managed according to routine care (12.8%) versus with ePOCT+ (aRR 1.07, 95% CI [0.97, 1.18], *p* = 0.154 (intervention–control); aRR 1.00, 95% CI [0.92,1.10], *p* = 0.917 (before–after). In the ITT analysis, effect estimates were modestly higher in both comparisons, but within the non-inferiority margins (aRR 1.14, 95% CI [1.02, 1.26], *p* = 0.017 and 1.09, 95% CI [1.02, 1.18], *p* = 0.016, respectively) (Table 2). No major differences were observed across subgroups, though failure rates in the ePOCT+ group were slightly higher for skin conditions, which typically take longer to resolve (S5 Fig).

In the intervention–control comparison, referral rates were two times higher in the intervention arm compared to the control arm (6.6% versus 2.8%; aRR 2.11, 95% CI [1.20, 3.70], *p* = 0.010) (Table 2). Primary and secondary hospitalizations (0.6% and 0.4%, respectively, at baseline) were also approximately two times higher in the intervention arm (aRR 2.02, 95% CI [1.21, 3.38], *p* = 0.007 and aRR 1.93, 95% CI [1.17, 3.16], *p* = 0.010, respectively). However, the adherence to referral advice was low in both study arms; less than 15% of children for whom referral was recommended on day 0 were hospitalized by day 7. Severe outcomes, comprised primarily of non-referred secondary hospitalizations (with very few deaths), were also more frequent in the intervention group (0.66% versus 0.36%; aRR 1.88, 95% CI [1.11, 3.15], *p* = 0.018). No differences in referrals, hospitalizations or severe outcomes were observed in the PP analyses in the before–after comparison (Table 2). Most of the children with non-referred secondary hospitalization had been cured or improved by day 7, and cure rates among these children were similar regardless of antibiotic treatment on day 0 (S2 Table).

According to the caregiver report on day 7, a very small percentage of children (<6.5%) subsequently took medications in addition to those prescribed to them at the study consultation, with no difference between the ePOCT+ and routine care arms (Table 2). Malaria testing of febrile cases improved with the use of ePOCT+ from a baseline of 65.4% in the control group (aRR 1.27, 95% CI [1.08, 1.41], *p* = 0.008). Treatment adherence was already high (>88%) and did not change significantly. Results of unadjusted analyses are provided in S3 Table.

### Additional exploratory analyses: impact of ePOCT+ on diagnosis and treatment decisions

**Fig 5:**
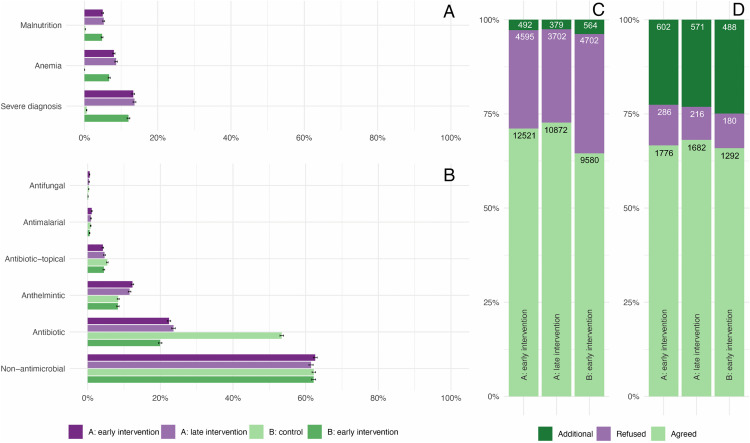
Impact of ePOCT+ on diagnosis and treatment, and adherence to algorithm recommendations. Panel **A** shows the proportion of consultations in which severe diagnoses, anemia, and malnutrition were identified. Severe diagnoses include any conditions requiring referral (e.g., severe pneumonia, severe dehydration, severe clinical infection). Use of ePOCT+ led to marked increases in the detection of all three conditions compared to routine care. Panel **B** presents the distribution of medication prescriptions by category, including antibiotics, antimalarials, antifungals, and non-antimicrobial agents. Aside from a reduction in antibiotic use, the overall prescription pattern remained stable across groups. Panel **C** displays clinician agreement with individual instances of algorithm-suggested diagnoses (and manually added diagnoses). While most were accepted, approximately 25% were rejected across intervention periods. Panel **D** shows clinician agreement with individual instances of algorithm-suggested antibiotics (and manually added antibiotics. Rejection of proposed antibiotics was rare, but approximately 25% of prescribed antibiotics were added, indicating partial algorithm adherence.

Routine use of anthropometric and hemoglobin measurements with ePOCT+ improved detection of malnutrition (from <0.5% to ~5%) and anemia (from 0% to ~8%). Severe diagnoses (requiring referral based on IMCI classification and national guidelines) were also identified more frequently (<1% versus 12%–13%) (Fig 5A). Apart from the reduction in oral and parenteral antibiotics, there were no significant changes in medicine prescriptions (Fig 5B). Some gaps were noted in compliance with ePOCT+ recommendations. Clinicians rejected diagnoses proposed by the algorithm approximately 25% of the time, while manually added diagnoses were rare (Fig 5C). On the other hand, rejection of proposed antibiotics was rare, but manual addition when not proposed by ePOCT+ constituted approximately 25% of all prescribed antibiotics (Fig 5D).

## Discussion

This pragmatic trial demonstrated a substantial reduction in antibiotic prescription rates for children aged 14 years or below managed in primary care settings using a CDSA integrated with point-of-care tests and supported by clinical mentorship. In the PP analysis, antibiotic prescriptions declined nearly 3-fold; in the ITT analysis, the reduction was 2-fold. The most pronounced reductions were observed in children aged 2–59 months—representing over 65% of the outpatient pediatric caseload—and among those presenting with respiratory, skin, or ear nose throat and mouth complaints, which together accounted for the majority of consultations. These findings are consistent with the cluster-randomized trial of ePOCT+ in Tanzania, where antibiotic prescribing also declined approximately 3-fold from 70% at baseline in the PP analysis and 2-fold in the ITT analysis [24]. However, in a recent pre-post study in Kenya and Senegal of a similar algorithm limited to children under the age of 5 years, a more modest reduction in antibiotic prescription (15%–23%) was observed. Notably, the algorithm used in that study included pulse oximetry but excluded hemoglobin and CRP diagnostic tests, and the implementation and clinical mentorship strategies differed [46]. These factors possibly contributed to differences in the impact of the CDSA on antibiotic prescribing.

The immediate effect of the intervention appeared to be sustained across a 10-month longitudinal assessment. In our study, antibiotic reductions were not accompanied by increased prescribing of other medications, suggesting that clinicians did not feel the need to compensate by substituting other medications. Similarly, parents did not give additional medicines to their children at home, except in a minority of cases, balanced across study arms.

Algorithm adherence, however, remained suboptimal. Approximately 25% of the prescribed antibiotics were added manually by clinicians and not recommended by the algorithm. This high rate suggests that this was not related to special clinical cases outside guidelines but rather persistent reliance on clinical judgement and the need for ongoing mentorship. Antibiotic prescribing rates—and the rate of manual additions when not proposed by ePOCT+—varied widely across facilities and individual clinicians. These findings suggest that with stronger supervision and better adherence, prescribing rates could fall even further, approaching the 11% observed in a prior efficacy study in controlled research conditions of an earlier ePOCT algorithm version [22]. Further analyses include exploring the relative contributions of the algorithm, CRP test, and monitoring and clinical mentorship enabled by the real-time dashboards on antibiotic prescribing.

Importantly, ePOCT+ improved the detection of malnutrition and anemia through routine assessment of anthropometrics and hemoglobin. Malaria testing rates also increased significantly, although malaria treatment adherence was already high and remained unchanged. These improvements reflect how algorithm-guided care may contribute to overall diagnostic quality.

Despite the impact demonstrated in the PP population, it is important to note that 25%–30% of the enrolled cases were not managed with ePOCT+. Lack of integration among reporting tools, with paper-based (IMCI registers, health insurance forms) and, in some health centers, electronic medical records, being used in parallel, made workflows time-consuming. Staffing constraints varied widely, with the most pronounced challenges in remote Nyamasheke district (group B), where high staff turnover and patient volume hampered consistent use of the application. These systemic factors underscore the importance of harmonizing clinical, administrative, and reporting tools into a unified digital system, particularly in low-resource settings. Individual-level factors, including digital literacy and motivation of clinicians to use CDSAs, warrant further investigation.

Although the findings support clinical safety of ePOCT+, the study was not powered to evaluate these secondary outcomes. Clinical cure rates were similar across groups in both intervention–control and before–after comparisons. Non-inferiority in clinical outcomes of the ePOCT+ algorithm was also confirmed in the cluster-randomized trial in Tanzania [24]. The higher referral and hospitalization rates in Group A compared to Group B in the intervention–control comparison, but not in the before–after comparison, suggest possible baseline differences in patient severity between cluster groups, despite balanced observable facility characteristics. Higher rates of severe diagnoses in Group A also support this hypothesis.

Despite the inclusion of oxygen saturation and hemoglobin measurements in ePOCT+ to improve detection of severe illnesses, referrals did not increase in the Tanzanian trial [24], nor in the Kenyan and Senegalese study of a similar algorithm for under five children that also included pulse oximetry [46]. Complementary concerning findings were low referral completion among caregivers of children and frequent non-referred hospitalizations, indicating remaining challenges both on behalf of clinicians in identifying severe cases and on behalf of patients reaching the hospital when referred. These patterns warrant further study.

Our study has several strengths and limitations. Strengths include a large sample size, multiple comparisons, and broad patient inclusion criteria, making the findings generalizable to the general pediatric outpatient population. Limitations include a clinician self-reported measure of antibiotic prescription, an approach often used in pragmatic studies [47], and relatively high loss-to-follow up (~30%); although not dissimilar to that of other SSA studies [48]. The clinician-reported nature of the antibiotic prescription outcome may mean that the reduction we found in the PP analysis is over-estimated, and most likely the real reduction is closer to that found in the ITT analysis, as was the case in the ePOCT+ study in Tanzania [21].

The non-randomized design is an important limitation of this study; randomization would have been preferable for eliminating selection bias and strengthening causal inference. We chose a non-randomized, staggered implementation design to align with how the Rwandan Ministry of Health would likely scale the intervention in routine practice, prioritizing external validity and policy relevance [49]. This pragmatic approach is consistent with the PRECIS-2 framework [50] and increasingly used methods for evaluating complex interventions under routine operational conditions [51,52], though residual confounding could still be present despite efforts to balance groups on observable characteristics.

To address these limitations, we employed multiple complementary comparisons (intervention–control, before–after, and longitudinal) and conducted sensitivity analyses adjusting for temporal trends. The substantial reduction in antibiotic prescribing was consistent across all analyses, providing reassurance about the robustness of findings. The present study’s design enhances understanding of real-world implementation outcomes and health system integration challenges, while acknowledging the trade-offs in internal validity inherent to pragmatic, non-randomized evaluation.

In this pragmatic trial, the implementation of ePOCT+ substantially reduced antibiotic prescribing in pediatric outpatient care in Rwanda without compromising clinical recovery. If scaled more broadly, ePOCT+ has the potential to strengthen adherence to clinical guidelines and support national efforts to promote antibiotic stewardship and combat AMR. Tackling AMR is a strategic priority for the Rwandan health sector, as outlined in the national action plan on AMR [31], the updated national standard treatment guidelines [32], and WHO AWaRe classification [53]—all of which emphasize antibiotic stewardship and rational prescribing. In this context, integrating ePOCT+ and other decision support tools into Rwanda’s national electronic medical record platform in accordance with the national digital health strategy [33], and ensuring that all clinical and reporting functions can be performed through a unified system, represents a critical next step. Such integration could streamline service delivery, improve data quality, and promote more consistent, evidence-based care at scale.

## Supporting information

S1 Annex Study protocol. (PDF)

S2 Annex Statistical analysis plan. (PDF)

S1 CONSORT Checklist CONSORT Checklist. Citation: Hopewell S, Chan AW, Collins GS, Hróbjartsson A, Moher D, Schulz KF, and colleagues. CONSORT 2025 Statement: updated guideline for reporting randomized trials. BMJ. 2025; 388:e081123. https://dx.doi.org/10.1136/bmj-2024-081123. 2025 Hopewell and colleagues. This is an Open Access article distributed under the terms of the Creative Commons Attribution License (https://creativecommons.org/licenses/by/4.0/), which permits unrestricted use, distribution, and reproduction in any medium, provided the original work is properly cited.
(PDF)

S1 Fig Longitudinal plots of study variables. (PDF)

S2 Fig Distribution of clinical outcome across exploratory variables. (PDF)

S3 Fig Antibiotic plots for the intention-to-treat population. (PDF)

S4 Fig Longitudinal plots of antibiotic prescription for individual health facilities. (PDF)

S5 Fig Adjusted relative risks for the clinical failure outcome. (PDF)

S1 Table Sensitivity analysis of antibiotic prescription. (PDF)

S2 Table Exploratory analysis of severe outcomes. (PDF)

S3 Table Results of unadjusted mixed effects logistic regression models. (PDF)

S1 Video Demo of the ePOCT+ algorithm in the medAL-reader application. (MP4)

## Abbreviations

AMR: antimicrobial resistance
CDSAs: clinical decision support algorithms
CI: confidence interval
CRP: C-reactive protein
eCRF: electronic case report form
IMCI: Integrated Management of Childhood Illness
ITT: intention-to-treat
PP: per-protocol
RBC: Rwanda Biomedical Center
SAP: statistical analysis plan
SSA: sub-Saharan Africa
WHO: World Health Organization

## References

[1] Adedokun ST, Yaya S. Childhood morbidity and its determinants: evidence from 31 countries in sub-Saharan Africa. BMJ Glob Health. 2020;5(10):e003109. doi: 10.1136/bmjgh-2020-003109 33046457 PMC7552796

[2] World Health Organization. Primary health care; 2023. Available from: https://www.who.int/news-room/fact-sheets/detail/primary-health-care

[3] Krüger C, Heinzel-Gutenbrunner M, Ali M. Adherence to the integrated management of childhood illness guidelines in Namibia, Kenya, Tanzania and Uganda: evidence from the national service provision assessment surveys. BMC Health Serv Res. 2017;17(1):822. doi: 10.1186/s12913-017-2781-3 29237494 PMC5729502

[4] Lange S, Mwisongo A, Mæstad O. Why don’t clinicians adhere more consistently to guidelines for the Integrated Management of Childhood Illness (IMCI)?. Soc Sci Med. 2014;104:56–63. doi: 10.1016/j.socscimed.2013.12.020 24581062

[5] Kruk ME, Chukwuma A, Mbaruku G, Leslie HH. Variation in quality of primary-care services in Kenya, Malawi, Namibia, Rwanda, Senegal, Uganda and the United Republic of Tanzania. Bull World Health Organ. 2017;95(6):408–18. doi: 10.2471/BLT.16.175869 28603307 PMC5463807

[6] GBD 2019 Human Resources for Health Collaborators. Measuring the availability of human resources for health and its relationship to universal health coverage for 204 countries and territories from 1990 to 2019: a systematic analysis for the Global Burden of Disease Study 2019. Lancet. 2022;399(10341):2129–54. doi: 10.1016/S0140-6736(22)00532-3 35617980 PMC9168805

[7] Boniol M, Kunjumen T, Nair TS, Siyam A, Campbell J, Diallo K. The global health workforce stock and distribution in 2020 and 2030: a threat to equity and “universal” health coverage?. BMJ Glob Health. 2022;7(6):e009316. doi: 10.1136/bmjgh-2022-009316 35760437 PMC9237893

[8] Leslie HH, Spiegelman D, Zhou X, Kruk ME. Service readiness of health facilities in Bangladesh, Haiti, Kenya, Malawi, Namibia, Nepal, Rwanda, Senegal, Uganda and the United Republic of Tanzania. Bull World Health Organ. 2017;95(11):738–48. doi: 10.2471/BLT.17.191916 29147054 PMC5677617

[9] Linder JA, Doctor JN, Friedberg MW, Reyes Nieva H, Birks C, Meeker D, et al. Time of day and the decision to prescribe antibiotics. JAMA Intern Med. 2014;174(12):2029–31. doi: 10.1001/jamainternmed.2014.5225 25286067 PMC4648561

[10] Fink G, D’Acremont V, Leslie HH, Cohen J. Antibiotic exposure among children younger than 5 years in low-income and middle-income countries: a cross-sectional study of nationally representative facility-based and household-based surveys. Lancet Infect Dis. 2020;20(2):179–87. doi: 10.1016/S1473-3099(19)30572-9 31843383

[11] Sulis G, Daniels B, Kwan A, Gandra S, Daftary A, Das J, et al. Antibiotic overuse in the primary health care setting: a secondary data analysis of standardised patient studies from India, China and Kenya. BMJ Glob Health. 2020;5(9):e003393. doi: 10.1136/bmjgh-2020-003393 32938614 PMC7493125

[12] Shekhar S, Petersen FC. The dark side of antibiotics: adverse effects on the infant immune defense against infection. Front Pediatr. 2020;8:544460. doi: 10.3389/fped.2020.544460 33178650 PMC7593395

[13] Duong QA, Pittet LF, Curtis N, Zimmermann P. Antibiotic exposure and adverse long-term health outcomes in children: a systematic review and meta-analysis. J Infect. 2022;85(3):213–300. doi: 10.1016/j.jinf.2022.01.005 35021114

[14] Bell BG, Schellevis F, Stobberingh E, Goossens H, Pringle M. A systematic review and meta-analysis of the effects of antibiotic consumption on antibiotic resistance. BMC Infect Dis. 2014;14:13. doi: 10.1186/1471-2334-14-13 24405683 PMC3897982

[15] Holmes AH, Moore LSP, Sundsfjord A, Steinbakk M, Regmi S, Karkey A, et al. Understanding the mechanisms and drivers of antimicrobial resistance. Lancet. 2016;387(10014):176–87. doi: 10.1016/S0140-6736(15)00473-0 26603922

[16] Antimicrobial Resistance Collaborators. Global burden of bacterial antimicrobial resistance in 2019: a systematic analysis. Lancet. 2022;399(10325):629–55. doi: 10.1016/S0140-6736(21)02724-0 35065702 PMC8841637

[17] World Health Organization. WHO guideline: Recommendations on digital interventions for health system strengthening. World Health Organization; 2018. Available from: https://play.google.com/store/books/details?id=S3Q-zAEACAAJ 31162915

[18] Schmitz T, Beynon F, Musard C, Kwiatkowski M, Landi M, Ishaya D, et al. Effectiveness of an electronic clinical decision support system in improving the management of childhood illness in primary care in rural Nigeria: an observational study. BMJ Open. 2022;12(7):e055315. doi: 10.1136/bmjopen-2021-055315 35863838 PMC9310162

[19] Mitchell M, Hedt-Gauthier BL, Msellemu D, Nkaka M, Lesh N. Using electronic technology to improve clinical care—results from a before-after cluster trial to evaluate assessment and classification of sick children according to Integrated Management of Childhood Illness (IMCI) protocol in Tanzania. BMC Med Inform Decis Mak. 2013;13:95. doi: 10.1186/1472-6947-13-95 23981292 PMC3766002

[20] Sarrassat S, Lewis JJ, Some AS, Somda S, Cousens S, Blanchet K. An Integrated eDiagnosis Approach (IeDA) versus standard IMCI for assessing and managing childhood illness in Burkina Faso: a stepped-wedge cluster randomised trial. BMC Health Serv Res. 2021;21(1):354. doi: 10.1186/s12913-021-06317-3 33863326 PMC8052659

[21] Tan R, Kavishe G, Kulinkina AV, Renggli S, Luwanda LB, Mangu C, et al. A cluster randomized trial assessing the effect of a digital health algorithm on quality of care in Tanzania (DYNAMIC study). PLOS Digit Health. 2024;3(12):e0000694. doi: 10.1371/journal.pdig.0000694 39715234 PMC11666054

[22] Keitel K, Kagoro F, Samaka J, Masimba J, Said Z, Temba H, et al. A novel electronic algorithm using host biomarker point-of-care tests for the management of febrile illnesses in Tanzanian children (e-POCT): a randomized, controlled non-inferiority trial. PLoS Med. 2017;14(10):e1002411. doi: 10.1371/journal.pmed.1002411 29059253 PMC5653205

[23] Shao AF, Rambaud-Althaus C, Samaka J, Faustine AF, Perri-Moore S, Swai N, et al. New algorithm for managing childhood illness using mobile technology (ALMANACH): a controlled non-inferiority study on clinical outcome and antibiotic use in Tanzania. PLoS One. 2015;10(7):e0132316. doi: 10.1371/journal.pone.0132316 26161535 PMC4498627

[24] Tan R, Kavishe G, Luwanda LB, Kulinkina AV, Renggli S, Mangu C, et al. A digital health algorithm to guide antibiotic prescription in pediatric outpatient care: a cluster randomized controlled trial. Nat Med. 2024;30(1):76–84. doi: 10.1038/s41591-023-02633-9 38110580 PMC10803249

[25] Bessat C, Zonon NA, D’Acremont V. Large-scale implementation of electronic Integrated Management of Childhood Illness (eIMCI) at the primary care level in Burkina Faso: a qualitative study on health worker perception of its medical content, usability and impact on antibiotic prescription and resistance. BMC Public Health. 2019;19(1):449. doi: 10.1186/s12889-019-6692-6 31035968 PMC6489291

[26] Pellé KG, Rambaud-Althaus C, D’Acremont V, Moran G, Sampath R, Katz Z, et al. Electronic clinical decision support algorithms incorporating point-of-care diagnostic tests in low-resource settings: a target product profile. BMJ Glob Health. 2020;5(2):e002067. doi: 10.1136/bmjgh-2019-002067 32181003 PMC7050342

[27] Jensen C, McKerrow NH, Wills G. Acceptability and uptake of an electronic decision-making tool to support the implementation of IMCI in primary healthcare facilities in KwaZulu-Natal, South Africa. Paediatr Int Child Health. 2020;40(4):215–26. doi: 10.1080/20469047.2019.1697573 31779539

[28] von Kalckreuth V, Rwandarwacu VP, Cobuccio L, Dusengumuremyi T, Levine GA, Norris M, et al. ePOCT+ Rwanda: a clinical decision support algorithm for managing sick children below 15 years of age in primary healthcare settings. Rwanda J Med Health Sci. 2025;8(1):148–62. doi: 10.4314/rjmhs.v8i1.13 40575279 PMC12188259

[29] Cobuccio LG, Faivre V, Tan R, Vonlanthen A, Beynon F, Barchichat E, et al. medAL-suite: a software solution for creating and deploying complex clinical decision support algorithms. BMC Med Inform Decis Mak. 2025;25(1):249. doi: 10.1186/s12911-025-03077-6 40615862 PMC12226914

[30] Aabenhus R, Jensen J-US, Jørgensen KJ, Hróbjartsson A, Bjerrum L. Biomarkers as point-of-care tests to guide prescription of antibiotics in patients with acute respiratory infections in primary care. Cochrane Database Syst Rev. 2014;(11):CD010130. doi: 10.1002/14651858.CD010130.pub2 25374293

[31] Government of Rwanda. National Action Plan on Antimicrobial Resistance; 2021. Available from: https://cdn.who.int/media/docs/default-source/antimicrobial-resistance/amr-spc-npm/nap-library/rwanda-nap-amr.pdf?sfvrsn=e292aeb1_5&download=true

[32] The guideline for the use of antibiotics in Rwanda. Ministry of Health, Government of Rwanda. 2022. Available from: https://www.moh.gov.rw/index.php?eID=dumpFile&t=f&f=92525&token=65bb1585f37424835c9eeb4c9ad4747b38a80514

[33] Rwanda Ministry of Health. National Digital Health Strategic Plan 2018-2023; 2018. Available from: https://extranet.who.int/countryplanningcycles/sites/default/files/public_file_rep/RWA_Rwanda_Digital-Health-Strategy_2018-2023.Pdf

[34] National Institute of Statistics of Rwanda (NISR). Census Atlas: physical characteristics. 2023. Available from: https://storymaps.arcgis.com/collections/37d4f88579d14f6cb910f988a10fe862?item=3

[35] National Institute of Statistics of Rwanda. The Fifth Rwanda Population and Housing Census; 2022. Available from: https://www.statistics.gov.rw/data-sources/censuses/Population-and-Housing-Census/fifth-population-and-housing-census-2022

[36] Rwanda Biomedical Center. Rwanda Malaria Strategic Plan 2020-2024; 2020. Available from: https://rbc.gov.rw/fileadmin/user_upload/strategy/Rwanda_Malaria_Strategic_Plan_2020-2024.pdf

[37] National Institute of Statistics of Rwanda, Ministry of Finance and Economic Planning. Rwanda demographic and health survey 2014-2015; 2016. Available from: https://play.google.com/store/books/details?id=52N3AQAACAAJ

[38] Rwanda Social Security Board (RSSB). Community based health insurance: coverage trends 2015-2023. Presentation at SDC health face-to-face meeting; 2023 [cited Jan 2024]. Available from: https://www.shareweb.ch/site/Health/Slides%20%20SDC%20Health%20F2F%202023/Rwanda%20Social%20Security%20Board%20RSSB.pdf

[39] Weber DL, Cubaka VK, Kallestrup P, Reventlow S, Schriver M. Rwandan primary healthcare providers’ perception of their capability in the diagnostic practice. Afr J Prim Health Care Fam Med. 2020;12(1):e1–10. doi: 10.4102/phcfm.v12i1.2197 33054271 PMC7565005

[40] Service availability and readiness assessment (SARA). Available from: https://www.who.int/data/data-collection-tools/service-availability-and-readiness-assessment-(sara)

[41] Muff S, Held L, Keller LF. Marginal or conditional regression models for correlated non‐normal data?. Methods Ecol Evol. 2016;7(12):1514–24. doi: 10.1111/2041-210x.12623

[42] Fernandez MAL. Delta method in epidemiology: an applied and reproducible tutorial; 2020. Available from: https://migariane.github.io/DeltaMethodEpiTutorial.nb.html#53_Conditional_risk_ratio_from_a_multivariate_regression_model

[43] Cummings P. The relative merits of risk ratios and odds ratios. Arch Pediatr Adolesc Med. 2009;163(5):438–45. doi: 10.1001/archpediatrics.2009.31 19414690

[44] R Core Team. R: a language and environment for statistical computing. Vienna, Austria: R Foundation for Statistical Computing; 2022. Available from: https://www.R-project.org/

[45] Rotondi MA. Sample size estimation functions for cluster randomized trials. 2023. Available from: https://cran.r-project.org/web/packages/CRTSize/CRTSize.pdf

[46] Langet H, Faye PM, Njiri F, Cicconi S, Levine GA, Glass TR, et al. Effectiveness of introducing pulse oximetry and clinical decision support algorithms for the management of sick children in primary care in Kenya and Senegal on referral and antibiotic prescription: the TIMCI quasi-experimental pre-post study. EClinicalMedicine. 2025;83:103196. doi: 10.1016/j.eclinm.2025.103196 40474998 PMC12140026

[47] Mc Cord KA, Al-Shahi Salman R, Treweek S, Gardner H, Strech D, Whiteley W, et al. Routinely collected data for randomized trials: promises, barriers, and implications. Trials. 2018;19(1):29. doi: 10.1186/s13063-017-2394-5 29325575 PMC5765645

[48] Christie SA, Mbianyor MA, Dissak-Delon FN, Tanjong MM, Chichom-Mefire A, Dicker RA, et al. Feasibility of a cellular telephone follow-up program after injury in sub-Saharan Africa. World J Surg. 2020;44(8):2533–41. doi: 10.1007/s00268-020-05529-8 32347352

[49] Ford I, Norrie J. Pragmatic Trials. N Engl J Med. 2016;375(5):454–63. doi: 10.1056/NEJMra1510059 27518663

[50] Loudon K, Treweek S, Sullivan F, Donnan P, Thorpe KE, Zwarenstein M. The PRECIS-2 tool: designing trials that are fit for purpose. BMJ. 2015;350:h2147. doi: 10.1136/bmj.h2147 25956159

[51] Skivington K, Matthews L, Simpson SA, Craig P, Baird J, Blazeby JM, et al. A new framework for developing and evaluating complex interventions: update of Medical Research Council guidance. BMJ. 2021;374:n2061. doi: 10.1136/bmj.n2061 34593508 PMC8482308

[52] Peters DH, Adam T, Alonge O, Agyepong IA, Tran N. Republished research: implementation research: what it is and how to do it. Br J Sports Med. 2014;48(8):731–6. doi: 10.1136/bmj.f6753 24659611

[53] World Health Organization. The WHO AWaRe (Access, Watch, Reserve) antibiotic book. Genève, Switzerland: World Health Organization; 2022 [cited Jan 2024]. Available from: https://www.who.int/publications/i/item/9789240062382

