# Supplementary material for: Effectiveness of a digital clinical decision support algorithm for guiding antibiotic prescribing in pediatric outpatient care in Rwanda: A pragmatic cluster non-randomized controlled trial
Source: PLoS Med. 2026 Feb 26;23(2):e1004692. doi: 10.1371/journal.pmed.1004692 (PMC12944774; doi:10.1371/journal.pmed.1004692)
Supplement: S1 Annex — (PDF) [file pmed.1004692.s001.pdf]

STUDY PROTOCOL

Dynamic clinical decision support algorithms to manage sick children in primary health care settings in Rwanda (DYNAMIC RW)

**Protocol Number**	752/RNEC/2020		
**Version Number**	2.1	**Document Date**	10-Sep-2021
**Sponsor Contact**	Centre for Primary Care and Public Health (Unisanté)<br>University of Lausanne<br>Bugnon 44 - 1011 Lausanne<br>Switzerland<br>www.unisante.ch		
**Principal Investigator**	Prof. Valérie D'Acremont<br>Centre for Primary Care and Public Health (Unisanté)<br>University of Lausanne<br>Bugnon 44 - 1011 Lausanne – Switzerland<br>+41 79 556 25 51<br>valerie.dacremont@unisante.ch; valerie.dacremont@swisstph.ch		
**Co-Principal Investigator**	Dr. Sabin Nsanzimana<br>Rwanda Biomedical Centre<br>KG 17 Ave, Ramera, Kigali, Rwanda<br>+250 788 752 475<br>sabin.nsanzimana@rbc.gov.rw		
**Funding Agency**	- Fondation Botnar, St. Alban-Vorstadt 56 - 4052 Basel – Switzerland<br>- Swiss Development Cooperation, Bern – Switzerland		

The information contained in this document is confidential. It is intended solely for the Investigators, potential Investigators, consultants, or applicable Independent Ethics Committees and Regulatory Authorities. It is understood that this information will not be disclosed to others without prior written authorization from the Sponsor, except where required by applicable local laws.

I. List of Investigators and other persons involved

Names	Institution	Position	Function in study
Prof. Valérie D'Acremont	Unisanté	Head of Digital and Global Health, Department of Training, Research and Innovation	PI, overall project coordinator
	Swiss TPH	Scientific Group Leader, Medical Department	
Dr. Sabin Nsanzimana	RBC	Director General	Co-PI, Advisor on national health system relevance
Dr. Alexandra V. Kulinkina	Swiss TPH	Project Leader, Swiss Centre for International Health	Co-investigator, Project coordinator
Cassien Havugimana	Rwanda office Swiss TPH	Kigali regional office coordinator	Co-investigator, Project implementer
Dr. Ludovico Cobuccio	Unisanté and Swiss TPH	Research physician	Clinical coordinator
Dr. Theophile Dusengumuremyi	Rwanda office Swiss TPH	Research physician	Clinical implementer
Francine Bayisenge	Rwanda office Swiss TPH	Social Scientist	Social scientist
Vincent Faivre	Unisanté	Deputy head of IT department	IT and data coordinator
Serge Zimulinda	Rwanda office Swiss TPH	IT Coordinator	IT implementer
Gilbert Rukundo	Rwanda office Swiss TPH	Data Manager	Data manager
Martin Norris	Swiss TPH	Project Manager	Implementation project manager
Aicha Yusuf	Rwanda office Swiss TPH	Assistant Project Manager	Project implementer
Dr. Victor Rwandarwacu	Rwanda office Swiss TPH	Assistant Research Clinician	Assistant Research Clinician
Dr. Marie-Annick Le Pogam	Unisanté	Associate Clinician	Advisor on statistical methods
Alan Vonlanthen	Unisanté	Project Manager, IT department	IT project manager
Dr. Mary-Anne Hartley	EPFL and Unisanté	Scientific Project Leader, Digital and Global Health, Department of Training, Research and Innovation	Machine Learning coordinator
Prof. Kaspar Wyss	Swiss TPH, Basel	Deputy Director and Head of Department, SCIH	Advisor on health systems and stakeholder engagement
Dr. Felix Sayinzoga	RBC	Maternal, Child and Community Health Division Manager	Advisor on national child health context
Dr. Pacifique Ndishimye	RBC	Clinical Research Opportunities Coordinator	Advisor on research and innovation
Noella Bigirimana	RBC	Division Manager for Research, Innovation and Data Science	Advisor on research and innovation
Dr. Albert Tuyishime	RBC	Head of the Institute of HIV/AIDS Disease Prevention and Control	Advisor on national data systems and HMIS

Project Location: Western Province, Rwanda
Projection duration: Project starts on 01-Sep-2021; Project ends on 31-Dec-2022

II. Signatures

The signatures below confirm agreement by the individuals authorized by the sponsor and principal participating institutions at the sites that the study will be conducted in compliance with protocol version 2.1 dated 10-Sep-2021.

Principal Investigator

Signature		10-Sep-2021
Name	Prof. Valérie D'Acremont	Date of Signature
Job title	Head, Digital and Global Health, Department Training, Research and Innovation	
Institution	Centre for Primary Care and Public Health, University of Lausanne	
Address	Bugnon 44 - 1011 Lausanne, Switzerland	
Phone	+41 79 556 25 51	

Co-Principal Investigator

Signature		10-Sep-2021
Name	Dr. Sabin Nsanzimana	Date of Signature
Job title	Director General	
Institution	Rwanda Biomedical Centre	
Address	KG 17 Ave, Ramera, Kigali, Rwanda	
Phone	+250 788 752 475	

Co-Investigator

Signature		10-Sep-2021
Name	Alexandra Kulinkina	Date of Signature
Job title	Project Leader, Medical Department and Swiss Centre for International Health	
Institution	Swiss Tropical and Public Health Institute	
Address	Socinstrasse 57 - 4051 Basel, Switzerland	
Phone	+41 79 555 90 46	

Co-Investigator

Signature		10-Sep-2021
Name	Cassien Havugimana	Date of Signature
Job title	Regional Coordinator	
Institution	Local/Regional Office of the Swiss Tropical and Public Health Institute	
Address	Kigali Heights, West wing, 5th Floor, KG 7 Ave Kigali, Rwanda	
Phone	+250 788 313 954	

III. Abbreviations / Glossary of terms

AMR	Antimicrobial resistance
CDSA	Clinical decision support algorithm
DHMT	District Health Management Team
CRP	C-reactive protein
DHIS2	District Health Information System 2
EPFL	École Polytechnique Fédérale de Lausanne
ePOCT+	Electronic point of care tool plus
FGD	Focus group discussion
HF	Health facility
HW	Health worker
HMIS	Health management information system
IMCI	Integrated management of childhood illness
IT	Information technology
M&E	Monitoring and evaluation
ML	Machine learning
MoH	Ministry of Health
PHC	Primary health care
POC	Point-of-care
RBC	Rwanda Biomedical Centre
RDT	Rapid Diagnostic Test
RW	Rwanda
Swiss TPH	Swiss Tropical and Public Health Institute
Unisanté	Centre for Primary Care and Public Health (University of Lausanne)
WHO	World Health Organization

IV. Synopsis

**Study Title**	**Dynamic clinical decision support algorithms to manage sick children in primary health care settings in Rwanda**
**Short Title**	**DYNAMIC RW**
**Study Category**	**Implementation study to support clinical decision making and disease surveillance in sick children**
**Study rationale**	Children are a well-recognized vulnerable population that still suffers from high rates of acute infectious diseases and preventable deaths due to inadequate clinical management at the PHC level. This is especially true in the fragile health systems of sub-Saharan Africa, where under-five mortality is ten times that of high-income countries. In these settings, health professionals rely on generic guidelines, such as the WHO IMCI booklet (WHO 2014A), while often lacking diagnostic tools, skills, and supportive supervision, which can lead to misdiagnoses and inappropriate prescription practices. Clinically validated POC diagnostic tests are often not available and static guidelines like IMCI can become rapidly outdated due to new evidence and changing epidemiology. When an epidemic arises, such static and generic guidelines can even become dangerous if the event is not detected on time and integrated into the recommendations.<br>In the absence of diagnostic tools, clear guidance, and supervision, it is challenging for HWs to confidently identify the minority of children with bacterial infections needing antibiotic treatment among the predominant cases of self-limiting diseases. In fact, nine out of ten febrile children attending PHC facilities typically receive antibiotics, as compared to the one or two who actually need them. Antibiotic over-treatment hinders the development of the natural, protective bacterial gut flora, which is an important immunological barrier. The tremendous overuse of antibiotics, especially in children at the PHC level where most patient encounters occur, contributes to the global spread of antibiotic resistance, which is a major public health threat, estimated to be responsible for up to 10 million deaths per year by 2050.<br>Misdiagnoses and poor prescribing practices have consequences that reach beyond the patient. They increase re-attendance rates, further congesting PHC facilities and accruing economic losses not only for families but for the entire health system. Systematic errors in patient-level data accumulate, and as they are aggregated in HMIS to measure population-level indicators, they have the potential to bias the statistics used to prioritize public health interventions and, importantly, identify epidemics.<br>The WHO has identified digital health interventions and predictive tools in primary care as key accelerators in achieving the 2030 Sustainable Development Goal 3 of ensuring good health and well-being for all. New simple and cheap technologies, such as mobile devices, coupled with the advances in computing and data science, could help mitigate several of the aforementioned challenges. Two versions of the CDSA for managing sick children have undergone rigorous evaluations, demonstrating that electronic algorithms can significantly reduce antibiotic prescriptions and improve patient outcomes in controlled research conditions.<br>• The first-generation algorithm called ALMANACH (based largely on IMCI) was tested in Tanzania in 2010-2011, achieving improved clinical cure (from 92% to 97%) and a decrease in antibiotic prescription (from 84% to 15%) as compared to routine care. ALMANACH also led to more consistent clinical assessments without taking more time than a conventional consultation and was perceived by clinicians as “a powerful and useful” tool.<br>• The second-generation algorithm called ePOCT was trialed in Tanzania in 2014-2016. In addition to symptoms and signs, it made use of several POC tests to help detect children with severe infections requiring hospital-based treatment (oximetry and hemoglobin level) and/or children with serious bacterial infection (CRP). The use of ePOCT resulted in higher clinical cure (98%) as compared to ALMANACH (96%) and routine care (95%). The algorithm also further reduced antibiotic

	prescription to 11%, as compared to 30% with the use of ALMANACH and 95% in routine care.<br>The proposed intervention includes a third-generation algorithm called ePOCT+. Its medical content has been expanded from the prior versions to cover a wider pediatric age range and clinical spectrum. The implementation of ePOCT+ will be accompanied by other electronic tools to facilitate uptake and integration into the health system and ensure sustainability. The additional features of the intervention include: a) e-learning modules to enable continuing education for HWs; b) an electronic platform that will use patient-level data to enable targeted supportive supervision of HWs and enhance M&E capacity at the local level; and c) an electronic platform that will allow medical experts in charge of clinical guidelines with no programming skills to adapt the clinical algorithm to changing epidemiological conditions.<br>The goal of the DYNAMIC project is to demonstrate the potential of digital CDSAs like ePOCT+ to improve care through further validation and implementation studies in different settings and in less research-like conditions (Figure 2). Implementation research will be conducted throughout the project, evaluating its feasibility, cost-effectiveness and impact, and to understand facilitators and barriers for further scale-up.<br>The project is aligned with Rwanda's national priorities to promote research in primary health to increase quality, demand and accessibility of health care for all. Furthermore, the 4th Health Sector Strategic Plan emphasizes the expansion of ICT and e-health within the health sector and increasing the availability of web-based training and educational opportunities for the health workforce. It is the intention of the DYNAMIC project to work within the current e-health landscape of Rwanda and to explore interoperability and data exchange with medical records systems deployed in the PHC setting (such as OpenMRS) and other platforms to improve the workflow of HWs and enhance the national data systems overall.
**Study objectives**	The goal of this study is to mitigate Antimicrobial Resistance (AMR) using a novel dynamic ePOCT+ tool that helps front-line HWs manage sick patients at primary care level, enhanced by additional digital platforms to promote uptake, sustainability, and integration of the tool into the health system.<br>More specifically, this study seeks to:<br>**Objective 1:** Improve the integrated management of children with an acute illness (more specific and diverse diagnoses; reduced antibiotic prescriptions) through the provision of an electronic CDSA (ePOCT+) to clinicians working at primary care level.<br>**Objective 2:** Improve the diagnostic and prognostic accuracy of the clinical algorithm and allow it to adapt to spatiotemporal variations in epidemiology and resources, based on the data generated through the ePOCT+ tool, analyzed using machine learning and checked by clinical experts.<br>**Objective 3:** Enhance the health management information system for monitoring and evaluation and conducting supportive supervision (increased number of meaningful indicators that are reviewed and actioned regularly) in HFs using the clinical data generated by the ePOCT+ tool and enhanced by additional data analysis and visualization dashboards.<br>**Objective 4:** Create a framework for the implementation of dynamic clinical decision support algorithms and supporting tools, for large-scale, sustainable, and clinically responsible use of machine learning and data science.
**Intervention**	The intervention will include four activities that are in line with the objectives of the study and supported by specific electronic tools:<br>***A)*** *Acutely ill children will be assessed, diagnosed and treated by routine clinicians guided by the* ***ePOCT+*** *application, which will be loaded on a tablet or PC.*

	The intervention will be undertaken in routine PHC facilities in Rwanda and entails the implementation of the ePOCT+ tool for patient assessment and management, with the provision of associated POC diagnostic tests. Healthcare providers will use ePOCT+ throughout their medical consultations for children aged 0-14 years (inclusive) presenting with an acute medical or surgical problem. The current CDSA of ePOCT+ is based on international guidelines and protocols (WHO Pocket Book of Hospital Care for Children 2013; WHO IMAI guidelines 2009) and is being updated based on the Rwandan national guidelines for pediatric clinical management (IMNCI booklet; Rwanda National Neonatology clinical treatment guidelines, National Guidelines for Prevention and Management of HIV and STIs, ETAT guidelines, Treatment guidelines, etc.). Specifically, the tool will guide clinicians on:<br>i) Decision on immediate referral to a higher level facility<br>ii) Medical history (questions to ask caregivers and/or children)<br>iii) Physical exam (signs to look for)<br>iv) POC diagnostic tests (that should be performed in a given clinical context)<br>v) Diagnoses<br>vi) Treatments (including medicines and supportive treatments)<br>vii) Counseling (explanation of final diagnoses, treatments, and follow-up)<br><br>***B)*** *The algorithm will be regularly updated according to trends in the collected data. The modifications will be designed by clinical experts based on the data collected during the study. Approved changes will then be integrated into the algorithm using the* ***medAL-creator*** *tool that allows physicians without any IT/programming skills to modify the decision trees.*<br><br>During Phase 2 of the study, the algorithm will be modified according to trends in the collected data detected using ML or conventional statistical methods. The modifications will be designed and validated by Rwandan clinical experts. Approved changes will then be integrated into the algorithm using medAL-creator and a new version of the ePOCT+ algorithm released. The new version will then be deployed onto the tablets or PCs of the relevant HWs (for example of a certain region where an epidemic is occurring) with a message explaining the rationale and evidence behind the change in the algorithm.<br><br>***C)*** *Clinical competencies of health workers will be targeted through supportive supervision from the DHMT using the* ***medAL-monitor*** *tool and additional training through the* ***e-learning*** *modules.*<br><br>Targeted supervision to HFs by the DHMT will be organized based on the quality of care indicators calculated in real time from the uploaded data and made available through a web-based dashboard called medAL-monitor. Personalized feedback to HWs and persons in charge of HFs will be provided for self-auditing and e-learning platform for additional skill building opportunities.<br><br>***D)*** *M&E capacity of the intervention districts will be enhanced through the* ***medAL-monitor/medAL-outbreak*** *tools.*<br><br>Dashboards created with ePOCT+ data will enable DHMT to monitor certain health indicators over time, be alerted if indicator values are higher than expected for a given month in a given location, suggesting a potential outbreak (or another change in the health system), and be able to decide if a response is needed.
**Study design**	The primary intervention study will be conducted using a parallel-group cluster non-randomized controlled superiority trial study design implemented in two phases. Additional mixed-methods operational research will take place throughout the intervention period.<br><br>**Phase 1: parallel cluster randomized controlled study in 32 health facilities**<br><br>The ePOCT+ tool will be introduced sequentially using in three implementation blocks of 10-12 (half intervention and half control) facilities. Each of the parallel cluster study (implementation block) will last for approximately four months, with the first month serving as a phase-in period. The aim is to compare the effect of using ePOCT+ with

	routine case management of sick children on antibiotic prescriptions by HWs. Due to the pragmatic nature of the study, the design is adaptive, in that changes to the implementation may be incorporated during each cluster study to account for issues encountered in monitoring data and feedback from the field. These changes apply to the implementation context and exclude significant adaptations of the algorithm.<br>**Phase 2: transformation of ePOCT+ into a dynamic algorithm**<br>In Phase 2, the same outcome indicators as in Phase 1 will be measured and evaluated over time to monitor longitudinal changes in the effectiveness of the intervention. The medical content of the algorithm will not be fixed anymore, but rather modifiable. Each potential modification will first be evaluated by the Rwandan clinical expert group for its clinical coherence, safety and potential benefit and then applied to the retrospective data. If retrospective analyses confirm a clinically relevant positive impact (or resource saving), the modification will be implemented in the relevant locations and/or patient sub-groups.<br>**Mixed-methods operational research:**<br>Throughout the pilot and Phases 1 and 2, mixed-methods operational research will be conducted in HFs and communities, as well as at district and national levels, to understand the contextual factors that facilitate or impede the implementation, sustainability and scale-up of ePOCT+ and other digital health interventions in PHC settings. Examples of such factors may include socioeconomic and ecological profiles of communities, working conditions of the clinicians in the HFs, among others. The studies will include health facility assessments, cross-sectional quality of care surveys, other quantitative surveys, in-depth interviews and focus group discussions with health care providers, caregivers, patients, community groups, and actors within the health system at district and national levels. Economic and ecological impacts of the project will also be assessed.
**Study site**	The study will be conducted in Nyamasheke and Rusizi districts in the Western Province of Rwanda.
**Study population**	All children aged 1 day to 14 years (inclusive) with an acute condition who present to the participating PHC facilities will be included.<br>HWs attending to these children, caregivers of these children, communities where these children live, and key actors of the health system (DHMTs, computer scientists and medical experts at national level) will be included in observational mixed-methods studies.
**Outcomes**	**Objective 1: Improve integrated management of sick children**<br>**Primary outcome measure** (parallel cluster study in Phase 1)**:**<br>- % of children prescribed an antibiotic at initial consultation (as reported by HWs) in intervention facilities as compared to control facilities.<br>**Secondary outcome measure** (parallel cluster study in Phase 1)**:**<br>- % of children cured at day 7* (according to caregivers contacted through a phone call or SMS or home visit) in intervention facilities as compared to control facilities.<br>* non-referred secondary hospitalizations occurring within 7 days will however be considered as clinical failures even if the child is already cured at day 7<br>**Primary outcome indicators** (monitored over time and space in Phase 2)**:**<br>- % of children prescribed an antibiotic at initial consultation<br>**Secondary outcome indicators** (monitored over time and space in Phase 2)**:**<br>*Clinical outcome:*<br>- % of children cured at day 7<br>- % of children with non-referred secondary hospitalization by day 7<br>- % of children who have died by day 7<br>*Re-attendance visits:*

	- % of children with ≥1 unscheduled re-attendance visits at any HF by day 7<br><br>*Primary referrals:*<br>- % of children referred to hospital or admitted to inpatient ward at a health center at initial consultation<br>- % of children who were hospitalized among those referred (i.e. referral completed)<br>*Appropriate case management for malaria at initial consultation:*<br>- % of suspected cases tested for malaria<br>- % of malaria positive children prescribed an antimalarial<br>- % of malaria negative children prescribed an antimalarial<br>- % of untested children prescribed an antimalarial<br>*Appropriate case management for other conditions at initial consultation:*<br>- % of key symptoms and signs checked and diagnostic tests performed by HWs<br>- Distribution of final diagnoses (including their severity) made by HWs<br>- Concordance between treatment prescribed and final diagnoses made by HWs<br>- Concordance between final diagnoses as proposed by ePOCT+ and final diagnoses by HW *(intervention HFs only)*<br>**Objective 2: Improve the algorithm and adapt it to spatiotemporal variations**<br>- Number of positive impact modifications in the algorithm identified by ML analyses<br>- Number of modifications implemented, by type of impact (better clinical cure, less severe clinical outcomes, less medicines or diagnostic tests needed, shorter consultation time)<br>**Objective 3: Enhance M&E and supportive supervision**<br>- Number of M&E indicators based on individual data that can be visualized through the medAL-monitor/medAL-outbreak dashboards<br>- Number of supervision and mentorship visits to HFs facilitated by the medAL-monitor tools<br>- Number of HWs reporting having used the medAL-monitor tool for self-auditing their clinical practices<br>- Number of HWs and members of DHMT reporting a positive experience with the medAL-monitor/medAL-outbreak tools<br>**Objective 4: Create a supportive environment for the use of clinical algorithms and supporting tools**<br>- Number of sick children attending primary care facilities managed by HWs using the electronic ePOCT+ tool<br>- % of HWs able to assess key clinical signs<br>- Frequency of use of the medAL-monitor/medAL-outbreak tool by district team members<br>- Perception by HWs of the ePOCT+ tool and intervention and the number of improvements they propose for the tool or intervention<br>- Perception by caregivers and community members of the intervention and the number of improvements they propose<br>- Perception by district team members of the medAL-monitor/medAL-outbreak tool and the number of improvements they propose for the tool<br>- Number of medical experts able to modify a clinical algorithm using medAL-creator<br>- Number of computer scientists able to maintain the software and the IT infrastructure for clinical algorithms<br>- Number of data scientists able to analyze clinical data using ML<br>- Patient and provider expenditures related to care of acutely ill children included in the study

	- Carbon footprint of the intervention or carbon-saving due to the intervention
**Procedures and measurements**	**Objective 1: Improve integrated management of sick children**<br>In Phase 1, eligible HFs will be grouped into three implementation blocks of 10-12 health facilities (half intervention and half control). Randomization will not be done for reasons of feasibility and high chance of contamination if control and intervention facilities are mixed and in close proximity to each other as randomized. Therefore, boundaries between groups of intervention and control facilities will be delineated geographically.<br>Sick children will be enrolled throughout Phase 1, as new blocks of facilities are introduced every four months and control facilities are converted into intervention facilities. Children enrolled in intervention facilities will be managed using ePOCT+, with all data associated with the consultation being captured electronically. Children enrolled in control facilities will be assessed, diagnosed and treated as per routine protocols by clinicians. A similar but simplified tablet or PC application will also be used in control facilities to record only outcome measure related data using an electronic case report form.<br>Primary outcome measure: For measuring antibiotics prescribed at initial consultation, the treatment data recorded in the tablets or PCs by HWs throughout the study will be compared between intervention and control facilities, captured electronically in both facility types.<br>Secondary outcome measure: For assessing clinical cure at day 7, a simple questionnaire (asking if the child is still sick, was hospitalized secondarily, or died) will be administered to all caregivers by phone.<br>In **Phase 2**, all HFs will be using ePOCT+. Indicators monitored over time and space will be measured the same way as in Phase 1, except that clinical cure, secondary hospitalizations and deaths may not be collected or will be assessed through an automated phone call or SMS sent to caregivers using the same simple questionnaire (resource dependent).<br>Secondary analyses will be used to assess differences in the quality of the consultation made by HWs, including the rational use of antimicrobials (antibiotics and antimalarials). Two sources of data will be used and compared between intervention and control facilities:<br>- the final decision on referral, tests performed, final diagnoses retained and treatment prescribed will be recorded in the tablets or PCs by HWs at initial consultations and re-attendance visits.<br>- during a cross-sectional quality of care survey, observers in the consultation room will record key symptoms and signs assessed by HWs, diagnostic tests requested, final decision on referral, final diagnoses retained and treatment prescribed.<br>**Objective 2: Improve the algorithm and adapt it to spatiotemporal variations**<br>In Phase 2 of the study, the algorithm will be improved in terms of diagnostic and prognostic accuracy and adapted to local fluctuations in epidemiology, demography, geography and resource availability. Each potential modification will first be evaluated by the Rwandan clinical expert group for its clinical coherence, safety and potential benefit, and then applied to the retrospective data. If these analyses confirm a clinically relevant positive impact, the change in the algorithm will be implemented in all relevant locations/patient sub-groups and monitored based on the indicators relevant to the specific change in the algorithm collected throughout the study through the tablets.<br>**Objective 3: Enhance M&E and supportive supervision**<br>Patient-level data will also enhance M&E activities and supportive supervision of the HWs through the medAL-monitor/medAL-outbreak platform. Clinicians will use data generated from their own consultations to self-audit their clinical practices by monitoring quality of care indicators in the medAL-monitor dashboards and improve their skills in specific areas using the e-learning platform. Similarly, members of the DHMT will use medAL-monitor to identify and provide support to HFs in need of additional training and mentorship. In time, this enhanced ability to capacitate HWs and

	improve quality of care should become evident in the trends in the M&E indicators displayed in medAL-monitor and subsequently in the national HMIS. Syndromic surveillance trends will be monitored by the DHMT using the complementary medAL-outbreak dashboard.<br>**Objective 4: Create a supportive environment for the use of clinical decision support algorithms and supporting tools**<br>During Phases 1 and 2, mixed-methods operational research will be undertaken to assess facilitating factors and barriers to ePOCT+ deployment. HWs, caregivers, community members and district health managers will be interviewed to assess the perception and acceptability of ePOCT+ and related tools.<br>Sustainability of the intervention will be evaluated by assessing the level of local human capacities that will have been reached at the end of the study in terms of: 1) clinical competencies of HWs; 2) clinical algorithm creation by medical experts; 3) software and IT infrastructure maintenance by computer scientists; 4) ML analysis by data scientists.<br>Finally, cost analyses will be performed using ML on the data collected during Phases 1 and 2, assigning a cost to each procedure performed or medication prescribed during consultations, as well as to the transport to come to the HF or to the hospitalization of the child if any, or of income lost. An ecological assessment of the intervention will also be performed to calculate the carbon footprint or carbon-saving of the intervention.
**Statistical considerations**	For the parallel cluster study in Phase 1, the primary analysis will evaluate whether the use of ePOCT+ for case management of sick children in PHC facilities results in decreased antibiotic prescriptions (superiority analysis) at the initial consultation. Superiority in antibiotic prescription is defined as a relative decrease of ≥25% in the proportion of children prescribed an antibiotic. We estimated a cluster size of 660 patients (monthly utilization of 220 – accounting for up to 30% non-adherence from 315 – over 3 months) and an intraclass correlation coefficient of 0.025. To have 80% power to detect a 25% reduction in antibiotic prescription (e.g. from 35% to 26%), for a one-sided hypothesis test at alpha of 0.025, we would require a minimum of 11 clusters per arm. With 16 clusters (or HFs) per arm, over 3 months, we expect a total of 10,500 participants per arm, this sample size is sufficient to test for non-inferiority in the secondary outcome (clinical cure at day 7) between the control and intervention groups.<br>We will test for significant difference in the primary outcome of antibiotic prescription between study arms by comparing proportions and their 95% confidence intervals. Primary analysis of superiority will be performed according to the intention-to-treat principle. The results will be stratified by sex, age group (young infants ages 1 to 59 days, children aged 2 months to 5 years, and children aged 5 to 14 years), and select clinical variables (respiratory symptoms, fever without clinical source, gastrointestinal complaints, skin problems, ear and throat problems, anemia, and malnutrition).<br>At the estimated utilization of the PHC facilities in Nyamasheke and Rusizi districts, we anticipate that approximately 150,000 consultations will be conducted using ePOCT+ throughout the study.
**Study duration and schedule**	Pilot phase: April 2021 – August 2021<br>Phase 1 (data collection): September 2021 – April 2022<br>Phase 2 (data collection): June 2022 – December 2022<br>Implementation research (data collection): October 2020 – October 2022<br>Sustainability assessment: August 2022 – December 2022
**Ethical statement**	The research project will be carried out in accordance with the research plan outlined in this protocol and with principles enunciated in the current version of the Declaration of Helsinki, Essentials of Good Epidemiological Practice issued by Public Health Switzerland (EGEP) as well as all national legal and regulatory requirements as applicable.

TABLE OF CONTENTS

1 Background and rationale 15
1.1 Management of sick children 15
1.2 Over-prescription of antimicrobials 15
1.3 Training and supervision of health workers 16
1.4 Monitoring and evaluation 16
1.5 Clinical decision support algorithms 17
1.6 Proposed intervention 18
1.7 Anticipated impact 20
2 Study objectives and theory of change 21
2.1 Objective 1 21
2.2 Objective 2 21
2.3 Objective 3 21
2.4 Objective 4 21
2.5 Theory of change 22
3 Description of the intervention 22
3.1 Management of sick children by health workers using ePOCT+ 22
3.2 Modification of ePOCT+ to improve accuracy and adapt to epidemiological changes 26
3.3 Enhanced clinical competencies of health workers 26
3.4 Enhanced monitoring and evaluation 27
4 Study design 29
4.1 Primary intervention 29
4.2 Mixed-methods operational research 30
4.2.1 Baseline health facility assessment 30
4.2.2 Cross-sectional quality of care survey 30
4.2.3 In-depth interviews, focus group discussions and surveys with health workers 31
4.2.4 In-depth interviews, focus-group discussions and surveys with caregivers 31
4.2.5 Focus group discussions with selected community groups 31
4.2.6 In-depth interviews with actors within the health system 31
4.2.7 Economic and ecological impact evaluations 32
5 Study area 32
5.1 Study sites 32
5.2 Selection of health facilities 35
6 Study population 36

6.1 Sick children attending PHC facilities 36
6.2 Other stakeholders and/or beneficiaries 37
7 Outcome measures and indicators 37
7.1 Outcome measures and indicators for Objective 1: Improve the integrated management of sick children 37
7.2 Indicators for Objective 2: Improve the algorithm and adapt it to spatiotemporal variations 39
7.3 Indicators for Objective 3: Enhance M&E and supportive supervision 39
7.4 Indicators for Objective 4: Create a supportive environment for the use of clinical algorithms and supporting tools 39
8 Study procedures 39
8.1 Objective 1: Improve the integrated management of sick children 40
8.1.1 Recruitment, screening and informed consent procedure 40
8.1.2 Management of sick children during the initial consultation 41
8.1.3 Re-attendance visits in case of persisting or worsening of symptoms 42
8.1.4 Training of health workers 42
8.1.5 Supervision of health workers by the DHMT 43
8.1.6 Measurement of primary and secondary outcomes 43
8.1.7 Unique study identification number 44
8.2 Objective 2: Adapt the algorithm to epidemiological and demographic conditions 44
8.3 Objective 3: Enhance M&E and supportive supervision 45
8.4 Objective 4: Create a supportive environment for the use of clinical algorithms and supporting tools 45
8.4.1 Stakeholder engagement 45
8.4.2 Capacity building 45
8.4.3 Development of frameworks for future scale-up of the e-POCT+ intervention 46
9 Statistical methods 46
9.1 Sample size calculations 46
9.2 Statistical analysis for the primary outcome measure in the parallel cluster study 47
9.3 Machine learning analyses 47
9.3.1 Supervised classification and correlation 47
9.3.2 Anomaly detection 48
9.4 Analyses of data from mixed-methods studies 48
9.5 Estimated number of beneficiaries 48
10 Information technology, data management and monitoring 49
10.1 Overall IT solution 49

10.2 Data flow, storage and transfer 50
10.3 Data security and privacy 51
10.4 Data quality assurance 52
11 Ethical considerations 52
11.1 Participant information and consent 52
11.2 Risk-benefit assessment 52
11.3 Internal monitoring 54
11.4 Premature termination of study 54
11.5 Local regulations, declaration of Helsinki and protocol amendments 54
11.6 Alignment of the project with national priorities 55
12 Organization and timelines 55
12.1 Organizational structure 55
12.2 Roles of investigators and collaborators 56
12.3 Timelines 58
13 Capacity building, knowledge transfer and dissemination plans 58
14 Budget 60
15 References 61
16 Appendices 65

1 BACKGROUND AND RATIONALE

1.1 Management of sick children

Children are a well-recognized vulnerable population that still suffers from high rates of acute infectious diseases and preventable deaths due to inadequate clinical management at the PHC level. In PHC settings, health professionals rely on generic guidelines, such as the WHO IMCI booklet (WHO 2014A), while often lacking diagnostic tools, skills, and supportive supervision, which can lead to misdiagnoses and inappropriate prescription practices. This is especially true in the fragile health systems of sub-Saharan Africa, where under-five mortality is ten times that of high-income countries. Rwanda has made significant progress in reducing under-5 mortality, with recent mortality rate estimates (35 deaths per 1,000 live births) less than half those of sub-Saharan Africa (78 deaths per 1,000 live births) (World Bank 2018). Nonetheless, further progress is possible, with children still dying of preventable causes such as pneumonia, meningitis, malaria and acute respiratory infections according to a recent verbal autopsy study (Gupta et al. 2018).

The IMCI strategy, used in the PHC outpatient setting, with its integrative disease management approach, remains very relevant today. Yet its implementation has faced major challenges, largely related to format and content-related shortcomings (Rambaud-Althaus et al. 2017; Lange, Mwisongo, and Mæstad 2014; Baiden et al. 2011). For example, the IMCI booklet requires integrating information from about 20 pages during a consultation, which is a great challenge for HWs. Additionally, the IMCI algorithm is over-reliant on clinical signs and symptoms, which consume the majority of the consultation time and inherently lack diagnostic accuracy in children (Keitel et al. 2017; Rambaud-Althaus et al. 2015). Diagnostic POC tests that can help detect children with severe illness have not been incorporated into the IMCI algorithm.

Furthermore, static guidelines like IMCI tend to become rapidly outdated. With revision cycles of 5-10 years, they cannot keep up with new evidence and epidemiological changes. They tend to be too generic, in that only a single version exists for an entire country, while each region faces different types of diseases and levels of transmission. When an epidemic arises, such static and generic guidelines can even become dangerous: existing recommendations may become inapplicable with changing disease patterns. As a result, HWs may put standard guidelines aside to focus on the disease outbreak. For example, the risk of malaria deaths increased during the West African Ebola epidemic as HWs focused on Ebola case management (Walker et al. 2015).

1.2 Over-prescription of antimicrobials

In the absence of diagnostic tools, clear guidance, and supervision, it is challenging for HWs to confidently identify the minority of children with bacterial infections needing antibiotic treatment among the predominant cases of self-limiting diseases (Rambaud-Althaus et al. 2017). Thus, they tend to over-prescribe antibiotics - and sometimes also antimalarials - “to be on the safe side” (Hopkins et al. 2017, Fink et al. 2019). In fact, nine out of ten febrile children attending PHC facilities typically receive antibiotics, as compared to the one or two who actually need them (D'Acremont et al. 2014). According to the most recent Demographic and Health Survey (DHS), approximately 49% of under 5 children with an acute respiratory infection (ARI), 39% with fever, and 10% with diarrhea received an antibiotic from a community health worker or a facility-based healthcare provider (DHS 2014-2015). Despite this high-volume and indiscriminate antibiotic treatment, mortality from childhood infections remains high. This is mainly because children with severe infections, which can be caused by viral, parasitic, or bacterial pathogens, are missed (Keitel et al. 2017). The over-reliance on antibiotics has also resulted in the neglect of other important supportive treatments for severe infections (e.g. oxygen therapy for viral bronchiolitis). Antibiotic over-treatment hinders the development of the natural, protective bacterial gut flora, which is an important immunological barrier (Benoun et al. 2016) and has been shown to be associated with cognitive neurological and behavioral development (Carlson et al. 2018, Loughman et al. 2020).

The tremendous overuse of antibiotics, especially in children at the PHC level where most patient encounters occur, contributes to the global spread of antibiotic resistance (AMR), which is a major public health threat, estimated to be responsible for up to 10 million deaths per year by 2050 (Holmes et al. 2016, Fink et al. 2019). According to the WHO, AMR is rising to dangerously high levels, especially in low-resource settings. Limited AMR surveillance data for Rwanda exists; however several studies conducted in tertiary and referral hospitals suggest widespread antimicrobial resistance among bacterial infections (Ntirenganya et al. 2015, Sutherland et al. 2019) with decreasing susceptibility trends for several antibiotic treatments between 2009 and 2013 (Carroll et al. 2016). Interestingly, a recent study among healthcare students in Rwanda found that the majority (up to 96%) demonstrated good knowledge of antibiotics, agreed that inappropriate use of antibiotics could lead to AMR, and that it was important for healthcare students to be knowledgeable about AMR. However, 38% did not believe that antibiotics were overused in Rwanda, approximately 20% believed that antibiotics are useful for viral infections like the flu, and 40% reported taking antibiotics without a prescription (Nisabwe et al. 2020). Antimicrobial stewardship among prescribers in outpatient settings, in combination with further education of healthcare professionals and the lay public are urgently needed to mitigate AMR.

1.3 Training and supervision of health workers

Current standards of training and supervision for HWs have much potential for further improvement. Continuous education still largely consists of one-time, off-site, didactic classroom-based instruction, which often lack relevance to the individual HW (Kiplagat et al. 2014) and are proven to have limited effectiveness in improving and sustaining HW competencies (Rowe et al. 2009). Rather, simulation and case-based learning at the point of care (Bluestone et al. 2013) and group problem solving (Rowe et al. 2018) are better suited at helping HWs put their knowledge into practice. To be most effective, on-the-job training and mentorship must be embedded in a broader quality improvement plan (WHO 2006, Rowe et al. 2009). This training approach focused on clinical simulation with hands-on exercises has already been recognized in Rwanda and successfully used by the Maternal and Child Survival Program (MCSP) in the areas of maternal and child health (MCSP 2018).

Growing evidence suggests benefits of flexible, mobile solutions for continuous education (Car et al. 2019) but these solutions have not yet been implemented at the PHC level. Supervision personnel are often located at district level, far away from the HWs. Due to logistical challenges, supervision visits can only happen infrequently and lack efficacy and efficiency. This is largely because the district supervision teams do not possess data that could inform them about specific problems and needs in the individual health facilities. For instance, studies have found that supervision methods including audit with feedback can be moderately effective at increasing HW competence (Rowe et al. 2018); however, the supervisor and supervisee would greatly benefit from a personalized, data-driven system to guide the process which does not exist to date in low-resource settings. The evidence base for the role of digital tools in HW training and supervision is currently limited.

1.4 Monitoring and evaluation

Currently, the planning, budget allocation, monitoring, and evaluation in the health sector is largely based on reports of data aggregated at the facility level and managed in HMIS platforms such as DHIS2 used by many African countries. Data aggregation at the facility is done manually on paper forms or excel tables using paper records that are supposed to be filled by HF personnel on a daily basis. Since the filling of these registers is additional work on top of patient care, they are often filled retrospectively and hence may be inaccurate and incomplete (Wilms et al. 2014). Furthermore, the manual data aggregation at the facility level and entry process into HMIS is error prone. In sum, the data available for health management and reporting is of insufficient quality (Mghamba et al. 2004, Farnham et al. 2020).

Even if HWs are motivated to report correct data, they lack the tools to make correct diagnoses. As a result, prevalence of many conditions that require diagnostic tools that are often out of stock or not

available altogether, is falsely represented in HMIS data. For example, bacterial pneumonia is constantly over-diagnosed as diagnostic tests to distinguish bacterial from viral respiratory infections are not available. Misdiagnoses have consequences that reach beyond the patient. They increase re-attendance rates, further congesting PHC facilities and accruing economic losses not only for families but for the entire health system. Systematic errors in patient-level data accumulate, and as they are aggregated in HMIS to measure population-level indicators, they have the potential to bias the statistics used to prioritize public health interventions and, importantly, identify epidemics.

1.5 Clinical decision support algorithms

The WHO has identified digital health interventions and predictive tools in primary care as key accelerators in achieving the 2030 Sustainable Development Goal 3 of ensuring good health and well-being for all. New simple and cheap technologies, such as mobile devices, coupled with the advances in computing and data science, can help mitigate several of the challenges described in sections 1.1-1.4.

Thus, Unisanté and Swiss TPH have been developing and clinically validating electronic CDSAs for the management of sick children since 2010. Two versions of the algorithm have been developed and evaluated in controlled research condition to date:

1) The first-generation algorithm for the management of childhood illness (ALMANACH) was trialed in Tanzania in 2010-2011. The content of ALMANACH was largely based on IMCI guidelines. ALMANACH achieved improved clinical cure (from 92% to 97%) and a decrease in antibiotic prescriptions (from 84% to 15%) as compared to routine care (Shao et al. 2015A). It also led to more consistent clinical assessments without taking more time than a conventional consultation and was perceived by clinicians as “a powerful and useful” tool (Shao et al. 2015B).

2) The second-generation algorithm called ePOCT, ‘electronic Point of Care Tests’ was also trialed in Tanzania in 2014-2016. Its medical content was based on a thorough review of the evidence and the existing national and international guidelines on the diagnosis and management of sick children (Keitel et al. 2017). In addition to symptoms and signs, it made use of several POC tests to help detect children with severe infections requiring hospital-based treatment and/or children with serious bacterial infection. It increased the ability to detect children with severe disease through the use of pulse oximetry and POC hemoglobin testing (to screen for severe anemia, a strong predictor of bacteremia). POC inflammatory host biomarkers, such as CRP, detected children in need for antibiotic treatment. Diagnostic and triage tests were only performed in patient groups with sufficient pre-test disease probability (which is indicated by the algorithm). The safety of ePOCT was assessed among over 3,000 febrile children aged 2 months to 5 years through a randomized trial in Dar es Salaam, Tanzania in 2014-2016 (Keitel et al. 2017); this was an essential first step since ePOCT contained important upgrades compared to IMCI. The use of ePOCT resulted in higher clinical cure (98%) as compared to ALMANACH (96%) and routine care (95%). The algorithm also further reduced antibiotic prescription to 11%, as compared to 30% with the use of ALMANACH and 95% in routine care (**Figure 1**).

Figure 1. Summary of gaps that ePOCT filled [left] and its impact [right] illustrated as the percentage of antibiotic prescription for children under-five with fever when managed per e-POCT and ALMANACH algorithms, and per routine care in Dar es salaam, Tanzania.

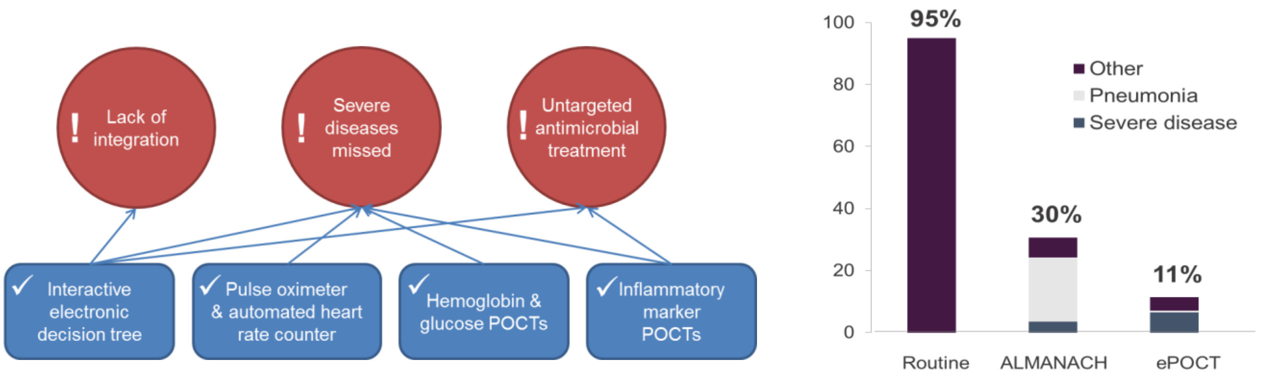

1.6 Proposed intervention

The proposed intervention includes a third-generation algorithm called ePOCT+. Its medical content has been expanded from the prior version (**Table 1**) to cover not only febrile children aged 2 months to 5 years but the entire pediatric age range and clinical spectrum so that the clinicians can use the tool for all their pediatric consultations. Additionally, a young infant (0 to 2 months) module has been added, as this age group is at highest risk of mortality. The implementation of ePOCT+ will be accompanied by other electronic tools to facilitate uptake and integration into the health system and ensure sustainability. The additional features of the intervention include: a) e-learning modules to enable continuing education for HWs; b) an electronic platform that will use patient-level data to enable targeted supportive supervision of HWs and enhance M&E capacity at the local level; and c) an electronic platform that will allow clinicians with no programming skills to conduct data analyses and adapt the clinical algorithm to changing epidemiological conditions.

The goal of the DYNAMIC project is to demonstrate the potential of digital CDSAs like ePOCT+ to improve care through further validation and implementation studies in different settings and in less research-like conditions (**Figure 2**). Implementation research will be conducted throughout the project, evaluating its feasibility, cost-effectiveness and impact, and to understand facilitators and barriers for further scale-up.

Table 1: Evolution of the clinical decision support algorithm from IMCI to ePOCT+

	**IMCI (2014)**	**ALMANACH**	**ePOCT**	**ePOCT+**
Format	Paper-based	Electronic CDSA (static)	Electronic CDSA (static)	Electronic CDSA (dynamic for phase 2)
Age	0-2 months and 2-59 months	2-59 months	2-59 months	0-14 years 11 months
Fever	Febrile and non-febrile	Febrile and non-febrile	Febrile only	Febrile and non-febrile
POCTs	mRDT, HIV rapid test, pulse oximeter (optional)	mRDT, typhoid test*, urine dipstick*	mRDT, CRP*, PCT*, Hb*, HIV, pulse oximeter*	mRDT, HIV, CRP*, Hb*, pulse oximeter*, urinalysis, syphilis rapid test (if available)
Modifications in algorithms compared to IMCI (2014)	Reference	Minor modifications:<br>- danger signs<br>- respiratory rate cut-offs	Minor modifications:<br>- danger signs<br>Major modifications:<br>- pneumonia<br>- dehydration<br>- malnutrition<br>- anemia	Minor modifications:<br>- danger signs<br>- ear infection<br>- measles<br>Major modifications:<br>- pneumonia<br>- dehydration<br>- malnutrition
Additional conditions compared to IMCI (2014)	Reference	- Jaundice<br>- Abscess<br>- Typhoid<br>- Urinary tract infection	- Respiratory illnesses (reactive airway disease)<br>- Skin infections (abscess, cellulitis, chicken pox, etc)<br>- Ear, nose and throat (dental abscess, neck mass)<br>- Other (osteomyelitis/septic arthritis)	- Respiratory illnesses (croup, reactive airway disease, foreign object in airways)<br>- Ear, nose and throat (pharyngitis, dental abscess, oral ulcers, candidiasis, neck mass)<br>- Eye problems (conjunctivitis, orbital cellulitis, etc.)<br>- Urine/genital (urinary tract infection, STIs, oxyuriasis, etc)<br>- Skin infections (abscess, cellulitis, chicken pox, etc)<br>- Neurological (poisoning, headache)<br>- Trauma (fracture, bruns, wounds, head injuries, osteomyelitis, etc)<br>- Fever without source<br>- Prevention/screening (TB)

*Provided by the CDSA
CRP = C-reactive protein, Hb = Hemoglobin, mRDT= Malaria Rapid Diagnostic Test, PCT = Procalcitonin.

Figure 2: The validation cycle for clinical decision support algorithms (adapted from Keitel et al. 2018)

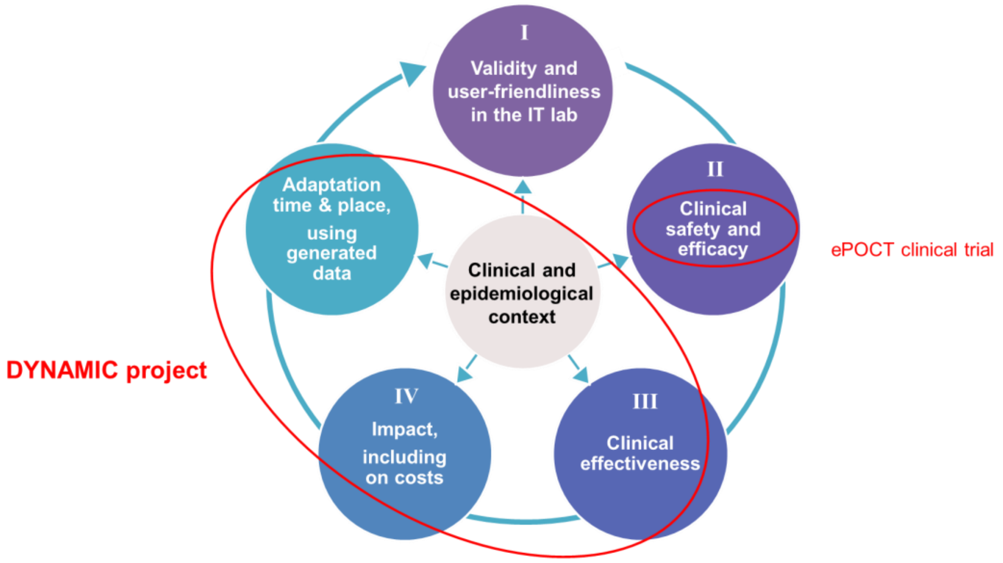

1.7 Anticipated impact

The ongoing digitalization of the health sector bears great potential for improving delivery and quality of care and for using real-time data in public health decision making. The DYNAMIC project aims at integrating innovative cutting-edge technologies and methodologies to improve the quality of care and reduce antibiotic prescriptions in PHC settings. Furthermore, it aims to improve the quality of continuing education, supportive supervision, and mentorship for HWs, while also contributing to more accurate reporting of morbidity episodes through the HMIS and M&E systems built on patient-level data. Lastly, it aims to contribute to the development of national frameworks and guidelines for the scale-up of the intervention, while bearing in mind potential socioeconomic and ecological impacts.

ePOCT+ should better identify children with severe disease who require immediate referral, treatment and supportive care. Early and appropriate triage and management not only leads to reduced mortality and morbidity but also decreases exposure to unnecessary antimicrobials, which protects their natural gut flora which is essential for the development of immunity. Rational use of antimalarials and antibiotics would result in a reduction of the drug pressure in the community and AMR, and eventually a reduction in morbidity, mortality and health care costs due to antimicrobial resistant infections.

ePOCT+ is designed to promote social cohesion between patients and HWs since it presents structured consultation steps which encourage dialogue and physical examination (Bessat et al. 2019). It saves time on trying to establish the right diagnosis, allowing clinicians to focus on social communication and fostering confidence through the perceived efficacy and reduced waiting times. Because ePOCT+ is designed to provide personalized treatment algorithms, the clinician is then made more aware of the individual characteristics of the patient in her/his environment. The project also aims to improve the working relationships between the HWs and their supervisors by facilitating supportive supervision and mentorship activities.

Implementation of digital health interventions also has negative impacts – studying these potential negative impacts and planning for mitigation measures in future scale-up is within the scope of the DYNAMIC project. Rapid expansion of eHealth has a carbon footprint and potential negative ecological effects that must be quantified and alternatives evaluated. These impacts can be reduced if the devices are used for other purposes, such as EMRs and HF management systems that health authorities are in the process of deploying at the PHC level. Important issues of patient confidentiality, data security, fragmentation of work, duplication of efforts, interoperability, and transparency within the health system will also be considered. These features of technological

innovations have significantly increased the need for national governance frameworks within the health sector. The DYNAMIC project intends to generate knowledge to inform these policies, frameworks, and guidelines on eHealth in Rwanda.

2 STUDY OBJECTIVES AND THEORY OF CHANGE

The **goal** of this study is to mitigate AMR using a novel dynamic ePOCT+ tool that helps front-line HWs manage sick patients, enhanced by additional digital platforms to promote uptake, sustainability, and integration of the tool into the health system. More specifically, this study seeks to:

2.1 Objective 1

Improve the integrated management of children with an acute illness (more specific and diverse diagnoses; reduced antibiotic prescriptions) through the provision of an electronic CDSA (ePOCT+) to clinicians working at primary care level.

Hypothesis 1: The use of ePOCT+ in the outpatient PHC setting in Rwanda will result in decreased prescription of antibiotics while maintaining similar clinical outcomes.

Hypothesis 2: The use of ePOCT+ in the outpatient PHC setting in Rwanda will result in improved quality of care.

2.2 Objective 2

Improve the diagnostic and prognostic accuracy of the clinical algorithm across spatiotemporal variations in epidemiology and resources through machine learning analyses of the data generated through the ePOCT+ tool, checked by clinical experts.

Hypothesis 3: Modifications in the electronic CDSA based on the results of ML analyses will enable similar or better outcomes over the course of the study despite temporal and spatial variability in disease patterns and/or availability of resources.

2.3 Objective 3

Enhance the health management information system for monitoring and evaluation and supportive supervision (increased number of meaningful indicators that are reviewed and actioned regularly) through additional data analysis and visualization of patient-level data generated by the ePOCT+ tool.

Hypothesis 4: The patient-level clinical data that is visualized and analyzed in near real-time will lead to improved ability of the DHMT to monitor the quality of patient care and undertake targeted supportive supervision of HFs and HWs.

Hypothesis 5: The patient-level clinical data that is visualized and analyzed in near real-time will lead to a more accurate epidemiological profile of the district and improved spatial and temporal resource allocation.

2.4 Objective 4

Create a framework for the implementation of dynamic clinical decision support algorithms and supporting tools, for large-scale, sustainable, and clinically responsible use of machine learning and data science in digital health.

Hypothesis 6: The interdisciplinary nature of the project, with an emphasis on cross-sectoral collaboration and knowledge/skills transfer, will enable the development of an evidence-based framework for sustainable scale-up of electronic CDSA interventions.

2.5 Theory of change

The goal and the objectives of the project are based on the following theory of change (**Figure 3**):

Figure 3: DYNAMIC project theory of change

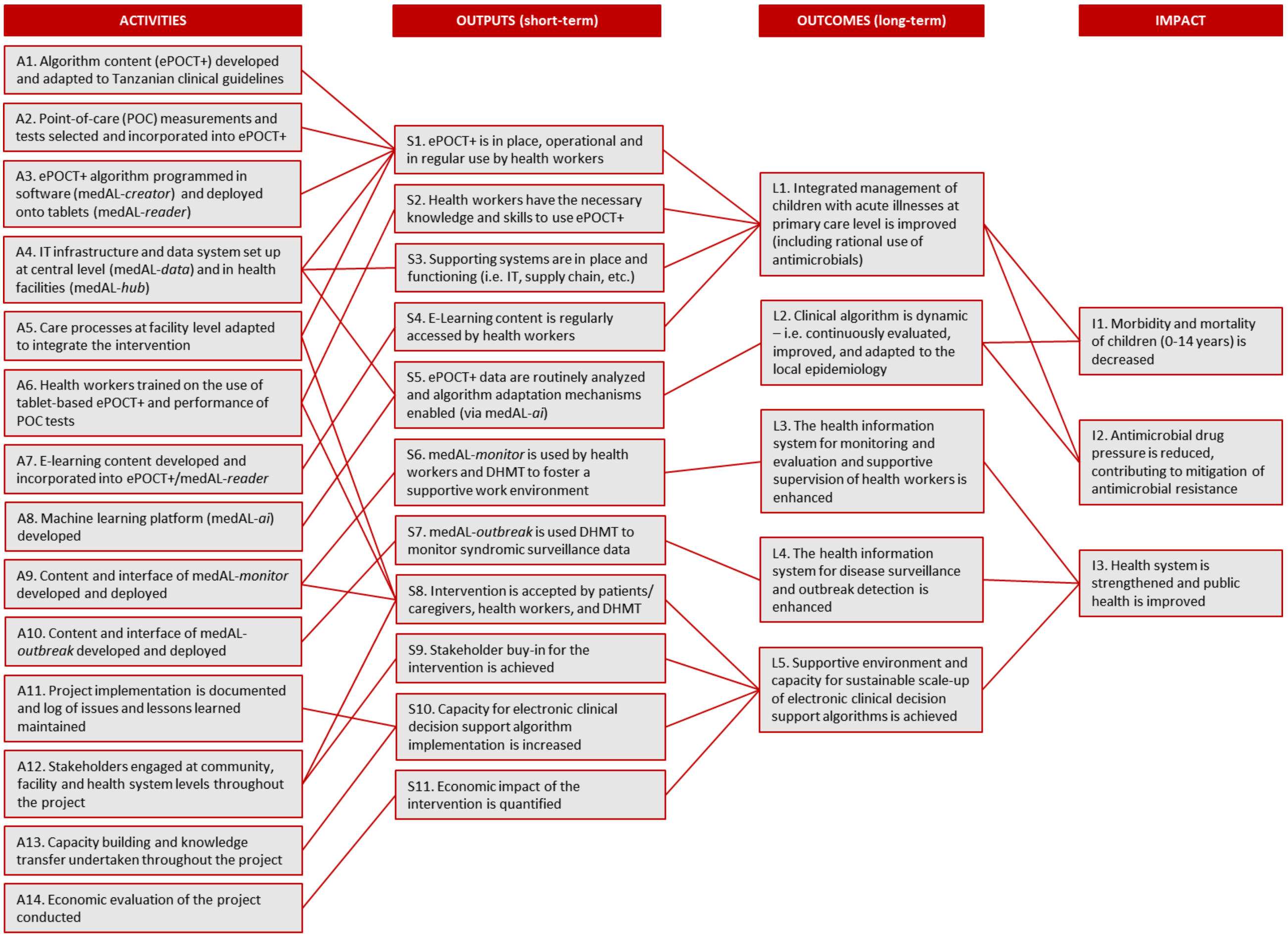

3 DESCRIPTION OF THE INTERVENTION

The intervention will include four activities that are in line with the objectives of the study and supported by specific electronic tools (**Figure 4**).

3.1 Management of sick children by health workers using ePOCT+

Acutely ill children will be assessed, diagnosed and treated by routine clinicians guided by the ePOCT+ application, which will be loaded on a tablet or PC.

The intervention will be undertaken in public PHC facilities in Rwanda and entails the implementation of the ePOCT+ tool for patient assessment and management, with the provision of associated POC tests. Healthcare providers will use ePOCT+ throughout their medical consultations for children aged 0-14 years (inclusive) presenting with an acute medical or surgical problem. In case of a re-attendance visit for the same illness episode, ePOCT+ will be used for managing the child in the same way as during the initial visit (taking into account the history of illness and prior test results and medications prescribed). Equipment, reagents, supplies/consumables for all laboratory and diagnostic tests

included in the algorithm that are not currently part of the standard of care in the study setting, will be made available to providers throughout the duration of the study.

Figure 4: Electronic tools on tablets or PCs used in the DYNAMIC project to improve the clinical management of sick children and enhance M&E and supervision. Icons were obtained from open sources: https://healthicons.org/ and https://openclipart.org/.

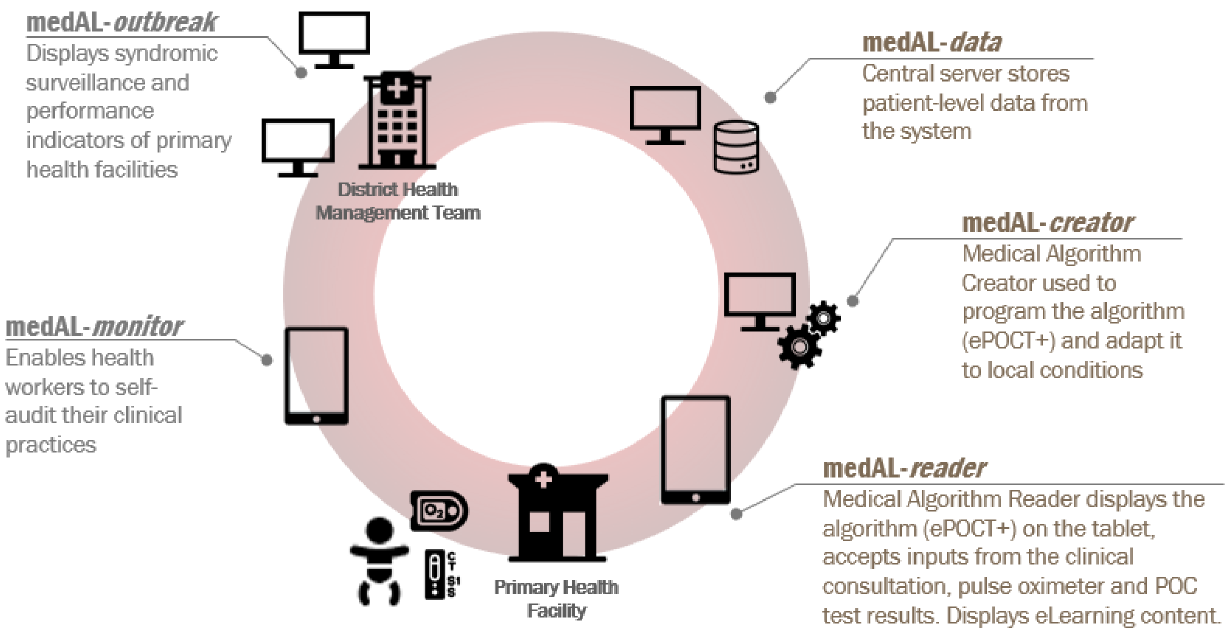

The current CDSA of ePOCT+ is based on international guidelines and protocols (WHO Pocket Book of Hospital Care for Children 2013; WHO IMAI guidelines 2009, ETAT guidelines 2016, etc.) and is being updated based on the Rwandan national guidelines for pediatric clinical management (e.g. IMCI Rwanda booklet 2020; Pediatric protocols 2014, Neonatal protocol 2019, Pediatric emergencies 2012, National Guidelines for Prevention and Management of HIV and STIs, Malaria clinical algorithm 2019, Protocol for management of acute malnutrition 2012, Guidelines for NTDs and other parasitic diseases, Treatment guidelines, etc.). The medical algorithm will be reviewed by key national experts in child health (Rwandan Pediatric Association; Ministry of Health; Rwanda Biomedical Centre) through a Delphi validation process. The final version of the algorithm will be presented to the MoH for final approval before implementation in the HFs.

The exact medical content of the ePOCT+ algorithm will be published online, on a website that will be dedicated to the DYNAMIC project. Any subsequent modifications made to the original algorithm will be approved by the Rwandan clinical expert group before being published and implemented in HFs.

Specifically, the ePOCT+ tool will guide clinicians on:

i) Decision on immediate referral to a higher level facility
ii) Medical history (questions to ask caregivers and/or children)
iii) Physical exam (signs to look for)
iv) POC diagnostic tests that should be performed in a given clinical context (**Table 2**)
v) Diagnoses
vi) Treatments (including medicines and supportive treatments)
vii) Counseling (explanation of final diagnoses, treatments, and follow-up)

Biosensors (presently pulse oximeter and hemoglobinometer) and rapid tests (presently malaria, HIV, semi-quantitative CRP, and urine dipstick) used for the intervention will have to meet requirements for use at the PHC level, including adequate measurement accuracy, stability in high-temperature and dusty settings, and affordability. Once adequate biosensors and POC tests are identified, manufacturer negotiations are complete, devices are procured, and the regulatory framework is in place, they will be used by HWs upon recommendation provided by ePOCT+ (**Table 2**).

The ePOCT+ CDSA guides the clinician step-by-step through the consultation process. The algorithm is able to integrate all the information from the consultation and provide diagnostic classifications and related treatment recommendations. The application will be radically different from the existing software used in the clinical settings such as eIMCI. It will allow clinicians to freely navigate up and down the algorithm, adding or modifying clinical information of any type (exposures, symptoms, signs, lab test results) at any time, in order to follow the natural consultation process (rather than forcing a clinician to assess the patient condition in a fixed order).The interface will also be much more user-friendly, and allow integration of alerts of ongoing epidemics, definitions or translations of medical terms in local languages, explanations and illustrations (pictures, sound or video clips) of symptoms or signs that are difficult to assess and those that are specific to rare diseases, and educational information on why a certain disease is being considered or not considered in a particular patient. The application will be available in English and French, with the possibility of also translating the client-facing content into Kinyarwanda.

In addition to the patient management algorithm, the overall ePOCT+ ecosystem contains a patient registration module and a vitals/triage module that can be used by different providers within the same HF, using tablets that are connected via the internet or an internal network. The purpose of the triage module is to prioritize patients that most urgently need medical attention and to allow task-shifting and optimization of the care pathway. The ePOCT+ triage module is based on the WHO ETAT guidelines, which have been implemented in Rwandan district hospitals (Hategeka et al. 2017) but not in the PHC setting, and the IMCI-based SCREEN tool. The triage module can be switched on and off within the application to allow flexibility to the facility staff to decide on how best to organize their workflows and task-shift (e.g. by having a lower level staff member recording all vital signs or performing the necessary rapid tests) so that the clinician can gain time during the consultation for tasks that cannot be delegated. Based on previous experience, the average consultation time while using ePOCT+ is expected to be 15 minutes.

ePOCT+ can be loaded onto a tablet or computer, depending on the IT platform already in use in the HFs and the level of comfort of providers with various electronic devices. We will perform pilot testing of the ePOCT+ tool in select HFs with direct supervision from qualified medical personnel before the start of study recruitment. The aims of this pilot phase are to ensure that ePOCT+ content is understandable and acceptable to the users, its flow conforms to the consultation process, and the interface is optimized to ensure positive user experience.

Table 2: Point-of-care tests included in the ePOCT+ CDSA

POCT	Targeted patient group	Rationale
Pulse oximetry (POX)*	Patients with respiratory complaints and severe disease; young infants with suspected severe disease	POX may improve detection of children with hypoxemia<br>In neonates and young infants, among whom accurate respiratory rate can be difficult to ascertain and short instances of rapid breathing or apnea can be physiologic, POX may improve diagnosis and classification of severe illness
Hemoglobin (Hb)*	Patients ≤ 5 years with fever; patients 6-14 years with malnutrition, palmar pallor or conjunctival pallor	Regular Hb measurements in febrile children will improve detection of severe anemia, which is a marker of severe disease for a variety of disease pathways, including severe malaria, sickle cell disease, and severe bacterial infections
Malaria rapid diagnostic test (mRDT) or microscopy	Patients with fever	Malaria is a potentially fatal disease that must be excluded in patients presenting with fever, or in a sub-group of febrile patients at high risk in very low endemic areas, as per current WHO guidelines
C-reactive protein (CRP)	Febrile patients at risk for bacterial pneumonia (based on clinical and demographic features); febrile patients without focal clinical signs; febrile patients with joint or extremity pain; infants aged 1-2 months with suspected pneumonia or bacterial infections	CRP improves targeting of antibiotic prescription in children with respiratory symptoms, with fever without focal signs, and with joint/extremity pain at risk of septic arthritis or osteomyelitis<br>CRP may improve specificity of diagnoses among young infants with non-specific signs of possible severe illness and inform targeted antibiotic treatment and appropriate supportive care
HIV rapid test	Patients with possible exposure to HIV (mother with HIV or mother with unknown HIV status and unavailable for testing, or the patient is sexually active); clinical suspicion of HIV; informed consent from the caregiver must be acquired	Rwandan national HIV program no longer recommends provider-initiated HIV testing but only upon clinical suspicion of HIV
Urine dipstick / urinalysis	Febrile patients <3 years without focal clinical signs; febrile patients > 3 years with complaints suggestive of urinary tract infection	CRP does not reliably rise in children with febrile urinary tract infections; febrile children <2 years should be assessed for febrile urinary tract infections per national guidelines<br>In older patients, for whom urine collection is easier, a urinary tract infection can be excluded using a urine dipstick

**POX, CRP and Hb cuvettes will be provided by the DYNAMIC project, all other tests are routinely available at PHC level.*

3.2 Modification of ePOCT+ to improve accuracy and adapt to epidemiological changes

The ePOCT+ algorithm will be programmed in an electronic software support system that allows direct visualization and internal validation of the algorithm and subsequently deployed onto the users' devices. For this, a specific software solution is currently being developed which has three main components (**Figure 5**):

- The clinical algorithm management system (called medAL-creator) that will allow a clinician without any IT programming skills to manage, reference, modify and deploy different versions of the algorithm into a mobile device;
- The tablet- or PC-based application (called ePOCT+) that will contain the algorithm and be used for data acquisition by the HW (called medAL-reader), as well as automated capture of results from biosensors and rapid tests;
- The data management system that will receive and manage the data on a central server with a database.

Figure 5: General architecture of the software solution. Icons were obtained from open sources: https://healthicons.org/ and https://openclipart.org/.

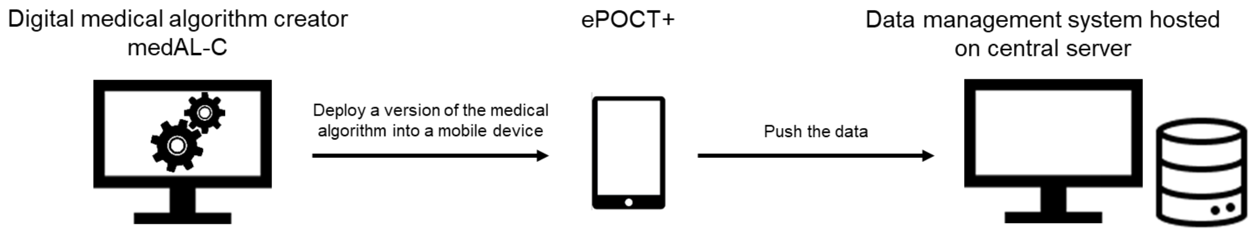

In the second phase of the study, the algorithm will be modified according to trends in the collected data detected using ML or conventional statistical methods. The modifications will be specified and validated by clinical experts. Approved changes will then be integrated into the algorithm using medAL-creator and a new version of the ePOCT+ algorithm released. The new version will then be deployed onto the tablets or PCs of the relevant HWs (for example of a certain region where an epidemic is occurring) with a message explaining the rationale and evidence behind the change in the algorithm.

Modifications in the algorithms designed to perform as well as the original algorithm but using fewer/different resources will be implemented in the facilities they were designed to serve (for example, if certain facilities do not have access to a certain resource). Similarly, modifications designed to personalize the algorithm according to demographic characteristics, geography or season will be implemented on the target population for which they were intended.

3.3 Enhanced clinical competencies of health workers

Supportive supervision

To enable better supervision of HWs by the district medical team, we will also develop and deploy an electronic supervision tool called medAL-monitor. We will define clinical performance indicators, based on the data collected through ePOCT+, and integrate them into the electronic supervision tool. The tool will contain dashboards to visualize these indicators and monitor their spatial and temporal trends. The dashboards will be adapted to different users who would benefit from different indicators

displayed to them in different ways. For example, at the district level, a dashboard will be enabled that will allow the DHMT to assess differential performance across health facilities and prioritize under-performing facilities for supportive supervision visits based on a set of criteria and indicator cut-off values. At the level of the HF in-charge or individual HWs, more granular information may be desired, aggregated at the level of provider rather than facility. Here, the user may wish to view trends in their own performance on a daily, rather than monthly basis. medAL-monitor is intended to enable and encourage more frequent two-way communication between remote HWs and the DHMT team. The supervision tool will be pilot-tested with the DHMT and HWs before routine implementation.

e-Learning

To continue improving providers' clinical skills throughout the project, e-learning modules will be developed and incorporated into ePOCT+. Such modules will help clinicians assess important clinical signs, especially pediatric danger signs (e.g. chest indrawing). First, we will perform an assessment of additional training needs among HWs. Next, we will screen available written materials, photos, sound clips, and video tutorials for suitability and adapt and translate them into the relevant languages for the Rwandan context, as needed. We will also develop additional training modules on clinical topics for which no training materials are available. The e-learning modules will then be pilot-tested among HWs and then deployed onto the clinicians' tablets and integrated with the ePOCT+ workflows.

The e-learning platform will be revised and upgraded throughout the project. Initially, a set of "information" buttons will be available for certain syndromes or clinical procedures so that the HW will be able to consult them in real-time. All of this content will also be curated in a reference library, which HWs will be able to consult at any time (e.g. in the afternoon after seeing patients). In the latter stages of the project, the e-learning process will be made more interactive, enabling the learner to answer questions and progress through the modules, reaching certain milestones. The approach will be further refined during the pilot stage, taking into consideration available platforms and certification processes for clinical skills required for IMCI that are currently available in Rwanda and in similar settings.

3.4 Enhanced monitoring and evaluation

Beyond facilitating supportive supervision, the medAL-monitor/medAL-outbreak platform will also be used to enhance the M&E capacity in the intervention districts. Dashboards created with ePOCT+ data will enable the DHMT to monitor certain health indicators over time, be alerted if indicator values are higher than expected for a given month in a given location, suggesting a potential outbreak (or another change in the health system), and be able to decide if a response is needed.

As previous mentioned, different versions of the dashboard will be developed: for the HFs, the district level and the national level. Furthermore, the dashboards will have several pages for different types of health statistics, including but not limited to general information, main symptoms or syndromes, acute diseases, chronic conditions, and clinical management including treatment and referral. Specific indicators for self-audits (HF level), supervision (district level), M&E (district and national level) and syndromic disease surveillance (district and national level), will be available. Examples of important M&E indicators related to the impact of the present intervention that can all be stratified by geographical area, time period and age group are shown in **Table 3**.

It is important to note that medAL-monitor/medAL-outbreak at this stage is a concept rather than a specific IT platform. In fact, at the district level, the preliminary list of M&E indicators is intentionally similar to that available in the Rwandan HMIS. However, with the use of ePOCT+ in the intervention HFs, de-identified non-aggregated patient-level data serve as a data source for medAL-monitor/medAL-outbreak, rather than aggregated values based on fixed categories in the HMIS. This allows much more flexibility in how these data can be aggregated and displayed based on the various users' needs. All functionalities of HMIS (e.g. DHIS2) and other platforms in which indicator monitoring

and alerts are enabled will be fully evaluated to ensure that the platform has useful and complementary, rather than redundant features. In the beginning of the project, all indicators consistent with HMIS in terms of their definitions will be transferred into HMIS to ensure continuity of national reporting in intervention facilities but also to serve as a basis for planning for future interoperability. The MoH will thus be able to compare the data provided by the HMIS dashboard with that provided by the Clinivisor dashboard, for the 2 districts where ePOCT+ will have been implemented. If and when the MoH is ready to do so, interoperability between the two systems can be pursued.

Table 3: Examples of M&E indicators that will be possible to display in the medAL-monitor/medAL-outbreak dashboards based on ePOCT+ data

**Consultations**	
	• Number of outpatient consultations
**Main indicators**	
	• Number and % of deaths<br>• Number and % of children referred to hospital<br>• Number and % of children with malaria (severe and non-severe)<br>• Number and % of children with bacterial pneumonia (severe and non-severe)<br>• Number and % of children with diarrhea (severe and non-severe)<br>• Number of measles cases<br>• Number and % of children prescribed an antibiotic (by level of disease severity)<br>• Number and % of children prescribed an antimalarial (by mRDT status)<br>• % of final diagnoses made by HWs for which the appropriate treatment is prescribed
**Danger signs**	
	• % of children with at least one danger sign<br>• % of children with each type of danger sign<br>• % of children with at least one danger sign prescribed an antibiotic<br>• % of children with at least one danger sign prescribed an antimalarial
**Among children with cough/difficulty breathing**	
	• Number and % of children with cough/common cold, viral pneumonia and bacterial pneumonia<br>• Median and distribution of respiratory rate measurements<br>• % of fast breathing among children with cough/difficult breathing<br>• % of chest indrawing among children with cough/difficult breathing<br>• Median and distribution of oxygen saturation measurements<br>• % of hypoxemia among children with fast breathing or chest indrawing<br>• % of children with bacterial pneumonia prescribed an antibiotic<br>• % of children with viral pneumonia prescribed an antibiotic<br>• % of children with cough/common cold prescribed an antibiotic<br>• % of children with bacterial pneumonia prescribed the first line antibiotic
**Among children with diarrhea**	
	• % of children with diarrhea<br>• % of dehydration among children with diarrhea<br>• % of dysentery/persistent diarrhea among children with diarrhea<br>• % of children with diarrhea prescribed ORS/zinc<br>• % of children with diarrhea prescribed an antibiotic
**Among children with fever/elevated temperature**	
	• % of children with confirmed versus clinical malaria<br>• % of children with positive mRDT result<br>• % of mRDT-positive children prescribed an antimalarial<br>• % of mRDT-negative malaria children prescribed an antimalarial<br>• % of children with malaria prescribed the first line antimalarial
**Screening for chronic conditions**	
	• % of sick children tested for anemia<br>• % of sick children assessed for malnutrition<br>• % of sick children tested for HIV

	• % of children with anemia (severe and non-severe)<br>• % of children with malnutrition (severe and moderate)<br>• % of HIV-positive children

4 STUDY DESIGN

The primary intervention (i.e. ePOCT+) will be implemented using a parallel-group cluster non-randomized controlled superiority trial study design in two phases. In Phase 1 of the implementation, the medical content of the algorithm will be static. In Phase 2, the medical content of the algorithm will be modifiable according to the local data captured by ePOCT+ to date. Throughout Phases 1 and 2, mixed-methods operational research will be conducted to understand change in the quality of care, the implementation context, user and patient experience, and facilitators and barriers to the intervention at the individual, health facility and district levels.

4.1 Primary intervention

As part of the primary intervention in Phase 1, the ePOCT+ tool, as well as the complementary e-learning and supervision tools, will be provided to HWs of 42 PHC facilities in Rusizi and Nyamasheke districts of the Western Province in Rwanda for the management of sick children aged 1 day to 14 years (inclusive). The intervention will be implemented in blocks of 10-12 (half intervention and half control) HFs (**Figure 6**). Each step will last for four months, with the first month serving as a phase-in period. To assess the primary and secondary outcomes in Phase 1 (i.e. antibiotic prescription and cure rate), final decision on referral, diagnoses and treatments at initial consultation (day 0) will be recorded by HWs electronically in intervention and control facilities. All children in Phase 1 of the study will also be followed-up by a phone call or SMS (or home visit) to their caregiver at day 7 to determine their final health status. The CRFs are presented in **Appendix A**.

Due to the pragmatic nature of the study, the design is adaptive, in that changes to the implementation may be incorporated during each of the three parallel cluster studies to account for issues encountered in monitoring data and feedback from the field. These changes apply to the implementation context (such as training, frequency or focus of monitoring visits, etc.) and exclude significant adaptations of the algorithm.

The primary outcome (antibiotic prescription rate) will be compared between intervention and control facilities within implementation blocks (e.g. group 1 will be compared with group 2, group 3 with group 4, etc.). To assess the secondary outcome (cure rate), the data from all intervention periods (groups 1, 3 and 5) and control periods (groups 2, 4 and 6) will be combined to achieve a sufficient sample size. Additionally, HFs within the same group will also be compared longitudinally (e.g. outcomes during control and intervention periods can be compared in groups 2 and 4).

In Phase 2, the same outcome indicators as in Phase 1 will be measured and evaluated over time to monitor longitudinal changes in the effectiveness of the intervention. The medical content of the algorithm will not be fixed anymore, but rather modifiable. Each potential modification will first be evaluated by the Rwandan clinical expert group for its clinical coherence, safety and potential benefit and then applied to the retrospective data. If retrospective analyses confirm a clinically relevant positive impact (or resource saving), the modification will be implemented in the relevant locations and/or patient sub-groups.

Health facilities will need to demonstrate successfully meeting the objectives of Phase 1 before entering Phase 2. In case of any deviations from expected outcomes, or any issues that preclude reaching the study objectives as part of the parallel cluster study in Phase 1, design and implementation will be re-evaluated. In this case, Phase 1 would continue in modified form instead of moving on to Phase 2. Regardless of study phase, outcomes will be continuously monitored in all facilities, and if outcomes are not trending as expected, the causes will be investigated as part of routine monitoring of study implementation by the research team in collaboration with the DHMT.

Figure 6: Study design overview

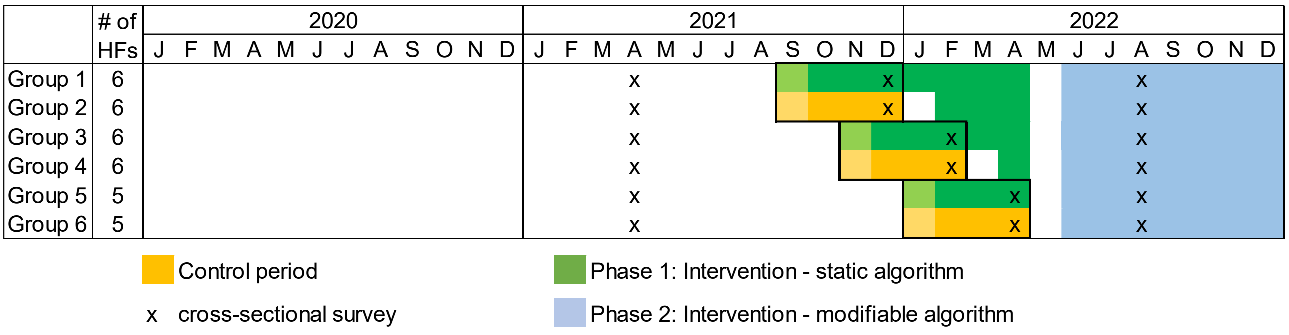

4.2 Mixed-methods operational research

Throughout the project, mixed-methods operational research studies will be conducted in HFs and communities, as well as at district and national levels, to understand the contextual factors that facilitate or impede the implementation, uptake, or sustainability of digital health interventions such as ePOCT+ in PHC settings. Examples of such factors may include socioeconomic and ecological profiles of patients and communities, working conditions of the clinicians in the HFs, general management of HFs, among others.

4.2.1 Baseline health facility assessment

Prior to the intervention, a baseline HF assessment will be conducted in all facilities chosen for the study to assess their basic readiness to provide health services, with an emphasis on child health. The questionnaire used in this survey is an abbreviated version of the Service Provision Assessment (SPA) inventory tool used nationally, complemented with additional questions that relate specifically to the implementation of digital health interventions. The survey will be repeated annually in all HFs regardless of where they are in the implementation process to ensure that no major changes in the general operation of the facilities have occurred that could bias the results of the study. The modified tool used in the study can be found in **Appendix B** (B1).

4.2.2 Cross-sectional quality of care survey

Cross-sectional surveys will be conducted to assess changes in the quality of care over time, from the baseline through the implementation of ePOCT+. Two main rounds of the survey will be conducted spaced approximately 20 months apart: prior to the intervention and during Phase 2. To account for the sequential start of the three parallel cluster studies, three additional smaller rounds of the survey will be conducted to constitute a third full round of the survey and to systematically compare quality of care between intervention and control facilities. Timing of the cross-sectional survey is marked by "x" in **Figure**. The surveys will be conducted in a subset (n=18) of randomly selected HFs to assess the quality of the consultation done by HWs and their level of compliance to the algorithm (and thus to guidelines). Clinical research assistants will attend and observe (without interfering) 25 consecutive consultations with children enrolled into the study per HF, thus a total of 450 HW/child pairs per round. Concurrently, client exit surveys will be conducted at the end of the consultation (linked to the observation data) to evaluate the caregiver's perception of the health care the child received. The questionnaire for this survey is adapted from the Service Provision Assessment (SPA) sick child client exit interviews (USAID et al. The Demographic and Health Surveys Program), a methodology recommended by the WHO. The modified tools used in the study can be found in **Appendix B** (B2, B3).

4.2.3 In-depth interviews, focus group discussions and surveys with health workers

Qualitative semi-structured interviews and focus group discussions will be used to assess several aspects of the HWs' experience with the intervention, such as: 1) experience using the ePOCT+ tool; 2) management of tablets and the associated IT infrastructure in the facility; 3) training on the content and use of the algorithm; and 4) experience with the e-learning modules and the medAL-monitor/medAL-outbreak tool.

For the interviews, HWs will be selected through a mix of purposive and random sampling. Based on the data collected through the medAL-monitor/medAL-outbreak tool, HFs will be selected based on relevant criteria such as levels of uptake of and compliance with the algorithm. Within the HFs, providers will be randomly selected among those available on that day. For focus group discussions, several HFs may be combined in order to obtain the desired number of 6-8 participants. Additionally, quantitative versions of the questions will be asked periodically to triangulate the qualitative findings. The interview and focus group guides, as well as quantitative surveys, can be found in **Appendix B** (B4, B5, B6).

For open-ended qualitative questions, probes will be used to generate further explanations from study participants. Each interview will be conducted as an informal discussion, leaving room for the HW to express additional elements. In order to reduce study bias, participants will be strongly encouraged to be as open as possible in expressing their opinion and reassured on full confidentiality of their responses.

To more specifically evaluate the effect of the intervention on HWs' clinical knowledge and competencies, we may use pre- and post-tests during the trainings and throughout the implementation. The tools for these assessments will be developed at a later stage in the project and are not included with this submission.

4.2.4 In-depth interviews, focus-group discussions and surveys with caregivers

Structured interviews will be conducted with caregivers of sick children (as well as potentially older children aged 9-14 years) who attended HFs that are part of the study. Face-to-face interviews will be conducted using a semi-structured questionnaire. Themes that will be explored include: 1) quality of care received; 2) influence of the tablet on the interaction between clinician and patient/caregiver; 3) confidence in treatment and management using ePOCT+; and 4) impact of the use of ePOCT+ on health seeking behavior. A purposive sampling method will be used to select a wide range of children treated for different pathologies at participating HFs. Quantitative versions of the questions will be asked periodically to triangulate the qualitative findings. Additionally, caregivers may be asked to participate in FGDs. The interview and focus group guides, as well as quantitative surveys, can be found in **Appendix B**. (B7, B8, B9)

4.2.5 Focus group discussions with selected community groups

Focus group discussions will be organized with members of the community regardless of their experience with the use of ePOCT+ for clinical consultation. This is meant to gather unbiased opinions, perceptions, and attitudes of community members toward the use of ePOCT+ and digital health tools in general. The tools for the interviews and focus groups can be found in **Appendix B** (B8).

4.2.6 In-depth interviews with actors within the health system

Development and implementation of the medAL-monitor/medAL-outbreak tool will be done in close collaboration with the district team and the HMIS stakeholders within the Rwanda MoH. Once implemented, members of the DHMT will be interviewed about the impact of medAL-monitor/medAL-outbreak on their ability to supervise and mentor HF staff. For example, they may be asked if the respective tool is accessible with internet connectivity available to them, if the indicators displayed in the dashboards are relevant and appropriate, and if the information displayed is clear, easily interpretable, and actionable. The interviews may be conducted several times for the purpose of

continuous improvement of the tools and for final evaluation of their effectiveness. The tools for the interviews can be found in **Appendix B** (B10).

4.2.7 Economic and ecological impact evaluations

Ecological and human impact

An ecological and human impact evaluation of the project will be assessed in collaboration with the "Sustainability Center" and the "Institute of Earth Surface Dynamics" of the University of Lausanne, to ensure that the intervention has limited impact on (or is even beneficial to) the environment. Indeed, the CO_2 emissions and pollution due to the manufacturing and use of tablets may be lower than that of manufacturing and international and national transport of (unnecessary) antibiotics and HF re-attendance visits or hospitalizations. Life Cycle Analysis (LCA) is a systematic study of the environmental impacts of a product system, which analyzes energy and material flows during its whole life cycle: extraction and acquisition of raw materials, its production, use, and end-of-life treatment/ final waste disposal (Frischknecht et al. 2007). Through such a systematic perspective, the displacement of potential environmental loads between the different stages of the life cycle or between particular processes can be identified and avoided. We will use the LCA methodology, published by the International Organization for Standardization, which gives a framework for LCA and guarantees the transparency of the analysis (see ISO 14040; 2006A and 2006B), to evaluate the ecological and human impacts of the intervention. An ecological assessment of the full intervention will be performed to calculate the carbon footprint or carbon-saving of the intervention.

Cost analysis

Cost analysis will evaluate if care provision using a ePOCT+ in the PHC setting is less costly than under routine conditions. Costs will include costs to patients, providers and the health system by assigning a cost to each procedure performed during consultations, as well as to transportation to the HF or to the hospitalization of the child (Yukich et al. 2010). Costs of resource inputs for providers will be determined based on the current pharmaceutical and supply price list from the Rwanda Food and Drug Administration and interviews with the pharmacy team in charge of procurement and pricing of health commodities at the MoH and RBC. Patient costs will be valued according to self-reported expenditures and lost income due to the travel to and time spent in the HF. Costs of project implementation will include tablets and the associated IT infrastructure, initial and refresher trainings and supervision visits, additional laboratory tests, but will exclude specific research costs.

5 STUDY AREA

5.1 Study sites

This study will be conducted in the Western Province of Rwanda that borders Lake Kivu along its western edge (**Figure 7**). It has a mixture of climates, ranging from Kivu-sea climate along the lake shores, humid mountain climate in the south, dry mountain climate in the north, and temperate climate of the central highlands in the east (Henninger 2012). Average daytime temperature is around 25 °C and relative humidity is generally above 80%. The Western Province receives the highest amounts of precipitation in Rwanda, with rains throughout the year and a short dry period lasting from May to September. The majority of the population in the Western Province live in rural areas (88%) and engage in farming as their predominant source of income (73.9%). The Western Province also has some of the highest rates of extreme poverty (21.6%) in Rwanda (**Table 4**).

In terms of health, Rwanda overall has a relatively low HIV prevalence (among adults), malaria prevalence (among children), and <5 mortality rate, as compared to that of other countries in the region. Malaria prevalence in the Western Province is especially low at 0.5%. Prevalence of fever among children and treatment seeking behaviors are similar to those of other provinces in Rwanda. According to the most recent DHS (2014-2015), among <5 children who experienced fever within the

last two weeks, 45.1% sought treatment in a health facility and 35.8% subsequently took antibiotics (**Table 4**).

The Western Province has seven districts (**Table 5**) which are further subdivided into sub-districts, cells, and villages. Most of the districts are primarily rural with similar population densities, with the exception of Rubavu, which is more urban and has a higher population density. Electricity coverage varies quite widely across the districts from 6-7% in Rutsiro and Nyabihu and >30% in Rubavu and Rusizi. Nyamasheke has significantly lower rates of reported childhood mortality than the rest of the districts according to the 2014-2015 DHS. Two districts in the southern part of the Western Province (Rusizi and Nyamasheke) were chosen for the study due to their higher rates of fever and respiratory conditions according to the 2019 HMIS data. Additionally, Rusizi district has higher malaria prevalence than the other districts and both districts have reasonably high electricity and mobile phone coverage, which is important in facilitating our ability to collect the child outcome at day 7.

Table 4: Demographic and health characteristics of five provinces in Rwanda.

Province	Kigali City	Eastern	Northern	Southern	Western
Population[1]	1’132’686	2’595’703	1’726’370	2’589’975	2’471’239
Population density (per sq km)[1]	1552	274	527	434	420
Rural population (%)[1]	24	93	91	91	88
Total poverty (%)[2]	13.9	37.4	42.3	41.4	47.1
Extreme poverty (%)[2]	4.2	15.3	17.4	16.9	21.6
Health insurance coverage (%)[2]	76.8	72.5	83.8	68.9	72.2
Mean time (minutes) to healthcare	31.4	55.1	43.1	56.2	53.6
Adult (15-49 years) HIV prevalence (%)[3]	6.3	2.4	2.3	2.6	2.4
<5 mortality rate (per 1,000 live births)[3]	42	86	60	66	62
<5 malaria prevalence (% of positive blood slides)[3]	0.0	3.9	0.0	4.4	0.5
<5 children with fever in the last 2 weeks (%)[3]	16.4	21.5	14.2	21.2	17.0
Sought treatment from community health worker (%)[3]	4.1	15.6	7.5	13.9	13.2
Sought treatment from health facility (%)[3]	59.8	54.8	48.4	41.7	45.1
Took antimalarials (%)[3]	6.6	19.2	0.9	12.6	6.0
Took antibiotics (%)[3]	57.5	40	43.6	30.7	35.8

Data sources: 1 – Population Census (2012); 2 – Integrated Household Living Conditions Survey (2016-2017);

3 – Demographic and Health Survey (2014-2015).

Figure 7: Partial map of Africa (A), Rwanda (B) and the Western Province (C) with the study districts highlighted. Figure was drawn in ArcGIS software by A. Kulinkina using the following data sources: country boundaries [Natural Earth]; lakes and waterbodies [RCMRD] regional and district boundaries [Humanitarian Data Exchange].

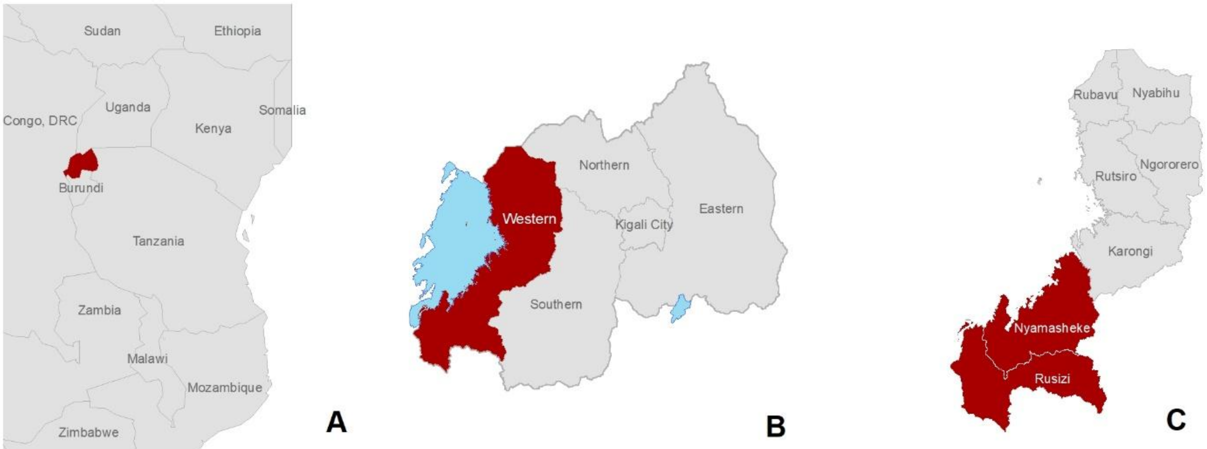

Table 5: Demographic and health characteristics of districts in the Western Province.

Province	Karongi	Ngororero	Nyabihu	Nyamasheke	Rubavu	Rusizi	Rutsiro
Population[1]	331'808	333'713	294'740	381'804	403'662	400'858	324'654
Approximate <5 population	48'592	48'871	43'163	55'914	59'115	58'704	47'544
Approximate <15 population	136'563	137'347	121'307	157'140	166'136	164'982	133'618
Population density (per sq km)[1]	334	491	555	325	1'039	418	281
Rural population (%)[1]	93	96	86	98	63	84	98
Households connected to electricity (%)[2]	17	16	7	20	31	34	6
Households with a mobile phone (%)[2]	64	56	45	56	60	69	48
Household members with health insurance (%)[2]	67	82	78	65	54	72	69
<5 mortality rate (per 1,000 live births)[2]	65	78	62	17	77	61	79
Infant mortality rate (per 1,000 live births)[2]	43	56	35	12	53	41	49
Neonatal mortality rate (per 1,000 live births)[2]	32	38	20	6	23	31	23
<5 children with ARI in the last 2 weeks (%)[2]	8	2	0	5	2	14	5
<5 children with fever in the last 2 weeks (%)[2]	29	9	3	14	11	33	18
<5 children with diarrhea in the last 2 weeks (%)[2]	23	8	6	8	16	24	17
<5 children with anemia - Hb <11 g/dL (%)[2]	37	41	33	41	30	38	18
<5 children who are stunted (%)[2]	49	56	59	34	46	35	46
<5 children who are underweight (%)[2]	8	19	6	6	12	9	12
<5 children who are wasted (%)[2]	0	4	4	1	2	3	3
<5 malaria prevalence (% of positive blood smear tests)[2]	0	1	1	0	0	1.8	0
Cough/difficulty breathing (episodes per 1000 children)[3]	381	275	542	615	475	612	461
Diarrhea (episodes per 1000 children)[3]	128	82	163	128	147	126	157
Fever (episodes per 1000 children)[3]	260	171	242	497	166	408	265

Data sources: 1 – Population Census (2012); 2 – Demographic and Health survey (2014-2015); 3 – National HMIS data

5.2 Selection of health facilities

PHC facilities are defined as the first point of contact to seek curative services. A full list of 291 health facilities in the Western Province was downloaded from the Rwanda MoH database[1]. Concurrently, monthly IMCI indicators from the HMIS were obtained for all primary health facilities. The two datasets were combined to produce a list of 184 public primary health facilities that could be geo-located and had IMCI data available. Of these, 126 facilities met the inclusion criteria (**Figure 8** and **Table 6**). The spatial distribution of the facilities and the corresponding IMCI utilization are shown in **Figure 9**.

Figure 8: Summary of how the exclusion criteria were applies to derive the list of eligible facilities in the Western Province.

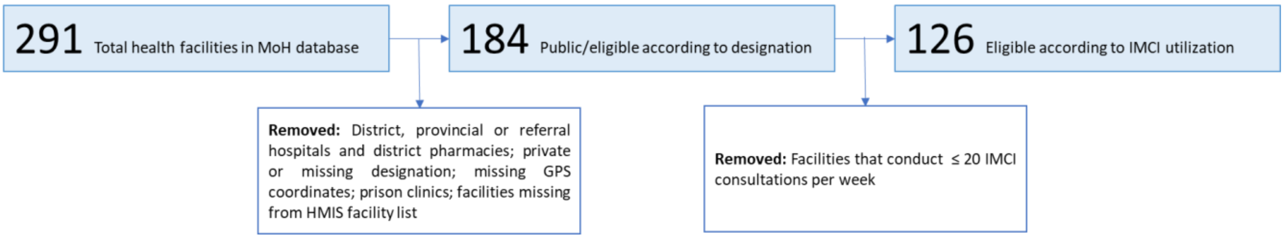

Table 6: Summary of eligible according to IMCI utilization (143) vs. all mapped public/eligible according to designation (195) in the Western Province.

	Health Centers	*Health Posts*	*Community facilities*
Rubavu	12 of 12	2 of 7	--
Nyabihu	13 of 16	4 of 10	--
Rutsiro	16 of 17	2 of 10	--
Ngororero	14 of 15	0 of 1	3 of 9
Karongi	19 of 23	1 of 3	--
Nyamasheke	20 of 20	13 of 22	--
Rusizi	18 of 18	6 of 12	--
**TOTAL**	**112 of 121**	**28 of 65**	**3 of 9**

<u>Inclusion Criteria:</u>

- Government and NGO-supported government-designated PHC facilities.
- Located in Rusizi and Nyamasheke districts of the Western Province of Rwanda.
- Seeing at least 19 children aged less than 5 years per week for IMCI services in 2019 to facilitate participant recruitment (Phase 1 only, this criteria will be removed for Phase 2).

<u>Exclusion Criteria:</u>

- Secondary and Tertiary HFs (district, provincial, and specialized referral hospitals).
- Private facilities (VCT clinics, medical clinics, dispensaries).

Primary health facilities which could not be geo-located and matched with the HMIS data to assess utilization.

According to these criteria, a total of 48 PHC facilities in the two selected districts qualify for inclusion in Phase 1 of the study, including 29 health centers and 19 health posts. Of these, 32 will be conveniently selected for the parallel RCT study. The remaining six facilities that see at least 19 children for IMCI services per week, as well as the remaining smaller HFs (for a total number of up to 67 facilities) will be added during the scale-up in Phase 2. The final number of facilities is budget dependent.

[1] https://moh.gov.rw/index.php?id=547

Figure 9: Spatial distribution of all public health facilities (left) and their IMCI utilization (right). Figure was drawn in ArcGIS software by A. Kulinkina using the following data sources: district boundaries [Humanitarian Data Exchange]; health facility locations [Humanitarian Data Exchange]; utilization data from HMIS was provided by the Rwanda Biomedical Center.

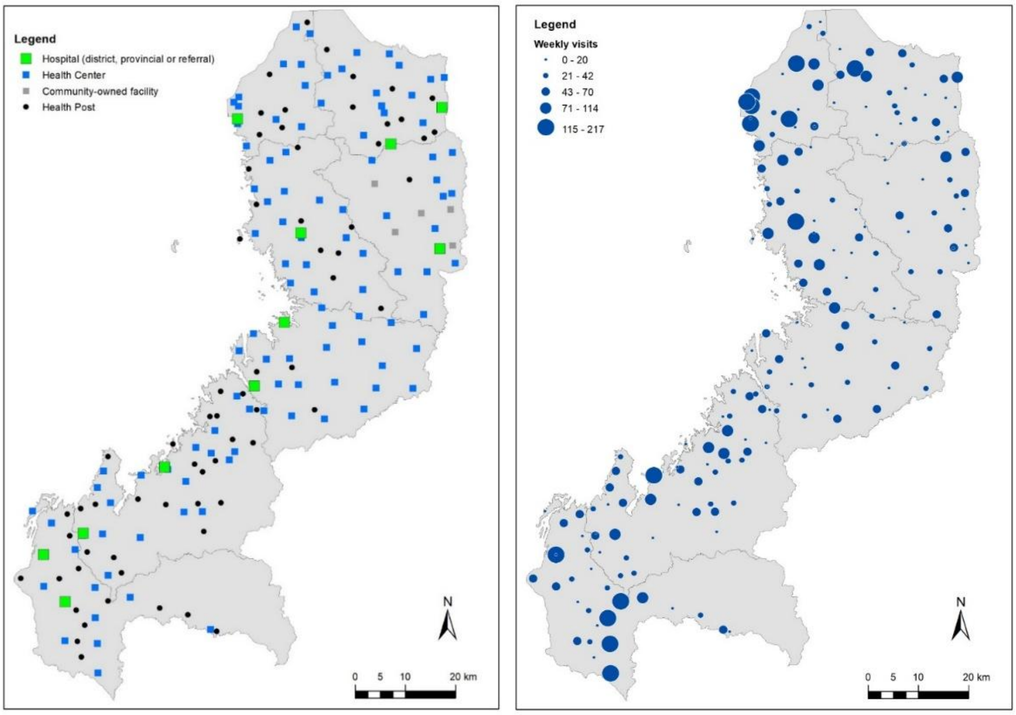

6 STUDY POPULATION

6.1 Sick children attending PHC facilities

All patients aged 1 day to 14 years (inclusive) who present to the participating PHC facilities for an acute medical or surgical problem will be included into the study. The lower limit of 1 day has been chosen to exclude peripartum conditions and immediate postnatal care, as these conditions are managed according to different guidelines and would require a specific and different tool; neonates who present to care immediately after birth (within the first 24 hours of life) will thus not be managed using ePOCT+ and will be excluded from the study. The upper limit of 14 years has been chosen to be consistent with the Rwandan age range of pediatric patients. Rwanda has a very young population, with 41.1% being children <15 years.

The algorithm does not include chronic disease management or content to inform the delivery of routine preventive health services independent of evaluation and management of an acute problem. The algorithm also does not cover routine neonatal visits.

No specification of the primary complaint or symptoms at presentation will be required for eligibility and inclusion criteria were kept as broad as possible to maximize the generalizability of our findings. Importantly, after an initial visit, any scheduled or unscheduled visit for that child that occurs within 14 days will be considered as a re-attendance visit for the same illness and excluded from primary analysis.

<u>Inclusion criteria for Phases 1 and 2:</u>

- Aged 1 day (24 hours) to 14 years (<15 years)

- Presenting for an acute medical or surgical condition

Exclusion Criteria for Phases 1 and 2:

- Presenting for scheduled consultation for a chronic disease (e.g. HIV, TB, NCD, malnutrition)
- Presenting for routine preventive care (e.g. growth monitoring, vitamin supplementation, deworming, vaccination)
- Caregiver unavailable, unable or unwilling to provide informed consent (except for children 12 or above who can provide assent with an adult witness during the consenting process).

* This does not impede the child to be managed using ePOCT+ but will lead to erasing of the electronic data at the end of the consultation and no follow-up via phone call or SMS will be made at day 7.

6.2 Other stakeholders and/or beneficiaries

Other stakeholders and/or beneficiaries will be included in mixed-methods operational research studies, including observation, in-depth interviews, surveys, and FGDs. These include HWs attending to sick children, caregivers, community members, key actors of the health system (DHMTs of Rusizi and Nyamasheke districts, HMIS department, clinical directorate staff), computer scientists at the University of Rwanda, research institutions in Rwanda involved in the ML training program, and medical experts working at the national level. Only those who have not had any interaction with the project (i.e. any of the electronic tools being implemented) or are unwilling to participate will be excluded from these additional studies.

7 OUTCOME MEASURES AND INDICATORS

7.1 Outcome measures and indicators for Objective 1: Improve the integrated management of sick children

The primary aim of the study is to evaluate whether the use of ePOCT+ for case management of sick children at the PHC level results in less antibiotic prescriptions at initial consultation as compared to routine care.

Rationale for choosing the primary aim and related primary analysis: The second-generation ePOCT CDSA showed potential as an antimicrobial stewardship intervention for managing febrile children aged 2 months to 5 years in a controlled trial setting in health facilities in Dar es Salaam in 2015-16. Management with ePOCT resulted in a drastic reduction in (unnecessary) antibiotic prescriptions while maintaining similar, or possibly achieving better clinical outcomes by day 7. The current study seeks to generate evidence that would be more generalizable to a wider population. It aims at assessing the effect of an intervention package including the extended CDSA called ePOCT+ and supporting e-learning and supervision tools, when implemented under conditions closer to those that would be encountered in routine practice versus the research setting.

Primary outcome measure for the parallel cluster study in Phase 1:

(i) % of children prescribed an antibiotic at initial consultation (as reported by HWs) in intervention facilities as compared to control facilities

Secondary outcome measure for the parallel cluster study in Phase 1:

(i) % of children cured at day 7 (according to caregivers contacted through a phone call or SMS or home visit) in intervention facilities as compared to control facilities

Main outcome indicator (monitored over the entire study period in all HFs)**:**

- % of children prescribed an antibiotic at initial consultation

Secondary outcome indicators (monitored over the entire study period in all HFs)**:**

These indicators will allow us to evaluate the effects of ePOCT+ use (versus routine care, over time, and across geographical areas) for managing sick children aged 0-14 years presenting to PHC facilities on:

Clinical outcome:
- % of children cured at day 7
- % of children with non-referred secondary hospitalization by day 7
- % of children who have died by day 7

Unscheduled secondary consultations:
- % of children with one or more unscheduled re-attendance visits at any HF by day 7

Primary referrals:
- % of children referred to hospital or admitted to inpatient ward at health center at initial consultation
- % of children who were hospitalized among those referred (i.e. referral completed)

Appropriate case management for malaria at initial consultation
- % of suspected cases tested for malaria
- % of malaria positive children prescribed an antimalarial
- % of malaria negative children prescribed an antimalarial
- % of untested children prescribed an antimalarial

Appropriate case management for other conditions at initial consultation
- % of key symptoms and signs checked and diagnostic tests performed by HWs
- Distribution of final diagnoses (including their severity) made by HWs
- Concordance between final diagnoses made and treatment prescribed by HWs
- Concordance between final diagnoses as proposed by ePOCT+ and final diagnoses by HW *(intervention period only)*

Definitions used for the outcome measures and indicators of objective 1
- *Clinical cure is a positive answer to the question "Is the child cured?" OR "Has the child improved?" (if not cured) asked to caregiver through a phone call or SMS (or home visit) at day 7. Non-referred secondary hospitalizations (see definition below) will however be considered as clinical failures even if the child is already cured at day 7.*
- *Initial consultation is the first visit of a sick child for an acute problem at a HF participating in the study (and thus registered electronically); timeframe from completion of the initial visit up to midnight of the same day.*
- *Re-attendance visit is a consultation (not necessarily at a HF participating in the study) taking place from the day after initial consultation up to day 7 included. A re-attendance visit can be scheduled (proposed by the HW on a certain day) or unscheduled (upon decision by caregivers).*
- *Primary referral is a decision of referring the child to hospital taken by the HW at the initial consultation.*
- *Secondary referral is a decision of referring the child to hospital taken by the HW during a re-attendance visit taking place from the day after initial consultation up to day 7 included.*
- *Primary hospitalization is an admission to a hospital ward taking place the same day as the initial consultation*
- *Secondary hospitalization is an admission to a hospital ward taking place from the day after initial consultation up to the phone call at day 7 included. A non-referred secondary hospitalization is a direct visit and admission to a hospital without a re-attendance visit the same day at a HF participating in the study.*
- *Antibiotic prescription is any oral, intramuscular or intravenous (but not topical) antibiotic prescribed by a HW during the initial consultation or a re-attendance visit.*
- *Antimalarial prescription is any oral, rectal, intramuscular or intravenous antimalarial prescribed by a HW during the initial consultation or a re-attendance visit.*
- *Febrile child is a child with a history of fever and/or a high temperature*
- *Malaria testing is a malaria RDT or microscopy ordered by a HW during an outpatient visit*

- *Day 7 is a range of 6-14 days post initial consultation to allow for completion of home visit for children whose caregiver reports having no phone / no phone number.*

7.2 Indicators for Objective 2: Improve the algorithm and adapt it to spatiotemporal variations

- Number of positive impact modifications in the algorithm identified by ML-based analyses
- Number of modifications implemented by type of impact (better clinical cure, less severe clinical outcomes, less medicines or diagnostic tests needed, shorter consultation time, etc.)

Definitions used for the indicators of objective 2
Positive impact modification is defined as a change in the algorithm expected to increase the clinical cure rate and/or decrease resources needed and/or to have another positive clinical or health system related impact.*
** Resources needed include but are not limited to medicines prescribed, diagnostic tests ordered by HWs, or time spent to perform a consultation.*

7.3 Indicators for Objective 3: Enhance M&E and supportive supervision

- Number of M&E indicators based on individual data that can be visualized through the medAL-monitor/medAL-outbreak dashboard
- Number of supervision and mentorship visits to HFs facilitated by the medAL-monitor tool
- Number of HWs reporting having used the medAL-monitor tool for self-auditing their clinical practices
- Number of HWs and members of DHMT reporting a positive experience with the medAL-monitor/medAL-outbreak tool

7.4 Indicators for Objective 4: Create a supportive environment for the use of clinical algorithms and supporting tools

- Number of sick children attending primary care facilities managed by HWs using the electronic ePOCT+ tool
- % of HWs able to assess key clinical signs
- Frequency of use of the medAL-monitor/medAL-outbreak tool by district team members
- Perception by HWs of the ePOCT+ tool and intervention and the number of improvements they propose for the tool or intervention
- Perception by caregivers and community members of the intervention and the number of improvements they propose
- Perception by district team members of the medAL-monitor/medAL-outbreak tool and the number of improvements they propose for the tool
- Number of medical experts able to modify a clinical algorithm using medAL-creator
- Number of computer scientists able to maintain the software and the IT infrastructure for clinical algorithms
- Number of data scientists able to analyze clinical data using ML
- Perception of the current and future utility of ML to improve health care amongst clinical experts (deciding on algorithm changes) and health authorities
- Patient and provider expenditures related to care of acutely ill children included in the study
- Carbon footprint of the intervention or carbon-saving due to the intervention

8 STUDY PROCEDURES

A flowchart summarizing the study procedures is presented in **Figure 10**.

Figure 10: Flowchart of health facilities and patients included in Phases 1 and 2

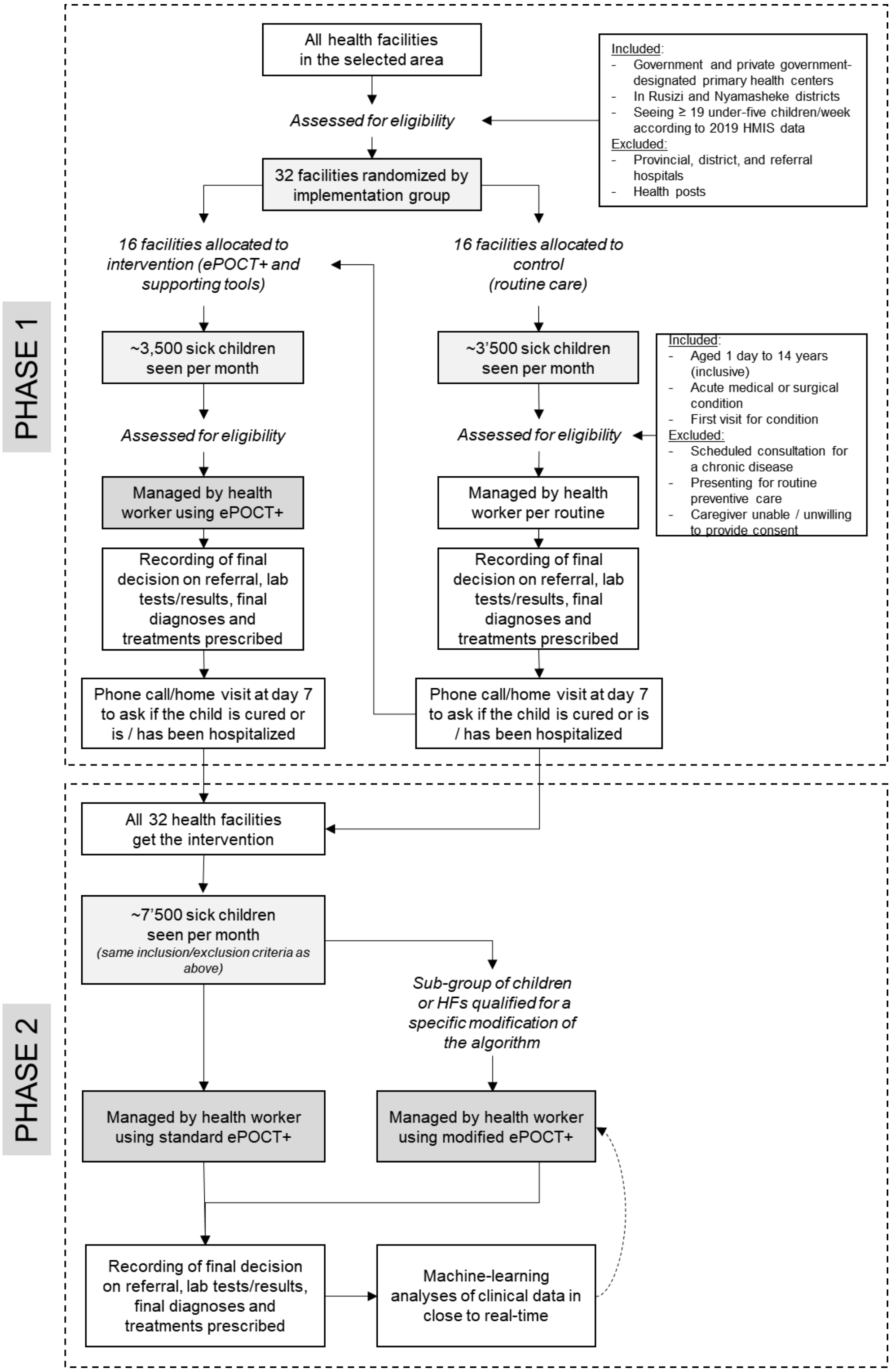

8.1 Objective 1: Improve the integrated management of sick children

8.1.1 Recruitment, screening and informed consent procedure

As the intervention is implemented at the facility level, approvals from relevant authorities will be sought before engaging with persons in charge of HFs and HWs. Sensitization meeting will be

organized in the sectors, cells and villages within the catchment areas of enrolled HFs. District and HF consent will be obtained for the intervention. HFs will be informed which group they have been selected for within the parallel cluster study design and when they will receive the intervention. HFs in groups 2, 4, and 6 will also be made aware that they will be asked to use study tablets and enroll patients to contribute baseline/control data for four months prior to receiving the intervention. Individual consent from caregivers (and assent from older children) will be sought for electronic recording of data and follow-up by phone call or SMS or home visit. Upon the start of the study, information will be provided in the waiting areas of facilities.

All patients in the HFs will be systematically screened for eligibility. All children aged 1 day to 14 years (inclusive) presenting for an acute problem to a HF that is part of the study will be asked if they wish to participate in the study by the person usually doing registration of patients or another appropriate person trained in ascertaining consent (using the registration module in the tablet). The consent process will be integrated into the routine workflow at the HFs to the extent possible. Caregivers will be asked consent for registration, electronic data recording for this and subsequent visits to the HF, and follow-up by phone or SMS or home visit for their child. They will be informed that not providing consent will not affect the medical management of their child and the child can still benefit from being managed by the ePOCT+ tool and that their data will not be used for research purposes. All caregivers will be provided a paper-based participant information sheet and consent form providing sufficient information to make an informed decision about their participation in the study. If the clinical condition of the child requires immediate treatment, this will supersede the written consent process, which will be conducted once the child is stabilized. Consent that remains at the HF will be documented electronically, either by obtaining the signature directly on the tablet, or by storing a photo of the signed paper consent form as part of the digital registration of the child. Because of the longitudinal nature of the study, a full consent process will be conducted two times throughout the study: at first visit in Phase 1 and at first visit in Phase 2. Otherwise, consent will be ascertained using a simple check-box within the ePOCT+ registration/check-in module during each visit.

A separate consent process will be used for the participants of the mixed-methods operational research studies. For example, as part of the cross-sectional quality of care surveys, trained research clinical observers will ask HWs present on the day of data collection for permission to observe a series of consultations. They will then ask the HW to request oral permission from their patient and to sign a consent form indicating their own and the patient's consent to have an observer in the room. The caregiver will also give consent to be subsequently interviewed by a different research assistant upon the completion of their consultation. Similarly, the participants of qualitative interviews or FGDs, or quantitative surveys will be recruited and asked for their consent to participate. The Informed Consent Forms associated with the study can be found in **Appendix C**.

8.1.2 Management of sick children during the initial consultation

In HFs with dedicated staff for triaging patients, sick children will first be assessed by this person using the triage module of ePOCT+ in the tablet. If the child requires urgent treatment, the triaging person will immediately inform the attending HW(s), so that he/she can give priority to that child over others waiting to be seen. If not, the child will queue normally and be attended by the next available HW. As part of triage, the vital signs will be measured (temperature, respiratory rate, weight and MUAC) as well as, if the child fulfils certain clinical criteria, oxygen saturation and heart rate using the pulse oximeter. If there are no dedicated triage staff, the triage module will be de-activated and vital signs will be measured during the consultation.

The attending HW will either manage the child using the ePOCT+ algorithm or as per routine (depending on the stage of implementation in that particular HF). HWs are ultimately responsible for the clinical management of all patients and may override the recommendations of the algorithm and provide additional or withhold treatment according to their own clinical judgment. All concomitant diagnostic interventions and treatments will thus be left at the discretion of the provider but recorded

in the ePOCT+ tool. While being managed with ePOCT+, if the child needs to go to another room for laboratory investigations (rapid tests or other), the HW will be able to suspend the data recording in the tablet and open a parallel session to attend another child in the meantime. When the child returns with the test results, the HW will resume the ePOCT+ algorithm and data recording of that child and continue the consultation normally.

At the end of the consultation, the HW (in both control and intervention facilities) will be asked to enter the final decision on referral, tests performed, final diagnoses and treatments prescribed (see Section 8.1.6 on outcomes measures). After the consultation, this information will be synced to the server as soon as there is internet connectivity.

It is assumed that usual diagnostic tests and treatments will be provided through the routine health care system. The majority of the Rwandan population, including children, are covered by a health insurance policy, which partially covers the cost of their outpatient visit (consultation cost is approximately 200 Rwandan francs but can vary depending on the socioeconomic category of the household). When not available at the HF, patients purchase their medications from private pharmacies that charge out-of-pocket. To allow testing of the intervention under conditions as close as possible to those expected for future implementation, we will not provide routine diagnostics or medications in either the control or the intervention facilities. However, we will ensure that immediate life-saving treatments are available and will support the facility staff in managing stocks through routine mechanisms. POC tests that would normally be unavailable in the PHC setting (i.e. CRP test), or available in lesser quantity than is required for the study (e.g. Hb test) will be provided to the HFs and the patients at no additional cost.

8.1.3 Re-attendance visits in case of persisting or worsening of symptoms

At initial consultation, the advice to caregivers on when to bring the child back to the HF is left at the discretion of the HW. During Phases 1 and 2, HWs will be guided by the ePOCT+ tool, which recommends conditional follow-up based on WHO and national guidelines. At re-attendance visits, whether scheduled or unscheduled, children will be managed in the same way as all other children in that particular facility at that time (ePOCT+ or routine care). If the child comes back to the same HF that uses ePOCT+ as for the initial consultation, the HW will have access to the electronic data of that initial consultation that is saved in the HF tablets. If the child goes to a different HF participating in the study, the HW will not have access to that data. Investigators will however be able to link data collected at different facilities (as long as they are part of the study) for the same child through the unique identification number provided to caregivers. If the child goes to a HF not participating in the study, no data will be available for that consultation. The ePOCT+ registration module, used at all participating facilities, will inquire about the type of visit (initial/scheduled re-attendance/unscheduled re-attendance) and the main reason for that visit.

8.1.4 Training of health workers

Following the study design, training of HWs will be rolled out in a sequential manner. Each implementation block includes facilities that will receive the intervention right away (e.g. group 1) and facilities that will start with a 4-month control period (e.g. group 2) that require different training approaches.

Before the start of the control period (applicable to groups 2, 4 and 6): All skilled HWs who are engaged in triage, assessment, and clinical management of sick children will receive training in study-related procedures, including consent for electronic recording of data and documentation of the final decision on referral, tests performed, final diagnoses and treatments using the tablet. Additionally, the research team will ensure that the standard paper guidelines (IMCI and Rwandan national treatment guidelines) are available in all HFs and HWs will be reminded that these guidelines should be followed during patient management.

Before the start of Phase 1 (applicable to all groups except group 6): HWs will receive training on how to use the ePOCT+ application on the tablet, using clinical case studies, as well as job aids for conducting and interpreting the results of POC tests which are not currently part of standard care at PHC level but are included in the algorithm (e.g. pulse oximetry and CRP rapid test). HWs will also be trained on the epidemiology of diseases in Rwanda, the most important pediatric diseases and their clinical predictors, and the importance of rational antibiotic and antimalarial use. They will also be able to review the content of the ePOCT+ algorithm and practice key clinical skills via the use of the e-learning tool. Additionally, training will be provided on the use of the medAL-monitor/medAL-outbreak tool (see Section 8.1.5).

During the month following this centralized training, HWs in control facilities will benefit from one and in intervention facilities from two visits from the implementation team for in-service face-to-face training during which trainers will accompany them in some of their consultations with real patients and help them optimize the way they use ePOCT+. Trainers will also support reorganization of workflows in the HF if necessary.

Before the start of Phase 2 (applicable to all groups): all HWs will be gathered for a brief refresher training (except group 6 will receive the full training) and to prepare them for Phase 2, where they will occasionally receive notifications that a new version of the algorithm has been released on their tablet and what that means for their management of the patients. During this refresher training, key clinical skills of the HWs will be re-assessed to evaluate whether the use of ePOCT+ and the e-learning modules has improved their clinical competencies, with anticipated differences across implementation groups associated with varying length of use of the application.

During both Phases 1 and 2, new personnel at study facilities will be trained through a dedicated module on the tablet and by their peers. This module will be available to all HWs using ePOCT+ so that they can refresh their knowledge at any time.

8.1.5 Supervision of health workers by the DHMT

In Phases 1 and 2, the DHMT will be provided the medALmonitor/medAL-outbreak tool with dashboards enabling them to monitor indicators (see **Table 3**) related to pediatric consultations taking place in the HFs enrolled in the study. Data on the child's or the HW's identification will not be provided, only the name of the heath facility will be available. Based on these indicators, the supervision team will be able to better organize on-site visits to HFs, prioritizing the lowest performing ones, knowing in advance what areas of improvement to concentrate on. The persons in charge of HFs, as well as individual HWs, will also have access to these indicators (fully anonymized except for their own data) to allow them to do self-auditing at HF level so that they are aware of their own performance in advance of receiving a DHMT supervision visit. The aim of the medAL-monitor/medAL-outbreak tool is to stimulate HWs' motivation and self-leadership to improve their skills and competencies, while enabling the supervision team to help them resolve remaining problems (Bessat et al. 2019).

8.1.6 Measurement of primary and secondary outcomes

Data captured on the tablet used by health workers during the consultation

Prior to the beginning of the control period (applicable to groups 2, 4 and 6), tablets will be provided to HWs that will contain only the patient registration and abbreviated end-of-consultation modules. Prior to Phase 1 (applicable to all facilities), the ePOCT+ application will be released onto the tablets that will contain the same patient registration module followed by the ePOCT+ algorithm that will guide them throughout the consultation process (see **Appendix A**).

To determine the percentage of children prescribed an antibiotic or an antimalarial, as well as to know the final decision on referral and final diagnoses retained by HWs, five questions will appear on the screen at the end of the consultation process, i.e. once the clinician has gone through the entire algorithm in the intervention facilities or just after the patient registration module in the control facilities. HWs will be asked to document (1) the final decision regarding the need for referral, (2) tests performed and the results, (3) date of follow-up if needed, (4) final diagnoses and (5) final treatments prescribed. For each medicine prescribed, the dosage and duration of the treatment will also be recorded (see **Appendix A**).

Data captured through phone call, SMS or home visit to caregivers

Research assistants will be trained to administer a simple questionnaire by phone to caregivers of all children at day 7 (range 6-14 to accommodate weekends and multiple attempts and home visits to those caregivers who do not have a phone or phone number) after their initial consultation with a HW of participating facilities, regardless of re-attendance visits. If the first phone follow-up fails, up to four additional attempts will be made on the subsequent days, after which they will be deemed lost to follow-up. These research assistants will be placed in a call center outside of the HFs and blinded to which facility the child has attended as much as possible. Data recorded during these phone interviews will be collected electronically and sent at least once a day to the central server. For the question related to the clinical primary outcome measure (percentage of children cured at day 7), field workers will be particularly trained not to influence caregivers when they answer the questions "Is the child cured?" and insist that they simply answer "Yes" or "No". If the answer is "No", the caregiver will be strongly advised to re-attend a HF, if possible, the one they went to for the initial consultation. Same procedure will be implemented if home visits are required using a REDCap mobile application. The questions included in the phone/SMS/home visit questionnaire are listed in **Appendix A**.

Concurrently with the personal phone call follow-up system, automated phone call and SMS systems with the same questions will be tested during the pilot or in Phase 1 in a subset of patients to check for congruency between the answers obtained using both methods. If an automated system proves successful, it will be implemented in all facilities. With the automated system, if no response is received from the first call or SMS sent to the caregiver, the research assistant will make up to two follow-up calls before the patient is deemed lost to follow-up.

8.1.7 Unique study identification number

Patients will be given a unique study identification number through a QR code that will be placed into the patient medical record booklet, vaccination card, or another document they are likely to carry with them each time they visit the HF. The ID labels will be pre-printed in bulk (specific to the district and HF) and delivered to the facilities. The label will be scanned using a QR code reader in the tablet each time the child presents at a HF. In the registration module on the tablet, an advanced search system will be created that uses the study ID number, the name of the child, the name of the mother or father or caregiver and the date of birth, so that the child can be identified with certainty with or without their ID label, and former consultations, if any, can be retrieved and linked to the new consultation. Provisions will be made to replace lost IDs if necessary and to store multiple IDs for the same patient.

8.2 Objective 2: Adapt the algorithm to epidemiological and demographic conditions

In Phase 2 of the study, the algorithm will be improved in terms of diagnostic and prognostic accuracy and adapted to local fluctuations in epidemiology, demography, geography and resource availability. Each potential modification will first be evaluated by the Rwandan clinical expert group for its clinical coherence, safety and potential benefit, and then applied to the retrospective data. If these analyses confirm a clinically relevant positive impact, the change in the algorithm will be implemented in all relevant locations/patient sub-groups and monitored based on the indicators relevant to the specific change in the algorithm collected throughout the study through the tablets.

8.3 Objective 3: Enhance M&E and supportive supervision

All data generated by ePOCT+ will be anonymized and sent to a central sever. Data will be visualized through the medAL-monitor/medAL-outbreak dashboard made available to district health authorities using the key indicators they will have chosen together with the M&E focal points (with as much overlap with the routine HMIS indicators as possible). The medAL-monitor/medAL-outbreak platform will then be installed on the PC or tablet of individuals who normally monitor data and/or conduct supervision to HFs. These users will be trained on how to use the tool and interpret trends in the indicators for decision-making. HWs and facility in-charges will also receive training on how to monitor their clinical practices at the individual and facility levels, perform self-audits, and build internal capacity and culture of quality improvement. Feedback from all users on the interface and their experience using the platform will be solicited throughout the project and the interface improved.

Furthermore, thresholds for the detection of abnormalities in the data will be integrated into the medAL-outbreak system to enable health authorities to investigate if the cause of abnormal trends in the syndromic surveillance data is due to local epidemic or another reason. For example, a sudden increase in malaria cases could be due to a problem with the quality of the batch of mRDTs used. These thresholds for the indicators of interest will be developed together with the district team to match their needs and interests.

8.4 Objective 4: Create a supportive environment for the use of clinical algorithms and supporting tools

8.4.1 Stakeholder engagement

Prior to implementation, the study will be presented to different partners and key actors in the Rwandan health sector through the Health Sector Working Group, Maternal and Child Health Technical Working Group, eHealth Technical Working Group, and the steering committee set up by the MoH to supervise and oversee the implementation of the project. The study protocol will also be presented and approved by the targeted district hospitals and regular meeting with other partners working in the targeted districts will be given regular updates on the project implementation.

Stakeholders will contribute to the content of key documents and give feedback on the planned activities. If needed, international experts can be invited at certain time points. The stakeholder group might evolve over time but the initial composition will include experts in child health, steering committee members, members of the technical working group, district hospitals and other who will be designated by the MoH and RBC.

Members of the community will also be involved throughout the study. Meetings with community leaders will be held in convenient locations to encourage participation. In initial study introduction meetings, community leaders will be informed of the study and asked to disseminate the information to their communities. Subsequently, wider community meetings will be held to solicit feedback about the intervention throughout the study period. Lastly, FGDs will allow community members to formally participate in the study.

8.4.2 Capacity building

DYNAMIC project aims to enhance partnerships with higher learning institutions to ensure the sustainability of the project and to encourage the development of highly skilled medical and research community in Rwanda. Sustainability of the intervention will be evaluated by assessing the level of local human capacities that will have been reached at the end of the study in terms of: 1) clinical competencies by HWs; 2) clinical algorithm creation by medical experts; 3) software and IT infrastructure maintenance by informatics professionals; 4) M&E capacity and use of data for decision-making by district health authorities and; 5) ML analysis by data scientists.

8.4.3 Development of frameworks for future scale-up of the e-POCT+ intervention

During the last year of the DYNAMIC project, we will develop for the Rwandan national and district health authorities, as the ultimate owners and users of ePOCT+ and its data, the necessary tools for further national scale-up. These tools will include but are not limited to:

- An optimized version of the software allowing health authorities to modify the electronic algorithms without the help of software programmers whenever they need (medAL-creator). This software application will allow clinical experts to create, maintain and update any type of algorithm aimed at guiding HWs during a patient consultation, whatever the age or medical condition. It will also include a feature to deploy or export a new version of the algorithm into all tablets located at the HFs. Through an intuitive interface, the user will be able to draw the medical algorithm by dragging and dropping some predefined elements such as exposures, signs, symptoms and diagnostic tests.
- A manual for local experts on the evidence-based path that needs to be followed to adapt and update the algorithm's medical content.
- A document on the best practices for the application of ML to clinical big data.
- A manual of implementation guidelines, standard operating procedures and training materials for various components of the ePOCT+ intervention.
- To the extent possible, interoperability guidelines will be developed for the prevailing eHealth tools with which ePOCT+ could be integrated. Examples include, but are not limited to, aggregation of patient level data from ePOCT+ for automated reporting into the DHIS2 platform, or linkage of ePOCT+ with the electronic medical records systems such as OpenClinic or OpenMRS so that information from patient encounters managed using ePOCT+ can be stored in the patients' medical record.

Support to health authorities willing to develop implementation guidelines that take into account the facilitators and barriers discovered during the project will be provided upon request. To be able to ensure maintenance of the medAL-creator open-source software in the long term, Unisanté is presently considering creating a non-profit foundation linked to Unisanté that would provide low-cost implementation consulting services for low-income countries or public entities, with the aim of reaching the largest possible number of patients with this public good.

9 STATISTICAL METHODS

9.1 Sample size calculations

For the parallel cluster study in Phase 1, the primary analysis will evaluate whether the use of ePOCT+ for case management of sick children in PHC facilities results in decreased antibiotic prescriptions (superiority analysis) at the initial consultation. Superiority in antibiotic prescription is defined as a relative decrease of ≥25% in the proportion of children prescribed an antibiotic. We estimated a cluster size of 660 patients (monthly utilization of 220 – accounting for up to 30% non-adherence from 315 – over 3 months) and an intraclass correlation coefficient of 0.025. Non-adherence here is defined as HWs not using the application for all eligible patients that come to the HF for various reasons. To have 80% power to detect a 25% reduction in antibiotic prescription (e.g. from 35% to 26%), for a one-sided hypothesis test at alpha of 0.05, we would require a minimum of 11 clusters per arm. With 16 clusters (or HFs) over 3 months, we expect a total of 10,500 participants per arm, this sample size is sufficient to test for non-inferiority in the secondary outcome (clinical cure at day 7) between the control and intervention groups.

9.2 Statistical analysis for the primary outcome measure in the parallel cluster study

Analyses will follow CONSORT guidelines (Schulz et al. 2010, Piaggio et al. 2012). A flowchart will describe the inclusion and follow-up of participants by study arm and by implementation block. Baseline characteristics and outcomes will be described by study arm and by implementation block using summary statistics such as median and interquartile range or number and percentage.

We will test for significant difference in the primary outcome of antibiotic prescription between study arms by comparing proportions and their 95% confidence intervals (CI). Primary analysis of superiority will be performed according to the intention-to-treat (ITT) principle (Committee for Proprietary Medicinal Products, 2001), with sensitivity analyses exploring differences between ITT, per-protocol and complete cases analysis approaches. The results will be stratified by sex, age group (young infants ages 1 to 59 days, children aged 2 months to 5 years, and children aged 5 to 14 years), and select clinical variables (respiratory symptoms, fever without clinical source, gastrointestinal complaints, skin problems, ear and throat problems, anemia, and malnutrition).

We will also use multi-level logistic regression with HF, patient ID, and time included as random effects (Kahan et al. 2012) to explore the effect of demographic and clinical variables on the outcome of interest. Effect modification by sex, age group, and the same clinical variables will be assessed by incorporating an interaction between arm and the respective variable, acknowledging that power will be low.

Primary analysis will be performed on initial consultations only (i.e. first visit for a particular illness episode), allowing multiple observations per patient over the course of Phase 1, but only one observation per illness. We will apply a 14-day period from the initial visit, during which no second initial visit will be possible and all subsequent visits will be marked as re-attendance by default. In the secondary sub-analyses, re-attendance visits between day 0 and day 7 will be considered, as well as longitudinal analyses of the complete visit history per patient. Further details on the primary and secondary analyses will be provided in the statistical analysis plan.

9.3 Machine learning analyses

Machine learning (ML) is a specialized branch of statistics that creates updatable algorithms, which learn from evolving data in real time. This project will make use of a specially designed algorithm-building platform, that will help clinicians explore and visualize patterns in the data collected by ePOCT+ to date. Two main types of ML methods will be used: 1) supervised classification and correlation and 2) anomaly detection. The former aims to create algorithms that predict the value of a labeled feature (e.g. the result of a malaria rapid test) using alternative data. Anomaly detection, on the other hand aims to find unusual clusters of values for a certain place, time, or person, and are the basis of outbreak detection and discovering erroneous data inputs that could be used to train field workers. These analytical techniques will be used in latter stages of the project when enough data has been collected to train the ML algorithms. Within the timeframe of the project, the analyses will be done by EPFL while identifying in-country partners and capacitating them to take on ML analyses in the future.

9.3.1 Supervised classification and correlation

Using the insights gleaned from visualizing the data, the expert clinician group will lead the generation of clinically supervised modifications to the static algorithms in ePOCT+ that aim to 1) reduce resource consumption (e.g. predicting the results of expensive or unavailable diagnostic tests), 2) improve or personalize diagnostic accuracy (e.g. creating algorithms adapted to local epidemiology, or better identifying patients who had severe outcomes in phase 1) or 3) improve the completeness of data collected by asking questions that would better predict the values that are missing. For this, the algorithm building platform will allow clinicians to explore various predictive methods, ranging from

logistic regression, neural networks and random forests. They will control the inputs required to make the prediction and decide at what threshold the predicted result will impact care decisions. The algorithm will then be updated with accumulating data to best reflect its dynamic environment.

9.3.2 Anomaly detection

Various methods allow us to identify and visualize clusters of abnormal data, by highlighting these instances, clinicians and epidemiologists can train the algorithms by labeling which are of interest and what, if any, actions should be taken when they are detected. Various methods will be explored for this aim and adapted to the type of anomaly under investigation (for example unsupervised density-based approaches such as isolation forests, and k-nearest neighbors [KNN] or multidimensional non-linear kernel-based techniques such as self-organizing maps [SOM] and support vector clustering [SVC]). The clusters deemed interesting will then be investigated for gold-standard labeling by clinicians and the surveillance team and visualizations may be exported to users of the tool for training purposes (medAL-outbreak).

9.4 Analyses of data from mixed-methods studies

Quantitative data from cross-sectional quality of care surveys, provider clinical competency or knowledge assessments, and caregiver and patient interviews will be analyzed using conventional statistical methods to compare means (t-tests or paired t-tests) or frequencies (chi-square tests) across intervention groups or time periods. Regression analysis may also be used to explore associations of multiple predictors with outcomes of interest.

Qualitative interviews and focus group discussions will be audio-recorded and detailed notes will be taken. Records will be manually transcribed in their original language and translated into English. A qualitative data analysis software (e.g. Dedoose, Nvivo, ATLAS.ti) will be used to code the English interview transcripts in order to identify emerging themes. A predefined list of codes based on the interview guide and the recurrent themes highlighted in the detailed notes will be used. New codes will be created when new themes are identified during more detailed analysis of the transcripts. Deductive coding will be used to extract the themes based on the interview guide, but inductive coding will also be used to extract any unexpected themes that arise during the interviews.

9.5 Estimated number of beneficiaries

Throughout the study, we expect that an average of 212 children per month per HF will be managed using ePOCT+. In Phase 1, the number of direct beneficiaries include children attended to using ePOCT+ (n=24,000) and HWs using ePOCT+ (n=120) for a total of 24,120 people. Indirect beneficiaries may include caregivers and immediate family members who are less exposed to infectious diseases as a result of better cure rates of their child/sibling. Using an average rural household size of 4.3 people according to the 2012 Census, the number of expected indirect beneficiaries is approximately 151,368 (i.e. 3*50,456, excluding the sick child who is a direct beneficiary). The actual number is likely to be lower due to the possibility of multiple visits per child over the course of Phase 1.

In Phase 2, we expect a maximum of around 48,000 consultations over 6-7 months. The number of direct beneficiaries will be less than this estimate, because children may come to the facility more than once, either for the same illness or a different illness over the course of the study. In Phase 2, approximately 200 HWs are expected to directly benefit from using ePOCT+ and their clinical decision support and e-learning functions. Additionally, approximately 10 users of medAL-monitor/medAL-outbreak at the district and national levels will also benefit directly. Lastly, all individuals who are capacitated IT systems management, ML, adaptation of the algorithm through medAL-creator, etc. will be included as direct beneficiaries (n=50). Because of high coverage of the intervention in Phase 2, indirect beneficiaries will be the entire population of the intervention districts (n=783,000) as a result of 1) less exposure to infectious diseases due to better cure rates of sick children; 2) reduced drug

pressure and likelihood of antibiotic resistance due to reduced antibiotic prescriptions; and 3) reduced risk of illness due to more effective outbreak detection and response.

10 INFORMATION TECHNOLOGY, DATA MANAGEMENT AND MONITORING

10.1 Overall IT solution

The overall IT solution is provided by several components; some of them have been already discussed or presented previously, the diagram (**Figure 11**) below is the complete overview of the solution. From left to right we have the following components:

medAL-creator (Medical Algorithm Creator): The clinical algorithm management system that will allow to manage, reference, modify and deploy the different versions of the algorithm into a mobile device. medAL-creator is hosted in Switzerland and managed by the Unisanté IT team.

medAL-reader (Medical Algorithm Reader): The application (tablet or PC) that will contain the algorithm and be used for data acquisition by the HW. medAL-reader could be deployed in a standalone or multi-device mode. In a standalone mode the application will receive updates of the algorithm directly from medAL-creator.

medAL-hub: The component that enables ePOCT+ to work in a multi-device environment. It plays a role of a central database at the HF level. This component will receive the updates of the algorithm from medAL-creator and pass them on to medAL-reader (i.e. the tablets). It will also collect the data from medAL-reader and push it to medAL-data.

Data Management System: this component will be considered as the data hub of our solution. It will be hosted on a server in a secure data center in Kigali:

- medAL-data: This is the data collector for the country, it plays several roles:
  - Data collector
  - Data manager (Clean, Quality Check, Audit trail, Anonymization)
  - Data publisher
  - Data monitoring
- medAL-monitor/medAL-outbreak: Targeted supervision to HFs by the DHMT will be organized based on the quality of care indicators calculated in real time from the uploaded data and made available through a web-based dashboard

Research Database: This is a database only with anonymized data where the machine learning team will do their analyses. This database may be hosted in Switzerland where ML analyses will be performed by the EPFL team, but overtime can be done on the medAL-data server in Rwanda.

Figure 11: IT architecture. Icons were obtained from open sources: https://healthicons.org/ and https://openclipart.org/.

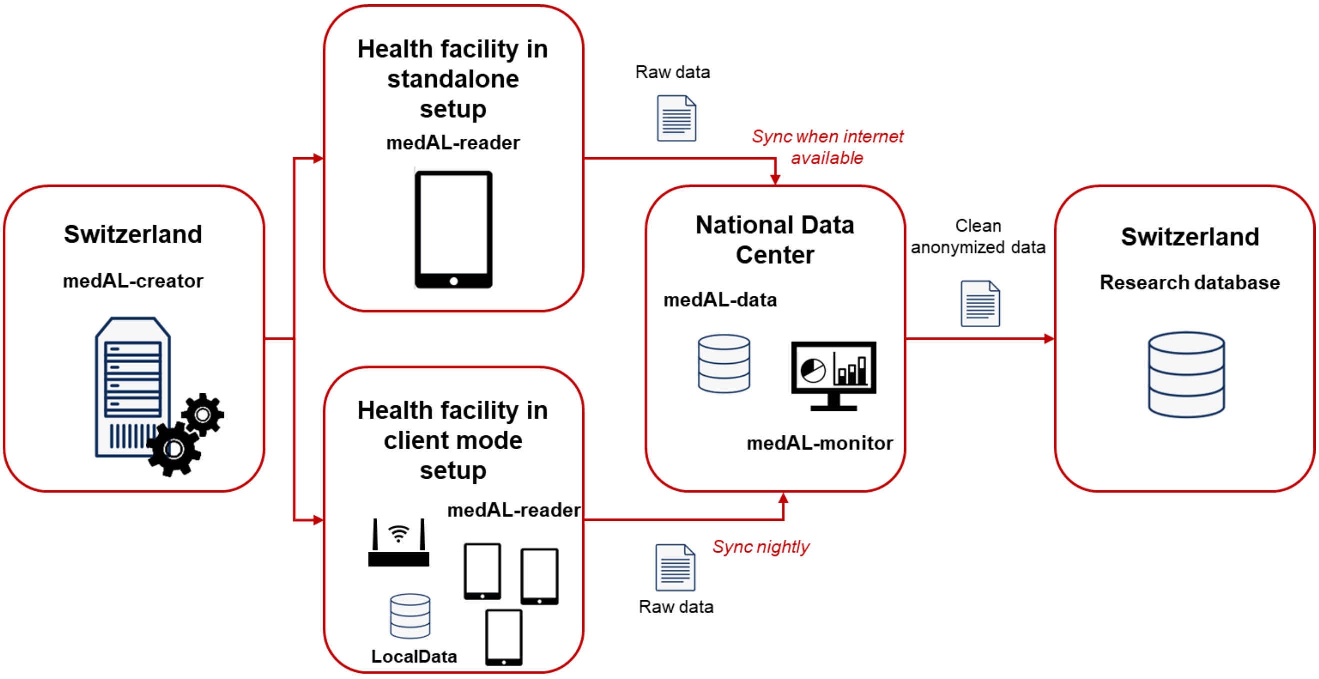

10.2 Data flow, storage and transfer

The data flow for data collected via the ePOCT+ application is shown in **Figure 12**. Data collection starts at the HF where the study clinicians capture the data using the mobile application. When an Internet connection is available, ePOCT+ (or medAL-hub in multi-devices set-up) will sync the data with the data management system hosted on the data server in Kigali (medAL-data) in the same data center that hosts other national government and partner data systems. The data management team will monitor, clean and anonymize the data in this system and make the anonymized data available for the Unisanté IT team, after signing the data sharing agreement. The Unisanté IT team will monitor the data received, communicate the possible errors or anomalies before sending them to the EPFL ML team for ML-based statistics. Data for the operational research studies will be collected and managed by a secure third-party cloud based EDC platform.

Figure 12: Data flow

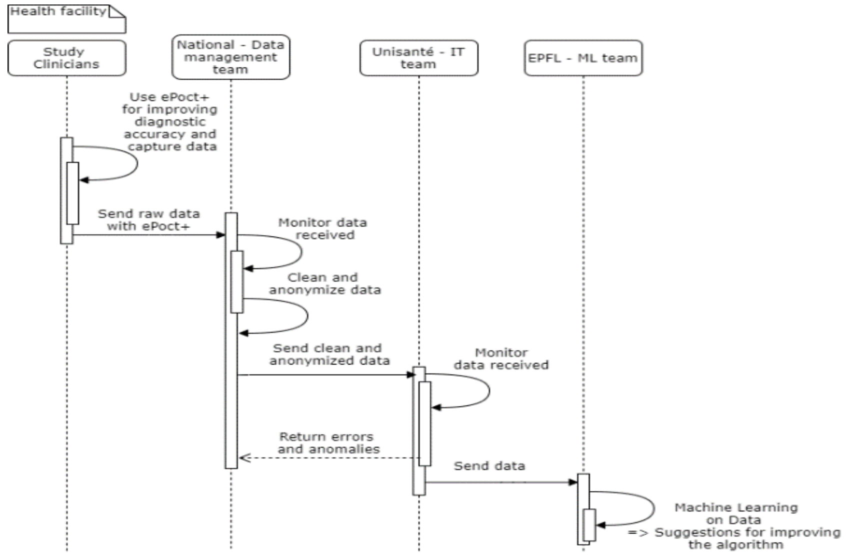

10.3 Data security and privacy

Swiss TPH Rwandan local team, working with Unisanté, in collaboration with the local health management teams, will be responsible for ensuring the privacy and security of all data collected during the project, especially with respect to individual patient data. Our methods are designed to comply with international and Rwandan human rights standards that apply to regulating patient data protection, ownership, storage and processing.

Primary data collection devices:

Tablets used for primary data collection will be password-protected and assigned to individual HFs and/or HWs. A secure PIN will be necessary on each request to view an existing patient file. When not in use, the tablets will be locked and stored in physically secure location.

Transfer of data to secure local servers:

All data uploaded from the primary data collection devices to the local server in Kigali will be encrypted using an SSL connection. All raw data will remain on the secure local servers in Kigali and will not be exported as such.

Anonymization and machine learning IPD privacy:

Before being transmitted to Switzerland for ML analysis or the creation of reporting metrics, automated anonymization algorithms will remove identifying features of the raw data. Features removed will include names, dates of birth, addresses and identification numbers. At no point will these confidential data attributes be exported outside of the secure local server.

Transfer of data from local to Swiss servers:

When transferred from Rwanda to Switzerland, the data will travel through a secured VPN connection. Data Transfer Agreements (DTA) will be filled by all partners prior to any data transfer.

Additionally, ML analyses done by collaborators at EPFL will explore new techniques of identifying additional feature combinations that put patient privacy at risk. We will also investigate means to export elements of the data in a manner that renders it impervious to the latest re-identification attacks, for instance, by adding carefully calibrated noise (differential privacy) or by advanced encryption. Finally, we will also explore the possibility of decentralized learning that will allow analyses to take place without data transmission.

10.4 Data quality assurance

To minimize data recording errors by HWs on the tablet, ePOCT+ data fields will include validations that limit the values to plausible ranges. Cleaning and quality assessment of the anonymized raw data arriving on the server in Kigali first be performed by the data manager using predefined algorithms developed in collaboration with the Swiss data management team and documented in the Data Management Plan. Pre-defined automated high-frequency checks will be performed including checks of completeness and indicators of other irregularities such as completion time. Reports will be shared with the field implementation team. Systematic missing or inconsistent data coming from HFs will be discussed first with the DHMT of the corresponding district and/or the facility in-charge, as appropriate. A quarterly data review meeting will be organized by the study team along with a representative of DHMTs of each district to discuss any outstanding data quality issues. Queries related to data captured through day 7 phone calls will be brought to and solved directly with the research assistants performing the calls. Call data will be audited through random checks of phone logs and repeat phone calls.

Regular quality checks will also be performed on the cleaned anonymized data sent to the Unisanté server by the Swiss team and queries sent to and solved with the Swiss TPH data management team in Kigali. The database will be locked after all data has been verified and all raised queries have been resolved. Conventional and ML analyses will then start to be performed on this locked database and results pushed to the medAL-monitor/medAL-outbreak platform.

11 ETHICAL CONSIDERATIONS

11.1 Participant information and consent

Inclusion of patients in the intervention study, as well as caregivers, HWs, community members or DHMT members in the mixed-methods studies, will occur only if the participant has provided written informed consent. Written informed consent will be obtained prior to data collection, all non-routine clinical assessments, and any other study-related activities.

District and HF consent will also be obtained for the intervention. Information and consenting tasks for sick children and their caregivers may be performed by any HF staff (for more details on the informed consent procedure, see Section 8.1.1). Information and consenting tasks related to mixed-methods studies will be performed by the research scientists and their study team members. All these individuals will undergo adequate training with a special emphasis on the process of unbiased information of study participants ensuring that non-participation in the study does not result in a child not being adequately managed. During this training, theoretical and practical sessions will be devoted to research ethics and informed consent procedures.

The participant information sheet/informed consent documents will be available in English and Kinyarwanda. These documents are attached to the present protocol (see **Appendix C**).

11.2 Risk-benefit assessment

Patients and caregivers:

The proposed intervention is based on international and national treatment guidelines, supplemented by POC tests that are routinely used for managing sick children at hospitals. As such, we do not anticipate that the implementation of this intervention will pose a risk to patients. We rather anticipate that the implementation of ePOCT+ will provide a direct benefit to patients by improving case management of sick children at PHC level. With reduced antibiotic prescriptions, we anticipate children to develop healthier immune systems, making them less likely to be sick in the future. Additional benefit to patients and their caregivers is the reduced cost of health care due to fewer re-attendance visits and secondary admissions.

Community:

Communities in the intervention area are also expected to benefit from the project in several ways. Reduced antibiotic prescriptions will reduce antimicrobial drug pressure in the community, and hence the risk of drug resistance. Better cure rates are expected to lessen the exposure to infectious diseases in the community.

Health workers:

HWs are expected to develop better clinical skills and practices through the use of ePOCT+ and the associated training and mentorship provided through the project. The data generated with ePOCT+ will produce an opportunity for their work to also be audited by their supervisors and the DHMT. In some circumstances, this could pose some perceived or actual risk to their job security. All data available to higher managers or authorities will be de-identified to mitigate these risks as much as possible.

District health authorities:

The DHMT will be able to monitor the quality of the consultations performed by HWs and plan better for their supervision and mentorship visits to HFs. To ensure that this insight into their daily work is not perceived by HWs as external control but rather a support mechanism for them to improve their competencies and skills, they will have access to their own data and will be encouraged to perform self-auditing before the supervision visit by the DHMT, so that a fruitful and respectful discussion can take place during the visit. This strategy has already been shown to be very well accepted by HWs in Burkina Faso during the implementation of a CDSA in routine conditions at primary care level (Bessat et al. 2019).

Central health system:

The parallel cluster study will generate evidence about the impact of ePOCT+ under routine (versus experimental) conditions. This will support the development of guidelines for potential national scale-up of the intervention, and similar digital health tools, in the future. The economic evaluation will provide additional information on the overall costs of healthcare provision with and without the use of ePOCT+. Lower costs of medicines are anticipated, which equates to lower costs of healthcare provision at the central level.

The project overall:

The project provides many benefits, as described above, but also comes with some risks. For example, clinicians may be hesitant to use the tool, because it is difficult to use or they feel it is too prescriptive, etc. As CDSAs cannot cover all possible clinical situations, even in a well-defined patient target group, HWs are ultimately responsible for the clinical management of all patients and may override the recommendations of the algorithm and provide additional or withhold treatment according to their own clinical judgement. To mitigate this, qualitative research will be conducted to solicit user feedback and facilitate improvements to ensure that the ePOCT+ application meets user needs. Furthermore, the application may be perceived by some stakeholders as a “black box” and be wary of the use of machine learning for algorithm adaptation, which may reduce their trust in the system. To ensure full transparency, as requested more and more by stakeholders (Ansermino et al. 2019), the medical content of the algorithm used during Phase 1, as well as any modification introduced during Phase 2, even if applied only in some geographical areas or for a short period of time, will be published in real-time on the website dedicated to the DYNAMIC study. Health authorities and HWs will thus always have access to this content and know the exact combinations of clinical predictors used to suspect or diagnose each disease. No change in the algorithm will take place automatically and all modifications will be introduced manually. ML will be used only in the background, to complement conventional statistical methods to analyze data and guide decisions of the clinical expert group. For safety reasons, ML will never be used within the algorithm. Lastly, in Phase 2 of the study, when the intervention is introduced in all or nearly all HFs in the intervention area, there will be no

control group. There is a possibility that due to circumstances that are external to the project (e.g. severe weather events, food insecurity, disease outbreaks) childhood morbidity and mortality could increase. In order not to incorrectly attribute poor outcomes to the study, we will need to monitor these events as closely as possible.

11.3 Internal monitoring

Monitoring of study procedures will be done by the implementation team through on-site and remote activities. The main goal of monitoring will be to ensure data quality and the wellbeing of study participants. Screening data, consent, recruitment rates and missing data on primary and important secondary endpoints will be reviewed on a periodic basis. Findings of remote monitoring may influence on-site monitoring. Summary reports will be provided to the research steering committee.

The study monitoring team will be responsible for checking if the intervention tools are in place and used for the purpose for which they have been designed (e.g. that informed consent is obtained correctly, or that ePOCT+ is used during the consultation and that data are not entered retrospectively after the consultation is finished) and that there are no major issues impeding basic clinical procedures (e.g. stock out of antimalarials or antibiotics for weeks). They will do so in a way that does not interfere with decisions taken by HWs so as not to introduce bias.

The DHMT will also play an active role in the monitoring of the project. All data related to child health or quality of the consultation, such as compliance with the algorithm by HWs, are outcomes and indicators that will be visualized in the medAL-monitor/medAL-outbreak tool and regularly reviewed by the DHMT. The study team will facilitate regular meetings with the district teams to discuss major problems related to the study, the intervention, or the tools (that may jeopardize the study) and propose solutions if any. The study team will accompany the DHMT during some of their supervision visits to monitor how they use the medAL-monitor/medAL-outbreak tool and how HWs are able to perform self-auditing. A detailed monitoring plan will be developed before the start of the study.

11.4 Premature termination of study

The Principal Investigators may decide to terminate the study prematurely according to certain circumstances, such as ethical concerns that cannot be resolved, in case of safety of the participants being compromized (e.g. if the benefits no longer outweigh the risks), alterations in accepted clinical practice that make the continuation of the study unwise, or evidence of harm of the intervention. In case of premature study termination, the National Ethics Committee will be notified as per local guidelines.

11.5 Local regulations, declaration of Helsinki and protocol amendments

The research project will be carried out in accordance with the research plan outlined in this protocol, principles enunciated in the current version of the Declaration of Helsinki, Essentials of Good Epidemiological Practice issued by Public Health Switzerland, and all national, legal, and regulatory requirements as applicable. The DYNAMIC study will also be registered on ClinicalTrials.gov.

Prior to the field activities, ethical approval will be obtained from all the concerned institutions. The study protocol will first secure approval of the Rwanda National Ethics Committee. The project will then be presented to and approved by the Rwanda Maternal and Child Health Technical Working Group. Lastly, the project may also need to be approved by the National Health Research Committee of the Rwanda MoH. After all the approval processes are completed, other institutions and line ministries like Rwanda Food and Drugs Authority, MoH, Ministry of local government and targeted districts will provide collaboration letters and the project management will regularly update those institutions about the study activities.

All protocol modifications will be documented. A protocol amendment can be initiated by either the Sponsor or any Investigator. The Investigator will provide the reasons for the proposed amendment in writing and will discuss with the Sponsor and the Swiss and Rwandan Principal Investigators. Any protocol amendment must be approved and signed by the Sponsor and the Principal Investigator and will be submitted to the National Ethics Committee for information and approval. Approval will be obtained before any changes can be implemented, except for changes necessary to eliminate an immediate hazard to study participants, or when the change involves only logistical or administrative aspects of the study. An annual report by the national investigators will be submitted to the National Ethics Committee per national guidelines.

11.6 Alignment of the project with national priorities

The project is aligned with Rwanda's national priorities to promote health research in its economic transformation process. The vision 2020 and 2050, and the Economic Development and Poverty Reduction Strategy (EDPRS) acknowledge that ''a healthy population is vital if the country is to achieve sustainable economic growth''. The main objectives of the DYNAMIC project are in line with the EDPRS priority 2 – to increase quality, demand and accessibility of primary health care in order to achieve economic growth and decrease under five mortality by 2030. The project is also in line with the National Action Plan for Human Security in Rwanda that includes advocacy and priority-setting regarding the mitigation of AMR.

Furthermore, the 4th Health Sector Strategic Plan emphasizes the expansion of ICT and e-health research within the health sector and increasing the availability of web-based training and educational opportunities for the health workforce. Additionally, Rwanda is currently in the process of expanding its Medical Records System in the OpenMRS platform, including at the primary care level and specifically within the child health subject area in collaboration with UNICEF. While OpenMRS is focused on creating and maintaining patients' medical records with some ability to incorporate clinical guidelines and alerts, ePOCT+ can provide complementary more advanced clinical decision support functions. It is the intention of the DYNAMIC project to fully explore interoperability and data exchange possibilities with OpenMRS and other e-health platforms used in Rwanda so as to improve (rather than complicate) the workflow of HWs and reduce duplication of effort and data entry.

12 ORGANIZATION AND TIMELINES

12.1 Organizational structure

Unisanté is the leading institution in close collaboration with Swiss TPH, Swiss TPH local office in Kigali, Rwanda Biomedical Center, and the Machine learning and optimization laboratory of EPFL.

The Project Technical Committee (PTC) will ensure an appropriate framework to achieve project objectives, take necessary decisions to effectively implement the workplan and achieve its deliverables, by allocating funds and resources according to activities and milestones. The local implementation team based in Rwanda will ensure implementation of all activities in the field in the Western Province, with the support of the different technical leads based in Switzerland.

The Project Management team will coordinate and monitor activities against the implementation plan; negotiate and administer funds; organize meetings and teleconferences; coordinate report writing; ensure effective communications between partners; monitor milestones and deliverables; and coordinate the dissemination of results obtained by the project to the scientific community (**Figure 13**).

The National Stakeholder Group will review and discuss progress of ePOCT+ implementation, give feedback on the annual project reports, provide guidance on aligning activities with national priorities, call upon harmonized specialized technical assistance in key areas when necessary, identify, discuss and propose solutions for possible problems related to the intervention and/or the health system.

Figure 13: Organizational structure for the project

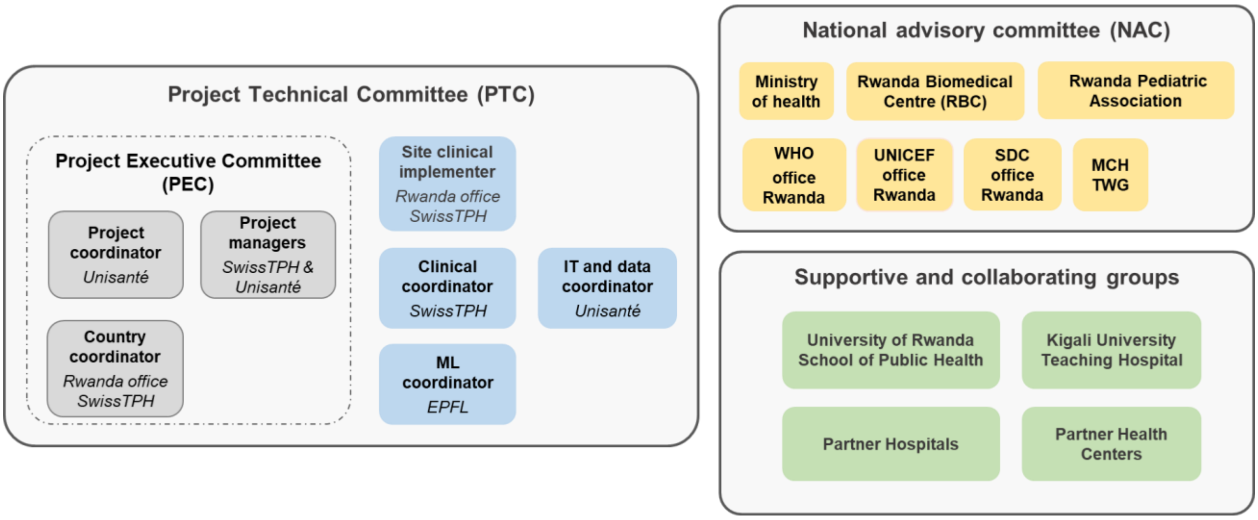

12.2 Roles of investigators and collaborators

Unisanté will act as the main sponsor for the study. The principal investigators and co-investigators at RBC, Unisanté and Swiss TPH are responsible for the overall design and conduct of the study. This includes assuring that the study is conducted in accordance with the protocol, the rights, safety and welfare of the study participants are protected, local requirements of the ethical committee are adhered to, adequate training of site personnel and integrity of study data are maintained. The team from Switzerland (Unisanté and Swiss TPH) will provide epidemiological, statistical, IT, administrative and financial management support.

Prof. Valérie D'Acremont will serve as the overall scientific lead and the key contact for study design and writing of the protocol. She will lead the process of documenting findings and lessons learnt through the project. She will oversee the development and writing of scientific manuscripts, as well as technical and financial reports. She will lead writing of progress reports and dissemination of results to partners and stakeholders.

Dr. Sabin Nsanzimana will oversee the study conduct in Rwanda. He will coordinate the inputs of other RBC colleagues and facilitate contacts with project stakeholders at the national and district levels.

Dr. Alexandra V. Kulinkina will be the overall project coordinator who will coordinate activities between the different groups involved in the study in Switzerland and in Rwanda and provide technical assistance to the local implementation team. She will support the PI in all her activities and lead the development and writing of scientific and technical manuscripts, as well as the writing of progress and financial reports and dissemination of results to partners and stakeholders.

Cassien Havugimana will coordinate and oversee the implementation activities throughout the project and serve as the main contact for the National Stakeholder Group. He will coordinate and contribute to the writing of progress reports, scientific manuscripts and dissemination of results to partners.

Dr. Ludovico Cobuccio will be responsible for providing technical support to all activities required to implement the ePOCT+ tool at HF level and the medAL-monitor/medAL-outbreak tool at district level in the Western Province. He will lead the development and approval of the ePOCT+ algorithms by the medical experts and coordinate and monitor the implementation of ePOCT+ in HFs. He will contribute to the writing of progress reports, scientific manuscripts and dissemination of results to partners.

Dr. Theophile Dusengumuremyi will lead the implementation of the ePOCT+ tool at HF level and the medAL-monitor/medAL-outbreak tool at district level. He will also coordinate the country adaptation process of the ePOCT+ algorithm by the child health expert group. He will contribute to the writing of progress reports, scientific manuscripts and dissemination of results to partners.

Francine Bayisenge will support the overall field implementation of ePOCT+ and training of health workers, lead the qualitative studies, and contribute to the writing of progress reports, scientific manuscripts and dissemination of results to partners.

Vincent Faivre will lead the development and maintenance of the software and assess the IT infrastructure needed with the local IT team. His team will also develop the necessary tools for the data collection and management. During the data collection, he will also coordinate data verification, in close collaboration with the IT and the in-country data management team.

Serge Zimulinda will coordinate and support the IT needs of the project. He will make sure that the infrastructure for the data collection is operational, secure, maintained and backed up. He will also make sure that an issue management system is operational for facilities to report their problems with the tablets and the IT infrastructure.

Gilbert Rukundo will coordinate the field data collection and serve as the data and quality manager for the project. He will work closely with the field team to resolve data quality issues as they arise. He will contribute to the writing of progress reports, scientific manuscripts and dissemination of results to partners.

Martin Norris will serve as a project manager for implementation activities. He will also support the data systems for the project.

Aicha Yusuf will assist in project management and organization of the field activities as well as support with clinical training and supervision of health workers.

Dr. Victor Rwandarwacu will support the implementation activities and mentor health workers in the field.

Dr. Marie-Annick Le Pogam will provide guidance on the study design and statistical considerations during the analysis of the results.

Alan Vonlanthen will ensure project coordination and communication between the different teams (clinical, data & IT, ML and project management) involved in the project.

Dr. Mary-Anne Hartley will lead the machine learning analyses, with the support of Prof. Martin Jaggi, as well as the related preparation of progress reports, technical and scientific manuscripts, and dissemination to partners and stakeholders. They will also organize knowledge transfer to professionals in Tanzania willing and able to learn the use of applied machine learning for clinical interventions in resource-limited settings.

Prof. Kaspar Wyss will advise on the larger context of the project and how it fits into health systems strengthening activities. He will also help facilitate stakeholder engagement and relations.

Dr. Pacifique Ndishimye and Noella Bigirimana will advise on locally appropriate research methods and innovation.

Dr. Albert Tuyishime and Eric Gaju will advise on the national eHealth landscape, progress of other national eHealth initiatives, and help facilitate integration of ePOCT+ into the national data systems.

The Sponsors, Investigators, and key study members as outlined above have all contributed to the writing of the present protocol. They may delegate tasks and responsibilities as appropriate but will provide sufficient oversight over all tasks delegated and will ensure that each individual, to whom a task is delegated, is qualified by virtue of education, training, and experience to perform each of their delegated tasks.

12.3 Timelines

The DYNAMIC project started on the 1st December 2019 and will cover a three-year period up to 31st December 2022. Phase 1 (excluding the pilot period) will last from April 2021 to April 2022, Phase 2 from June 2022 to December 2022, implementation research from April 2021f to October 2022, and assessment of sustainability for scale-up from August 2022 to December 2022. The project timeline is shown in **Figure 14**.

Figure 14: Project timeline

	2020						2021												2022											
	7	8	9	10	11	12	1	2	3	4	5	6	7	8	9	10	11	12	1	2	3	4	5	6	7	8	9	10	11	12
**Project phase**										**Pilot**					**Phase 1**									**Phase 2**						
**Tools implementation**																														
Sensitisation meetings				x	x	x	x	x	x																					
HCWs in person group training										x				x	x		x	x		x	x									
Onsite face-to-face training										x				x	x		x	x		x	x									
e-learning platform																		x	x	x	x	x	x	x	x	x	x	x	x	x
ePOCT+ stepped-wedge study															x	x	x	x	x	x	x	x								
ePOCT+ scale-up (if feasible)																								x	x	x	x	x	x	x
Modifications of ePOCT+																								x	x	x	x	x	x	x
Clinivisor/eMergence tool																		x	x	x	x	x	x	x	x	x	x	x	x	x
**Data collection**																														
Consultation data										x	x	x	x	x	x	x	x	x	x	x	x	x	x	x	x	x	x	x	x	x
Follow-up data										x	x	x	x	x	x	x	x	x	x	x	x	x	x	x	x	x	x	x	x	x
Cross-sectional surveys									x	x								x	x	x	x	x				x	x			
Qualitative studies																		x	x	x					x	x	x			
**Data analysis**																														
Clinical data										x	x	x	x	x	x	x	x	x	x	x	x	x	x	x	x	x	x	x	x	x
Qualitative data																			x	x	x					x	x	x		
**Dissemination**																														
Publications and reports								x	x						x	x									x	x			x	x
Dissemination of findings																										x	x	x	x	x
Framework for scale-up																										x	x	x	x	x

13 CAPACITY BUILDING, KNOWLEDGE TRANSFER AND DISSEMINATION PLANS

The proposed project offers many opportunities for capacity building and transfer of technology, which are summarized in

Table 7.

A website dedicated to the DYNAMIC project will be set up to publish the medical algorithm and its modifications in real time. Regular news on the status of the project, teaching material and tutorials developed for the project and preliminary results of studies will also be made available on this website.

As soon as they are available, the final results of the parallel cluster randomized controlled study of Phase 1 will be presented to and discussed with the DHMT as well as with the staff of all HFs participating in the study. They will also be presented and discussed with the communities of the catchment areas of these HFs. The same will be done again with results of studies in Phase 2. National dissemination meetings will be held to share the gained knowledge among all stakeholders in Rwanda. Reports for stakeholders and funders will be submitted annually and/or upon reaching key milestones in the project.

Results from this project will be published in open-source peer reviewed scientific journals and presented at relevant conferences and during symposia at national and international level, thus contributing to the dissemination of knowledge gained for the benefit of stakeholders worldwide involved in child health, digital health, disease surveillance and the use of ML in the health sector.

Table 7: Areas of anticipated capacity building and technology transfer by health system level.

**Area of capacity building/knowledge transfer**	**Health system level**		
	**National**	**District**	**Health Facility**
Clinical knowledge	Evidence-based clinical guideline development and continuous update of clinical content through the involvement of key pediatric stakeholders in the development of the ePOCT+ content<br>Increased capacity of Rwandan MDs in clinical algorithm validation and translation into health policy	Increase in medical knowledge through supportive supervision visits by study personnel<br>Supervision at HF level through the development and implementation of medAL-monitor/medAL-outbreak	Improved clinical skills of HWs through the use of ePOCT+ and e-learning modules
Information technology		Set-up and maintenance of IT infrastructure through initial capacity building and continuous technical support from study team	Set-up and maintenance of IT infrastructure through continuous technical support from study team
Software development and maintenance	Development and maintenance of ePOCT+ software through involvement of Rwandan software engineers	Maintenance of software at the district level through continuous capacity building of the district IT team throughout the project	Knowledge in the use of electronic tools for patient management

Database set-up and management	Set-up and maintenance of the database		
Data interpretation and use for decision-making	Definition of relevant indicators through ownership of database by MoH and technical support	Management and interpretation of health data shown in medAL-monitor/medAL-outbreak dashboards through technical support by study team	Improved ability of HWs to interpret data due to information provided through medAL-monitor/medAL-outbreak
Machine learning	Use of ML to analyze health data through involvement of Rwandan partners in ML activities and a training program		

14 BUDGET

The budget for the DYNAMIC project administered from Rwanda is 1,799,200 Swiss francs (CHF), which is equivalent to approximately 7,150,000 Rwandan francs (FRw). **Table 8** breaks down the budget by project year (1st December 2019 through 31st December 2022) and project activity.

Table 8: Total budget in Swiss francs.

	Year 1	Year 2	Year 3	Total
Staffing	262’000	379’000	316’000	957’000
Training and workshops	42’500	27’500	12’500	82’500
IT infrastructure and equipment	45’750	5’000	3’000	53’750
Transportation	40’350	40’350	71’400	152’100
ePOCT+ implementation (Phases 1 and 2)	30’000	164’050	148’800	342’850
medAL-monitor/medAL-outbreak implementation	5’000	5’000	5’000	15’000
Mixed methods operational research	5’000	5’000	5’000	15’000
Stakeholder meetings and dissemination	45’000	40’000	36’000	121’000
Administrative costs	20’000	20’000	20’000	60’000
**TOTAL**				**1’799’200**

Budget justification

Staffing costs include salaries of all Swiss TPH local office in Kigali who are fully or partially allocated to the DYNAMIC project, as well as any temporary staff hired specifically for the project.

Training and workshop costs include rental fees for meeting venues, development and printing of all training materials, and per diems/allowances for meeting participants.

IT infrastructure and equipment costs include the procurement of all tablets, computers, servers, routers, and other items required to establish connectivity between tablets in study facilities and with the main server. This category also incudes the set up and maintenance of the follow-up system via phone calls or automated SMS system.

Transportation costs include hiring or purchase and maintenance of vehicles, motorbikes, and other means of transportation between the study sites and/or to all the HFs for data collection or monitoring associated with all study activities. Driver salaries are also included in this category.

ePOCT+ implementation costs include the procurement of POC lab tests and medicines (if needed), printing of labels for identifying patients, salary top-ups for HF staff that perform additional duties for the study (if needed), internet bundles (if needed), and other miscellaneous expenses.

medAL-monitor/medAL-outbreak implementation costs include meetings and consultations with the users and relevant stakeholders regarding the content and visualization of the data, regular visits to district health officials to build capacity in interpreting the dashboards, and facilitating regular district visits to HFs for mentorship and supervision of the HWs.

Mixed methods operational research costs include the development of finalization of study tools, field data collection, software for data analysis, and the hiring of temporary field enumerators.

Stakeholder meetings and dissemination costs include rental fees for meeting venues, refreshments, development and printing of materials, reimbursement of transportation costs or per diems/allowances for meeting participants (if needed). Also included in this category are publication fees and development of dissemination materials at the end of the study (e.g. pamphlets, booklets, etc.).

Administrative costs include office space rental fees, office supplies, fees for various approvals, and other miscellaneous expenses.

15 REFERENCES

- Asermino JM Wiens M, Kissoon N. Evidence and Transparency are Needed to Develop a Frontline Health Worker mHealth Assessment Platform. Am. J. Trop. Med. Hyg., 101(4), 2019, p. 948.
- Benoun, J. M., J. C. Labuda, and S. J. McSorley. 2016. 'Collateral Damage: Detrimental Effect of Antibiotics on the Development of Protective Immune Memory', MBio, 2016: 7.
- Bessat C, Zonon NA, D'Acremont V. Large-scale implementation of electronic Integrated Management of Childhood Illness (eIMCI) at the primary care level in Burkina Faso: a qualitative study on health worker perception of its medical content, usability and impact on antibiotic prescription and resistance. BMC Public Health. 2019 Apr 29;19(1):449.
- Car J, Carlstedt-Duke J, Tudor Car L, et al. Digital Education in Health Professions: The Need for Overarching Evidence Synthesis. *J Med Internet Res*. 2019;21(2):e12913.
- Carlson AL, Xia K, Azcarate-Peril MA, et al. Infant Gut Microbiome Associated With Cognitive Development. *Biol Psychiatry*. 2018;83(2):148-159.
- Carroll M, Rangaiahagari A, Musabeyezu E, Singer D, Ogbuagu O, Five-year antimicrobial susceptibility trends among bacterial isolates from a tertiary healthcare facility in Kigali, Rwanda. Am J Trop Med Hyg, 2016; 95(6): 1277-1283.
- Committee for Proprietary Medicinal P. Points to consider on switching between superiority and noninferiority. Br J Clin Pharmacol. 2001;52(3):223-8.
- Cordey S, Laubscher F, Hartley M-A, Junier T, Pérez-Rodriguez FJ, Keitel K, Vieille G, Samaka J, Mlaganile T, Kagoro F, Boillat-Blanco N, Mbarack Z, Docquier M, Tapparel C, Kaiser K , D’Acremont V. Detection of Dicistroviruses RNA in Blood of Febrile Tanzanian Children. *Emerg Microbes Infect*. 2019, 8(1):613-623.
- Cordey S, Hartley MA, Keitel K, Laubscher F, Brito F, Junier T, Kagoro F, Samaka J, Masimba J, Said Z, Temba H, Mlaganile T, Docquier M, Fellay J, Kaiser L, D’Acremont V. Detection of novel astroviruses MLB1 and MLB2 in the sera of febrile Tanzanian children. *Emerg Microbes Infect*. 2018; 7(1):27.

- D'Acremont V, Kilowoko M, Kyungu E, Philipina S, Sangu W, Kahama-Maro J, Lengeler C, Cherpillod P, Kaiser L, and Genton B.'Beyond Malaria — Causes of Fever in Outpatient Tanzanian Children', *NEJM* 2014; 370: 809-17.
- De Santis O, Kilowoko M, Kyungu E, Sangu W, Cherpillod P, Kaiser L, Genton B, D'Acremont V. Predictive value of clinical and laboratory features for the main febrile diseases in children living in Tanzania: A prospective observational study. *PLoS One* 2017;12(5):e0173314.
- Erdman LK, D'Acremont V, Hayford K, Rajwans N, Kilowoko M, Kyungu E, Hongoa P, Alamo L, Streiner DL, Genton B, Kain KC. Biomarkers of Host Response Predict Primary End-Point Radiological Pneumonia in Tanzanian Children with Clinical Pneumonia: A Prospective Cohort Study. *PLoS One* 2015; 10(9): e0137592.
- Farnham A, Utzinger J, Kulinkina AV, Winkler MS. Using district health information to monitor sustainable development. Bull World Health Organ. 2020 Jan 1;98(1):69-71.
- Fink G, D'Acremont V, Leslie HH, Cohen J. Antibiotic exposure among children younger than 5 years in low-income and middle-income countries: a cross-sectional study of nationally representative facility-based and household-based surveys. *Lancet Infect Dis.* 2019 Dec 13.
- Frischknecht R., Jungbluth N., Althaus H.-J., Doka G., Heck T., Hellweg S., Hischier R., Nemecek T., Rebitzer G., Spielmann M., Wernet G. Overview and Methodology. ecoinvent report No. 1. Swiss Centre for Life Cycle Inventories, Dübendorf, 2007.
- Gupta N, Hirschhorn L, Rwabukwisi FC, Drobac P, Sayinzoga F, Mugeni C, et al. 2018. Causes of death and predictors of childhood mortality in Rwanda: a matched case-control study using verbal social autopsy. BMC Public Health 18, 1378.
- Hansoti B, Jenson A, Keefe D, De Ramirez SS, Anest T, Twomey M, Lobner K, Kelen G, Wallis L. Reliability and validity of pediatric triage tools evaluated in Low resource settings: a systematic review. BMC Pediatr. 2017 Jan 26;17(1):37.
- Hansoti B, Jenson A, Kironji AG, Katz J, Levin S, Rothman R, Kelen GD, Wallis LA. SCREEN: A simple layperson administered screening algorithm in low resource international settings significantly reduces waiting time for critically ill children in primary healthcare clinics. PLoS One. 2017;12(8):e0183520.
- Hategeka C, Mwai L, Tuyisenge L. Implementing the Emergency Triage, Assessment and Treatment *plus* admission care (ETAT+) clinical practice guidelines to improve quality of hospital care in Rwandan district hospitals: healthcare workers' perspectives on relevance and challenges. *BMC Health Serv Res* 2017;17:256.
- Head SJ, Kaul S, Bogers AJ, Kappetein AP. Non-inferiority study design: lessons to be learned from cardiovascular trials. Eur Heart J. 2012;33(11):1318-24.
- Henninger S. Does the global warming modify the local Rwandan climate? Natural Science 2012;5:124-129.
- Holmes AH, Moore LS, Sundsfjord A, Steinbakk M, Regmi S, Karkey A, Guerin PJ, Piddock LJ. Understanding the mechanisms and drivers of antimicrobial resistance. Lancet. 2016 Jan 9;387(10014):176-87.
- Hopkins H, Cairns ME, Chandler CIR, Leurent B, Ansah EK, Baiden F, Baltzell KA, Björkman A, Burchett HED, Clarke SE, DiLiberto D, Elfving K, Goodman C, Hansen KS, Kachur SP, Lalloo DG, Leslie T, Magnussen P, Jefferies M, Mårtensson A, Mayan I, Mbonye AK, Msellem MI, OE, Owusu-Agyei S, Reyburn H, Rowland MW, Shakely D, Vestergaard LS, Webster J, Wiseman VL, Yeung S, Schellenberg D, Staedke SG, Whitty CJM. Impact of introduction of rapid diagnostic tests for malaria on antibiotic prescribing: analysis of observational and randomised studies in public and private healthcare settings. BMJ 2017;356:j1054.
- International Organization for Standardization (ISO). Environmental management - Life cycle assessment – Principles and framework. ISO 14040: 2006A; Second Edition 2006-06, Geneva.
- International Organization for Standardization (ISO). Environmental management - Life cycle assessment – Requirements and guidelines. ISO 14044: 2006B; First edition 2006-07-01, Geneva.
- Jolliet O, Müller-Wenk R, Bare J, Brent A, Goedkoop M, Heijungs R, Itsubo N, Peña C, Pennington D, Potting J, Rebitzer G, Stewart M, Udo de Haes H and Weidema B. The LCIA Midpoint-damage Framework of the UNEP/SETAC Life Cycle Initiative, Int J LCA 2004: 9 (6) 394 – 404.
- Kahan BC, Morris TP. Reporting and analysis of trials using stratified randomisation in leading medical journals: review and reanalysis. BMJ. 2012;345:e5840.

- Keitel K, Kilowoko M, Kyungu E, Genton B, D'Acremont V. Performance of prediction rules and guidelines in detecting serious bacterial infections among Tanzanian febrile children. *BMC Infect Dis* 2019; 19:769.
- Keitel K, Samaka J, Masimba J, Temba H, Said Z, Kagoro F, Mlaganile T, Sangu W, Genton B, D'Acremont V. Safety and efficacy of C-reactive protein-guided antibiotic use to treat acute respiratory infections in Tanzanian children: a planned subgroup analysis of a randomized, controlled non-inferiority trial evaluating a novel electronic clinical decision algorithm (ePOCT). *Clin Infect Dis.* 2019; 69(11):1926-1934.
- Keitel K, D'Acremont V. Electronic Clinical Decision Algorithms for the integrated primary care management of febrile children in low resource settings - Review of existing tools. *Clin Microbiol Infect*. 2018, 24(8):845-855.
- Keitel K, Kagoro F, Samaka J, Masimba J, Said Z, Temba H, Mlaganile T, Sangu W, Rambaud-Althaus C, Gervaix A, Genton B, D'Acremont V. A novel electronic algorithm using host biomarker point-of-care-tests for the management of febrile illnesses in Tanzanian children (e-POCT): A randomized, controlled non-inferiority trial. *Plos Medicine* 2017;14(10):e1002411.
- Lange S, Mwisongo A, Mæstad O. Why don't clinicians adhere more consistently to guidelines for the Integrated Management of Childhood Illness (IMCI)? Soc Sci Med. 2014;104:56-63.
- Loughman A, Ponsonby AL, O'Hely M, et al. Gut microbiota composition during infancy and subsequent behavioural outcomes. EBioMedicine 2020;52:102640.
- Maternal and Child Survival Program (MCSP). An Alternative to Classroom-Based Health Worker Training in Rwanda: MCSP Rwanda Case Study (2018). https://www.mcsprogram.org/resource/an-alternative-to-classroom-based-health-worker-training-in-rwanda/
- Mghamba, J., Mboera, L., Krekamoo, W., Senkoro, K., Rumisha, S., Shayo, E., & Mmbuji,. Challenges of implementing an integrated disease surveillance and response strategy using the current health management information system in Tanzania. P. J. T. J. o. H. R. 2004; 6(2), 57-63
- Nantanda, Rebecca, James K. Tumwine, Grace Ndeezi, and Marianne S. Ostergaard. 'Asthma and pneumonia among children less than five years with acute respiratory symptoms in Mulago Hospital, Uganda: Evidence of under-diagnosis of asthma', PLoS ONE 2013, 8: 1-9.
- National Institute of Statistics of Rwanda (NISR) [Rwanda], Ministry of Health (MOH) [Rwanda], and ICF International. 2015. Rwanda Demographic and Health Survey 2014-15. Rockville, Maryland, USA: NISR, MOH, and ICF International.
- Nisabwe L, Brice H, Umuhire MC, Gwira O, Harelimana JDD, Nzeyimana Z. Knowledge and attitudes towards antibiotic use and resistance among undergraduate healthcare students at University of Rwanda. J Pharm Policy Practice, 2020; 13(7).
- Ntirenganya C, Manzi O, Muvunyi CM, Ogbuagu O. High prevalence of antimicrobial resistance among common bacterial isolates in a tertiary healthcare facility in Rwanda. Am J Trop Med Hyg, 2015, 92(4): 865-870.
- Piaggio G, Elbourne DR, Pocock SJ, Evans SJ, Altman DG, Group C. Reporting of noninferiority and equivalence randomized trials: extension of the CONSORT 2010 statement. JAMA. 2012;308(24):2594-604.
- Pocock SJ, Assmann SE, Enos LE, Kasten LE. Subgroup analysis, covariate adjustment and baseline comparisons in clinical trial reporting: current practice and problems. Stat Med. 2002;21(19):2917-30.
- Rambaud-Althaus, Clotilde, Fabrice Althaus, Blaise Genton, and Valérie D'Acremont. 'Clinical features for diagnosis of pneumonia in children younger than 5 years: a systematic review and meta-analysis.', The Lancet. Infectious diseases, 2015; 15: 439-50.
- Rambaud-Althaus C, Shao S, Samaka S, Swai N, Perri S, Kahama-Maro J, Mitchell M, D'Acremont V, and Genton B. 'Performance of Health Workers Using an Electronic Algorithm for the Management of Childhood Illness in Tanzania: A Pilot Implementation Study.' *Am J Trop Med Hyg* 2017; 96: 249-57.
- Rowe, AK et al (2009). Review of strategies to improve health care provider performance. In: Peters DH ed. Improving Health Service Delivery in Developing Countries: from Evidence to Action. Washington, DC: World Bank, 101-126.
- Ryan, S. J., McNally, A., Johnson, L. R., Mordecai, E. A., Ben-Horin, T., Paaijmans, K., Lafferty, K. Mapping Physiological Suitability Limits for Malaria in Africa Under Climate Change. Vector-Borne And Zoonotic Diseases. 2015; 15 (12): 718-725

- Schulz KF, Altman DG, Moher D, Group C. CONSORT. Statement: updated guidelines for reporting parallel group randomised trials. Trials. 2010;11:32.
- Shao AF, Rambaud-Althaus C, Samaka J, Faustine AF, Perri-Moore S, Swai N, Kahama-Maro J, Mitchell M, Genton B, D'Acremont V. New Algorithm for Managing Childhood Illness Using Mobile Technology (ALMANACH): A Controlled Non-Inferiority Study on Clinical Outcome and Antibiotic Use in Tanzania. *PLoS One* 2015A; 10(7): e0132316.
- Shao A, Rambaud-Althaus C, Swai N, Kahama-Maro J, Genton B, D'Acremont V, and Pfeiffer C. 'Can smartphones and tablets improve the management of childhood illness in Tanzania? A qualitative study from a primary health care worker's perspective.', BMC Health Services Research 2015B; 15: 135.
- Sutherland T, Mpirimbanyi C, Nziyomaze E, Niyomugabo JP, Niyonsenga Z, Muvunyi CM, Mueller A, Bebell LM, Nkubana T, Musoni E, Talmor D, Rickard J, Riviello ED. Widespread antimicrobial resistance among bacterial infections in a Rwandan referral hospital. PLoS ONE 14(8): e0221121.
- USAID. The Demographic and Health Surveys Program. https://dhsprogram.com/What-We-Do/Survey-Types/SPA-Questionnaires.cfm
- Walker, P. G., M. T. White, J. T. Griffin, A. Reynolds, N. M. Ferguson, and A. C. Ghani. 'Malaria morbidity and mortality in Ebola-affected countries caused by decreased health-care capacity, and the potential effect of mitigation strategies: a modelling analysis', Lancet Infect Dis 2015; 15: 825-32.
- World bank 2018. Mortality rate, under-5 (per 1,000 live births) - Sub-Saharan Africa, World. https://data.worldbank.org/indicator/SH.DYN.MORT?locations=ZG-1W
- World Health Organization 2006. 'Improving health worker performance:in search of promising practice'. https://www.who.int/hrh/resources/improving_hw_performance.pdf
- World Health Organization 2011. Interagency 'Universal access to malaria diagnostic testing: an operational manual'. http://www.who.int/malaria/publications/atoz/9789241502092/en/index.html
- World Health Organization 2013. Howie S. Blood sample volumes in child health research: review of safe limits. http://www.who.int/bulletin/volumes/89/1/10-080010/en/#
- World Health Organization 2014A. 'IMCI Chart Booklet'. https://www.who.int/maternal_child_adolescent/documents/IMCI_chartbooklet/en/
- World Health Organization 2014B. "Ground zero in Guinea: the Ebola outbreak smoulders – undetected – for more than 3 months". http://www.who.int/csr/disease/ebola/ebola-6-months/guinea/en/
- World Health Organization 2018. Climate change and health: Key facts. Geneva, Feb 1. https://www.who.int/news-room/fact-sheets/detail/climate-change-and-health
- Yukich J, D'Acremont V, Kahama J, Swai N, Lengeler C. Cost savings with rapid diagnostic tests for malaria in low-transmission areas: evidence from Dar es Salaam, Tanzania. *Am J Trop Med Hyg.* 2010; 83(1): 61-8.

16 APPENDICES

Appendix A: Case Report Forms

- A1 – Information collected electronically during clinical consultations for the main study
- A2 – Information collected via a phone call or SMS or home visit for the main study

Appendix B: Mixed-methods operational research tools

- B1 – Facility Assessment (adapted from SPA inventory tool)
- B2 – Quality of Care Consultation Observation Checklist (adapted from SPA sick child tool)
- B3 – Quality of Care Client Exit Interview (adapted from SPA sick child tool)
- B4 – Qualitative In-depth Interview: Health Worker
- B5 – Focus Group Discussion Guide: Health Worker
- B6 – Quantitative Survey: Health Worker
- B7 – Qualitative In-depth Interview: Caregiver
- B8 – Focus Group Discussion Guide: Caregiver
- B9 – Quantitative Survey: Caregiver
- B10 – Qualitative In-depth Interview: DHMT

Appendix C: Informed Consents

- C1 – Participant information sheet and informed consent form for the main study
- C2 – Participant information sheet and informed consent form for the mixed-methods operational research studies
- C3 – Consent form for photo/video

Appendix D: CVs of Team Members

Appendix E: Example content of the algorithms

- E1 – Visual representation (diagrams) of the Tanzania algorithms
- E2 – Modifications made based on the Tanzanian clinical expert review
