# Supplementary material for: Effectiveness of a digital clinical decision support algorithm for guiding antibiotic prescribing in pediatric outpatient care in Rwanda: A pragmatic cluster non-randomized controlled trial
Source: PLoS Med. 2026 Feb 26;23(2):e1004692. doi: 10.1371/journal.pmed.1004692 (PMC12944774; doi:10.1371/journal.pmed.1004692)
Supplement: S2 Annex — (PDF) [file pmed.1004692.s002.pdf]

# DYNAMIC Rwanda Phase I Statistical Analysis Plan

| | | | |
|---|---|---|---|
| **Trial registration number** | NCT05108831 | | |
| **SAP version number** | Version 1.0 | **Date** | 10-Sep-2021 |
| **Protocol version** | This document has been written based on information contained in the study protocol version 2.1, dated 10 September 2021 | | |

| **Authors** | **Position** | **Signature** | **Date** |
|---|---|---|---|
| Alexandra Kulinkina | Project Coordinator | | |
| Valérie D'Acremont | Principal Investigator | | |
| Ludovico Cobuccio | Research Physician | | |
| **Approved by** | | | |
| Marie-Annick Le Pogam | Statistical Advisor | | |
| Rainer Tan | External Collaborator | | |

**Contributing collaborators/authors**

| **Name** | **Position** |
|---|---|
| Gilbert Rukundo | Data Manager |
| Mary-Anne Hartley | Scientific project leader |

**Revision history**

| **Protocol version** | **SAP version no.** | **Section number changed** | **Description of and reason for change** | **Date changed** |
|---|---|---|---|---|
| | | | | |
| | | | | |
| | | | | |
| | | | | |

# Contents

Abbreviations and glossary of terms .......... 3
1. Introduction .......... 4
1.1 Hypothesis .......... 4
1.2 Objective .......... 4
2. Study outcomes .......... 4
2.1 Outcome definitions .......... 4
2.1.1 Primary outcome .......... 4
2.1.2 Secondary outcomes .......... 4
3. Methods .......... 6
3.1 Study design .......... 6
3.2 Allocation of health facilities to study arms .......... 7
3.3 Sample size .......... 7
3.4 Statistical interim analyses .......... 8
3.5 Timing of final analysis .......... 8
4. Statistical principles .......... 8
4.1 Adherence and protocol deviations .......... 8
4.2 Analysis population definitions .......... 8
5. Study population .......... 9
5.1 Screening data .......... 9
5.2 Eligibility .......... 9
5.2.1 Health facility (cluster) eligibility .......... 9
5.2.2 Study population eligibility .......... 9
5.3 Recruitment .......... 9
5.4 Baseline characteristics .......... 9
6. Main analyses .......... 10
6.1 Analysis methods .......... 10
6.2 Exploratory analyses .......... 11
References .......... 12

# Abbreviations and glossary of terms

| | |
|---|---|
| CDSA | Clinical Decision Support Algorithm |
| CI | Confidence interval |
| CRP | C-reactive protein |
| ePOCT+ | Name of CDSA (not an abbreviation) |
| HIV | Human Immunodeficiency Virus |
| HF | Health facility |
| HW | Health worker |
| IQR | Interquartile range |
| IMCI | Integrated management of childhood illness |
| NCD | Non-communicable disease |
| PCs | Personal Computers |
| PHC | Primary health care |
| POC | Point-of-care |
| RDT | Rapid Diagnostic Test |
| RW | Rwanda |
| Swiss TPH | Swiss Tropical and Public Health Institute |
| TB | Tuberculosis |
| Unisanté | Centre for Primary Care and Public Health (University of Lausanne) |
| WHO | World Health Organization |

# 1. Introduction

## 1.1 Hypothesis

In routine conditions, health workers (HWs) provided with an electronic clinical decision support algorithm (CDSA) on tablets or PCs can treat sick children attending primary health care facilities in Rwanda with appropriate antimicrobial prescriptions. More specifically, we hypothesize that with the use of CDSA called electronic point of care tests plus (ePOCT+) for case management of sick children in primary health care facilities, compared to routine care, less antibiotics will be prescribed at the initial consultation.

## 1.2 Objective

The overall objective of the DYNAMIC project is to improve the **integrated management of children with an acute illness** through the provision of an electronic CDSA (ePOCT+) to clinicians working at primary care level. The specific research objective of the Phase 1 of the DYNAMIC project is to determine if the use of ePOCT+ for case management of sick children in primary care, compared to routine care, results in less antibiotic prescriptions at initial consultation (primary outcome).

# 2. Study outcomes

## 2.1 Outcome definitions

### 2.1.1 Primary outcome

*Percentage of children prescribed an antibiotic at initial consultation in the intervention group (ePOCT+) as compared to the control group (routine care)*

Outcome measure: Prescription of oral or parenteral antibiotic at initial consultation, as reported by the HW.

Timing and method of measurement: Documented by the HW at the end of the initial consultation where the subject was enrolled (day 0).

Analysis type: Superiority

### 2.1.2 Secondary outcomes

All secondary outcomes are compared between the intervention (ePOCT+) and control (routine care) arms.

*Percentage of children cured at day 7 in the intervention group (ePOCT+) as compared to the control group (routine care)*

Outcome measure: The child is defined as being cured at day 7 if the caregiver says that the child is cured or has improved since the initial consultation. Non-referred secondary hospitalizations (if caregiver says that child was hospitalized between day 0 and day 7 but the electronic clinical data does not indicate a referral for hospitalization) will however be considered as clinical failures even if the child is already cured at day 7.

Timing and method of measurement: Assessment of subjective clinical cure by the caregiver, and history of non-referred hospitalization will be assessed by telephone or home visit follow-up 7 days (range 6-14 days) after enrollment of the subject. The day of enrollment of the subject is considered as day 0.

Analysis type: Superiority

*Secondary consultations:*

- % of children with one or more unscheduled re-attendance visits at any health facility by day 7

Timing and method of measurement: Telephone or home visit follow-up 7 days (range 6-14 days) after enrollment of the subject. The day of enrollment of the subject is considered as day 0.

*Severe outcome by day 7:*

- % of children with non-referred secondary hospitalization by day 7
- % of children who have died by day 7

Timing and method of measurement: Death and non-referred secondary hospitalization will be assessed by telephone or home visit follow-up 7 days (range 6-14 days) after enrollment of the subject. The day of enrollment of the subject is considered as day 0.

*Primary referrals:*

- % of children referred to hospital or inpatient ward at a health centre at initial consultation

Timing and method of measurement: Documented by the HW at the end of the initial consultation in the eCRF (control arm) or in ePOCT+ (intervention arm) when the subject was enrolled (day 0)

*Appropriate case management for malaria at initial consultation*

- % of febrile children tested for malaria by RDT and/or microscopy at day 0
- % of malaria positive children (positive RDT result OR positive microscopy result) prescribed an antimalarial at day 0
- % of malaria negative children prescribed an antimalarial at day 0
- % of untested children prescribed an antimalarial at day 0

Timing and method of measurement: Documented by the HW at the end of the initial consultation where the subject was enrolled (day 0)

**Definitions:**

*Clinical cure is a positive answer to the question "Is the child cured?" or if not cured "Has the child improved?" asked to caregiver through a phone call at day 7. Non-referred secondary hospitalizations (see definition below) will be considered as clinical failures even if the child is already cured at day 7.*
*Clinical failure is a negative answer to the question "Is the child cured?" and "Has the child improved" asked to the caregiver through a phone call at day 7. Non-referred secondary hospitalization (see definition below) will be considered as a clinical failure even if the child is already cured at day 7.*
*Initial consultation is the first visit of a sick child for an acute problem at a HF participating in the study (and thus registered electronically); timeframe from completion of the initial visit up to midnight of the same day. The initial consultation is considered as day 0.*
*Re-attendance visit is a consultation (not necessarily at a HF participating in the study) taking place from the day after initial consultation up to day 14 included. A re-attendance visit can be scheduled (proposed by the HW on a certain day) or unscheduled (upon decision by caregivers).*
*Primary referral is a decision of referring or admitting the child at a hospital or health center for at least one night, taken by the HW at the initial consultation.*
*Secondary referral is a decision of referring or admitting the child at a hospital or health center taken by the HW during a re-attendance visit taking place from the day after initial consultation up to day 7 included.*
*Primary hospitalization is an admission to a hospital or health center ward for at least one night taking place the same day as the initial consultation*
*Secondary hospitalization is an admission to a hospital or health center ward for at least one night taking place from the day after initial consultation up to the phone call at day 7 included. A non-referred secondary hospitalization is a direct visit to hospital without a re-attendance visit the same day at a HF participating in the study. Non-referred secondary hospitalization will be determined if the caregiver reports during the day 7 phone follow-up that the child was hospitalized, even if there was no documentation of a referral from initial and re-attendance visits at participating health facilities.*

*Antibiotic prescription* is any oral, intramuscular or intravenous (but not topical) antibiotic prescribed by a HCW during the initial consultation or a re-attendance visit.
*Antimalarial prescription* is any oral, rectal, intramuscular or intravenous antimalarial prescribed by a HCW during the initial consultation or a re-attendance visit.
*Febrile child* is a child with a history of fever (measured or suspected fever in the past 48 hours) or a high temperature.
*Malaria testing* is a malaria RDT or microscopy ordered by a HCW during an outpatient visit.

# 3. Methods

## 3.1 Study design

The DYNAMIC RW Phase 1 study is a pragmatic, open-label two-arm parallel-group cluster non-randomized controlled superiority trial implemented in a sequential manner. There are three distinct implementation blocks of half intervention and half control health facilities that will be enrolled in the study at three sequential time points. The intervention consists of the provision to health workers of the ePOCT+ clinical decision support algorithm (CDSA), point-of-care tests proposed by ePOCT+ that are not part of routine care (pulse oximeter, CRP rapid test, additional hemoglobin cuvettes), complementary training on the tool, regular monitoring and mentorship/supervision visits by the study team and/or the District Health Management Team (DHMT). Mentorship and supervision will be enabled by a complementary dashboard (medAL-monitor), used to visualize and monitor study-related indicators.

Since the intervention takes place at the health provider level and their practices are influenced by the context of the health facility they are working in, randomization at health facility level rather than at HCW level or at individual patient level was chosen. The randomization of clusters (i.e. health facilities) will be performed on a 1:1 ratio. Thus, ePOCT+ will be provided to health workers (HWs) of 16 of the 32 selected primary health care facilities (intervention arm) in Rusizi and Nyamasheke districts, while the other 16 health facilities (HFs) will serve as controls (control arm). Sick children aged 1 day to 14 years (inclusive) will be managed in accordance with the study arm to which the HF they attend has been assigned. The comparator for Phase 1 will be routine care as currently implemented in Rwanda supplemented with equivalent clinical training given to the intervention arm. To assess the primary and secondary outcomes, final decision on referral, laboratory test results, diagnoses and treatments at initial consultation will be recorded by HCWs electronically in both arms (in the intervention arm integrated into the CDSA; in the control arm via a simple eCRF without any decision support); children will also be followed-up by a phone call or home visit to their caregiver at day 7 (range 6-14) to determine their clinical status. The study is considered as pragmatic since there are no major exclusion criteria to the population of interest, and the setting and organization of the health facilities remains mostly the same. The trial design is summarized below. Furthermore, in line with the pragmatic nature of the study, the design is adaptive, in that changes in the implementation across the three parallel cluster studies may be made based on monitoring data and feedback from the health facilities. These implementation changes (excluding significant adaptations to algorithm content) will be thoroughly documented and accounted for in comparisons across the three cluster studies.

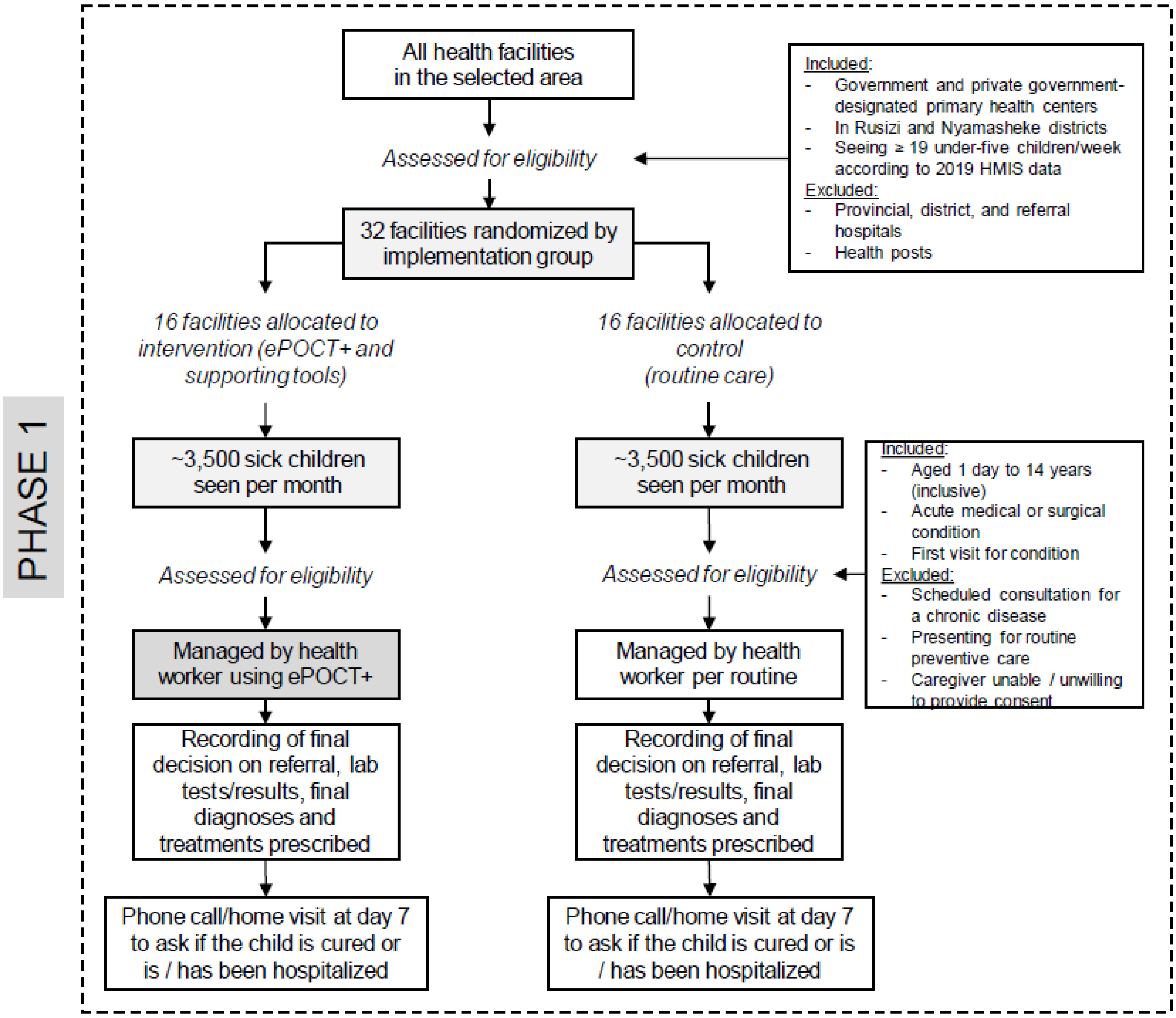


## 3.2 Allocation of health facilities to study arms

The 32 health facilities to be included in the study will be selected from all eligible health facilities in the participating districts (Rusizi and Nyamasheke). Eligible health facilities include public health centers with at least 19 consultations with children between 2 months and 5 years of age per week. Cluster inclusion and exclusion criteria will first be applied to the full list of health facilities in the participating districts prior to selection of the 32 health facilities. Health facilities (or clusters) will be allocated (1:1), to using ePOCT+ (intervention) or routine care (control group). Due to the pragmatic nature of the study, randomization is not possible. Health facilities will be allocated first to one of the three implementation blocks using geographical considerations, and then split into study arms, also using geographical considerations. Similar sample size by study arm and by implementation group will be maintained as much as possible.

## 3.3 Sample size

The primary analysis will evaluate whether the use of ePOCT+ for case management of sick children in PHC facilities results in decreased antibiotic prescriptions (superiority analysis) at the initial consultation. Superiority in antibiotic prescription is defined as a relative decrease of ≥25% in the proportion of children prescribed an antibiotic. We estimated a cluster size of 660 patients (monthly utilization of 220 – accounting for up to 30% non-adherence and/or loss to follow-up from 315 – over 3 months) and an intraclass correlation coefficient of 0.025. Non-adherence here is defined as HWs not using the application for all eligible patients that come to the HF for various reasons. To have 80% power to detect a 25% reduction in antibiotic prescription (from 35% to 26%), for a two-sided hypothesis test at alpha of

0.05, we would require a minimum of 11 clusters per arm. With 16 clusters per arm, over 3 months, we expect a total of 10,500 children in each study arm. Considering the pragmatic/adaptive nature of the study design, if a higher antibiotic prescription in the control arm is encountered (e.g. 45% or higher), each cluster study of 5-6 health facilities per study arm will be sufficiently powered to show a statistically significant reduction in the primary outcome. Sample size calculations were performed in R statistical software (version 4.0.3) using the CRTSize package (Rotondi, 2015).

## 3.4 Statistical interim analyses

Interim analyses for the primary endpoints will be performed after each of the cluster studies. As previously mentioned, minor implementation changes may be made throughout the study to adapt to issues discovered from the monitoring data and feedback from the field at the time of the interim analyses. The Principal Investigators may decide to terminate the study prematurely according to certain circumstances, such as ethical concerns that cannot be resolved, in case of safety of the participants being compromised (e.g. if the benefits no longer outweigh the risks), alterations in accepted clinical practice that make the continuation of the study unwise, or evidence of harm of the intervention. In case of premature study termination, the Rwanda National Ethics Committee will be notified as per local guidelines.

## 3.5 Timing of final analysis

The final analysis will be performed after all enrolled study subjects have the day 7 outcome measured.

# 4. Statistical principles

## 4.1 Adherence and protocol deviations

Adherence to the study protocol is assessed based on the percent of patients enrolled in the study for which the clinician entered the final treatment (or confirmed the absence of treatment).

Adherence is defined as: % adherence to the study protocol = (number of children for whom the final treatment was inputted / number of children enrolled in the study) * 100%

Descriptive statistics on the adherence (N, mean, SD, median, minimum, maximum) will be provided by study arm (intervention and control).

## 4.2 Analysis population definitions

Intention-to-treat population: This population includes all patients enrolled in the study, regardless of whether the HW used ePOCT+ (intervention arm), or entered the final treatment data in the eCRF (both arms).

Per-protocol population: This population only includes patients enrolled in the study for whom the clinician confirmed the final treatment (or absence of treatment) / referral status within the ePOCT+ tool (intervention arm) or in the eCRF (control arm).

Complete case set: All enrolled subjects (intervention or control) for whom day 7 clinical cure data was collected (i.e. excluding lost to follow-up patients).

Complete consultation set: All enrolled subjects (intervention or control) for whom at least information on treatments were collected at day 0.

# 5. Study population

## 5.1 Screening data
All patients presenting to the outpatient department of participating health facilities will be screened for eligibility. At the screening stage, the age of the patient and reason for coming to the health facility (acute vs. routine or chronic care visit) will be documented. The following summary data for the facility will be presented: the number of days during which screening has taken place, the number of patients recruited, the average number of patients recruited per day, the number of screened patients not recruited, and the reason for non-recruitment (e.g. non-eligibility vs. declined enrollment).

## 5.2 Eligibility
### 5.2.1 Health facility (cluster) eligibility
Heath facility eligibility for the study is based on the health facility meeting all inclusion criteria and none of the exclusion criteria:

**Inclusion Criteria**:
- Government and NGO-supported government-designated primary health centers
- Located in Rusizi and Nyamasheke districts of the Western Province of Rwanda
- Seeing at least 19 children aged less than 5 years per week for IMCI services in 2019 (to facilitate participant recruitment)*

*Based on estimates from extrapolated data from HMIS.

**Exclusion Criteria**:
- Primary health posts
- Secondary and Tertiary HFs (district, regional, zonal and specialized hospitals)

### 5.2.2 Study population eligibility
A patient is eligible for the study if they meet all inclusion criteria and none of the exclusion criteria:

**Inclusion Criteria:**
- Aged 1 day (24 hours) to 14 years (inclusive)
- Presenting for an acute medical or surgical condition

**Exclusion Criteria:**
- Presenting for scheduled consultation for a chronic disease (e.g. HIV, TB, NCD, malnutrition)
- Presenting for routine preventive care (e.g. growth monitoring, vitamin supplementation, deworming, vaccination).
- Caregiver unavailable, unable or unwilling to provide informed consent (except for older children who can provide assent with an adult witness during the consenting process).

## 5.3 Recruitment
A CONSORT flow diagram will be used to summarize the number of patients throughout the study process.

## 5.4 Baseline characteristics
The baseline cluster (health facility) characteristics that will be summarized by study arm include:
- Number of health facilities by district
- Average number of patients seen per month by health facility
- Availability and readiness of basic health care services score based on the Rwandan Service Provision Assessment (median and IQR)

The baseline patient characteristics that will be summarized by study arm, and for the overall study include:

- Demographics: sex (number and percentage); age (median and IQR), age groups 0-2 months, 2 months - 5 years, 5-14 years (number and percentage)
- Medical history: Main reasons for consultation, history of fever (number and percentage)

Given the pragmatic nature of the study, and so as not to influence the normal management of patients and their outcome in the control arm, clinical signs and comorbidities will not be systematically collected in the control and intervention arm.

Baseline data will be compared across the study arms to confirm no bias due to lack of true randomization.

# 6. Main analyses

## 6.1 Analysis methods

Analyses will follow CONSORT guidelines (Campbell, Piaggio, Elbourne, & Altman, 2012; Piaggio et al., 2012; Schulz, Altman, & Moher, 2010; Zwarenstein et al., 2008). Outcomes will be described by arm using summary statistics. Baseline characteristics will be compared by study arm and by cluster study to ensure that the pragmatic allocation of health facilities and sequential rollout of the study did not introduce significant bias. Baseline characteristics which will differ between treatment arms within and over all clusters will be described using summary statistics.
Since the documentation of final treatment in both arms is part of the per protocol definition, only the PP population can be used for primary outcome analysis. The primary outcome will be evaluated using a random effects logistic regression model with the binary outcome variable of antibiotic prescription, with study arm and covariates at patient-level (e.g. age, sex) as fixed effects, and cluster (health facility) and patient included as random effects. Binary secondary outcomes will be evaluated in the same way. All estimates will be reported as odds ratios (ORs) with two-sided 95% confidence intervals (CI). The study will be deemed a success if antibiotic prescription at the initial consultation is 25% less in the ePOCT+ arm compared to the control arm, although we expect a greater reduction based on limited pilot data.

Effect modification of the primary outcome of antibiotic prescription will be performed in groups of patients where the highest reduction in antibiotic prescription is expected, and areas of extension of the content of the algorithm compared to the version tested in the previous ePOCT study. Effect modification by sex, age group (young infants ages 1 to 59 days, children aged 2 months to 5 years, and children aged 5 to 14 years), respiratory symptoms, fever without clinical source, gastrointestinal complaints (vomiting and diarrhea), skin problems, ear, nose and throat problems, will be assessed by incorporating an interaction term between the treatment arm and the respective variable, acknowledging that power will be low. Furthermore in order to adjust for potential imbalances in baseline characteristics between study groups, relevant baseline characteristics will be included as fixed effect variables in the final model. Baseline characteristic imbalance will be determined by statistical method (i.e., comparisons of treatment arms over all clusters and within clusters) and by consensus among the study analysis team.

The number of children lost to follow up may be quite high due to the difficult nature of phone follow-up at day 7, as found in previous studies with day 7 phone call lost to follow up rates of 13-25% (Christie et al., 2018; Hannigan, Chisale, Drew, Watson, & Gallagher, 2019; Nguhuni et al., 2017). Comparatively, the proportion not cured at day 7 is expected to be quite low (3%). The use of a complete case analysis was therefore selected for the secondary outcome of day 7 clinical cure, as it would be the most conservative analysis. If we were to use the "enrolled set" and considered all day 7 lost to follow up as not being cured, this would dilute the difference between arms. Not considering lost to follow up cases as failures is also the approach used for similar studies with high number of LTF

and low proportion of outcomes (Källander et al., 2018). We may adjust the final model for further baseline variables (not already included as fixed effect variables) which are associated with missing outcome data (analogous to performing multiple imputation in the case of a single endpoint). As with the other fixed effect variables, baseline characteristic imbalances among patients lost to follow up will be determined by consensus among the study analysis team.

## 6.2 Exploratory analyses

Unless specifically described, the following analyses will be analyzed similarly to the binary secondary outcomes, using a random effects logistic regression model with the cluster (health facility) and patient included as a random effect.

- % of children hospitalized among those referred for hospitalization during an initial consultation

Timing and method of measurement: Hospitalization determined by day 7 (6-14) phone call or home visit. Referral for hospitalization as determined by eCRF during the initial consultation.

- % of children referred for malnutrition management during the initial consultation

Timing and method of measurement: Referred for malnutrition as documented in eCRF during the initial consultation.

- % of children referred for or performed at the health facility tuberculosis screening during the initial consultation

Timing and method of measurement: As documented in the eCRF during the initial consultation.

- % of children tested for HIV during the initial consultation:

Timing and method of measurement: As documented in the eCRF during the initial consultation.

- % of children tested for any laboratory test during the initial consultation:

Timing and method of measurement: As documented in the eCRF during the initial consultation

- % of clinicians accepting the ePOCT+ testing, treatment, diagnosis, and management recommendations during the initial consultation

Timing and method of measurement: As documented in the eCRF of the intervention arm (ePOCT+) during the initial consultation

- % of children/caretakers seeking additional medications after the initial consultation

Timing and method of measurement: Asking caretakers if they procured additional medications for their child after the initial consultation by phone call or home visit at day 7 (6-14)

- Change in % of children with antibiotic prescribed over time / across three cluster studies

Timing and method of measurement: Antibiotic prescription as documented in the eCRF (control arm) / ePOCT+ tool (intervention) during the initial visit (day 0).

Analysis: Compare change in percentage of prescription from month to month, and across the three cluster studies for the intervention arm and the control arm, using the control arm as comparison to correct for seasonality, and presenting implementation team mentorship visits and combined implementation team and DHMT mentorship visits to contextualize changes. Independent sample t-test will be used to compare change in antibiotic prescription between intervention and control arms.

- Change in % of children with basic anthropometrics and clinical signs assessed over time / across three cluster studies (intervention arm only)

Timing and method of measurement: Measurement and documentation of individual basic measurements (height, MUAC, Temperature), and clinical signs (respiratory rate) as documented within the ePOCt+ tool during the initial assessment (Day 0).

Analysis: Descriptive statistics presenting change in documentation of basic measurements and clinical signs from month to month, and across three cluster studies. Dates of implementation team mentorship visits and combined/supervised implementation team and DHMT mentorship visits and any changes in

implementation strategy will also be presented to contextualize the changes. Subgroup analyses may be conducted for each intervention health facility.

- Change in % of cases managed using ePOCT+ over time / across three cluster studies (intervention arm only):

Timing and method of measurement: Uptake of ePOCT+ is defined as the use of the ePOCT+ tool to guide the clinical consultation including the decision on what treatment to prescribe during the initial consultation (Day 0).

Analysis: Descriptive statistics to present change in the proportion of HWs' uptake from month to month, and across the three cluster studies will be presented. Dates of implementation team mentorship visits and combined/supervised implementation team and DHMT mentorship visits and any changes in implementation strategy will also be presented to contextualize the changes. Subgroup analyses may be conducted for each intervention health facility.

- Prognostic value of individual or combined predictors during the initial consultation to predict clinical failure/cure, hospitalization for more than one night and mortality (intervention arm)

Timing and method of measurement: Individual or combined predictors as documented in the eCRF during the initial consultation in the intervention arm. Clinical failure, hospitalization more than one night, and mortality assessed during the phone calls or home visit at day 7.

Method of analysis: ORs and 95% confidence intervals will be calculated using Pearson's chi-squared test or Fisher's exact test (two-tailed) for binary individual predictors. Initial selection of individual predictors for the bivariate models will be determined by a group of clinical experts, with consideration of the algorithmic logic of the intervention tool to avoid circularity, and evidence on predictors already found to be prognostic. Multivariable ordinal logistic regression with lasso penalty will be used for covariate selection of the final model. Validity tests will be calculated including sensitivity, specificity, positive likelihood ratio, negative likelihood ratio, and area under the receiver operating characteristic. The dataset will be divided into a portion used for the derivation, and another portion for validation.

# References


Campbell, M. K., Piaggio, G., Elbourne, D. R., & Altman, D. G. (2012). Consort 2010 statement: extension to cluster randomised trials. *BMJ : British Medical Journal, 345*, e5661. doi:10.1136/bmj.e5661

Christie, S. A., Mbianyor, M. A., Chichom-Mefire, A., Nana, T., Dicker, R. A., & Juillard, C. (2018). Feasibility of a Cellular Telephone Follow-Up Program after Injury in Sub-Saharan Africa. *Journal of the American College of Surgeons, 227*(4), S129-S130. doi:10.1016/j.jamcollsurg.2018.07.272

Hannigan, A., Chisale, M., Drew, R., Watson, C., & Gallagher, J. (2019). GP133 Mobile phones for follow up in paediatric clinical studies in africa. *104*(Suppl 3), A84-A84. doi:10.1136/archdischild-2019-epa.197 %J Archives of Disease in Childhood

Kahan, B. C., & Morris, T. P. (2012). Reporting and analysis of trials using stratified randomisation in leading medical journals: review and reanalysis. *BMJ (Clinical research ed.), 345*, e5840-e5840. doi:10.1136/bmj.e5840

Källander, K., Alfvén, T., Funk, T., Abebe, A., Hailemariam, A., Getachew, D., . . . Gutman, J. R. (2018). Universal versus conditional day 3 follow-up for children with non-severe unclassified fever at the community level in Ethiopia: A cluster-randomised non-inferiority trial. *PLoS Med, 15*(4), e1002553-e1002553. doi:10.1371/journal.pmed.1002553

MoHCDGEC-HMIS Unit. 2019. Retrieved from http://dhis.moh.go.tz/dhis-web-pivot/app/index.html]

Nguhuni, B., De Nardo, P., Gentilotti, E., Chaula, Z., Damian, C., Mencarini, P., . . . Ippolito, G. (2017). Reliability and validity of using telephone calls for post-discharge surveillance of surgical site infection following caesarean section at a tertiary hospital in Tanzania. *Antimicrobial Resistance & Infection Control, 6*(1), 43. doi:10.1186/s13756-017-0205-0

Piaggio, G., Elbourne, D. R., Pocock, S. J., Evans, S. J. W., Altman, D. G., & Consort Group, f. t. (2012). Reporting of Noninferiority and Equivalence Randomized Trials: Extension of the CONSORT 2010 Statement. *JAMA, 308*(24), 2594-2604. doi:10.1001/jama.2012.87802

Rotondi, M. (2015). Package 'CRTSize' https://cran.r-project.org/web/packages/CRTSize/CRTSize.pdf
Schulz, K. F., Altman, D. G., & Moher, D. (2010). CONSORT 2010 Statement: updated guidelines for reporting parallel group randomised trials. *BMJ, 340*, c332. doi:10.1136/bmj.c332
Zwarenstein, M., Treweek, S., Gagnier, J. J., Altman, D. G., Tunis, S., Haynes, B., . . . Moher, D. (2008). Improving the reporting of pragmatic trials: an extension of the CONSORT statement. *BMJ, 337*, a2390. doi:10.1136/bmj.a2390
