# Supplementary material for: Effectiveness of a digital clinical decision support algorithm for guiding antibiotic prescribing in pediatric outpatient care in Rwanda: A pragmatic cluster non-randomized controlled trial
Source: PLoS Med. 2026 Feb 26;23(2):e1004692. doi: 10.1371/journal.pmed.1004692 (PMC12944774; doi:10.1371/journal.pmed.1004692)

S1 Figure: Longitudinal plots of study variables.

Enrollment, uptake of ePOCT+, follow-up, and clinical failure are shown as percentages. Purple line indicates group A (original intervention) and green line indicates group B (original control). Vertical line indicates the time of cross-over in each block of health centers.

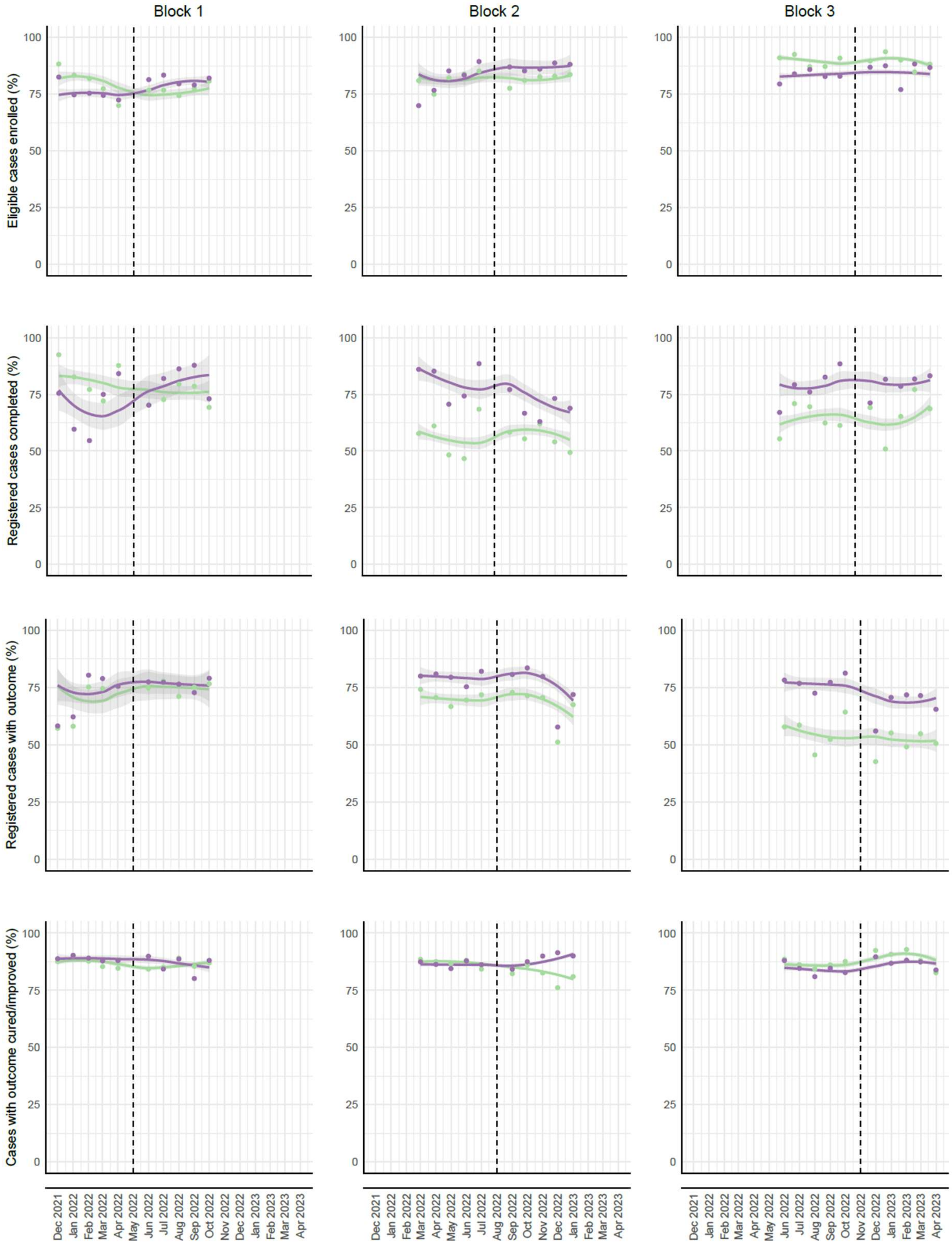

Supplement: S1 Fig — (PDF) [file pmed.1004692.s004.pdf]
