# Supplementary material for: Effectiveness of a digital clinical decision support algorithm for guiding antibiotic prescribing in pediatric outpatient care in Rwanda: A pragmatic cluster non-randomized controlled trial
Source: PLoS Med. 2026 Feb 26;23(2):e1004692. doi: 10.1371/journal.pmed.1004692 (PMC12944774; doi:10.1371/journal.pmed.1004692)
Supplement: S2 Fig — (PDF) [file pmed.1004692.s005.pdf]

S2 Figure: Distribution of clinical outcome across exploratory variables.

Four variables are shown: phone owner, days to outcome, follow-up type and caller identity. Green = clinical cure; purple = clinical failure. The second and fourth panels justify adjusting day 7 outcome models for days to outcome (clinical failures reduced with longer duration) and caller identity (some callers had markedly higher clinical failure rates than others). The third panel justifies excluding data reported by community health workers from home visits, as compared to phone calls; the two modes of follow-up had markedly different clinical failure rates.

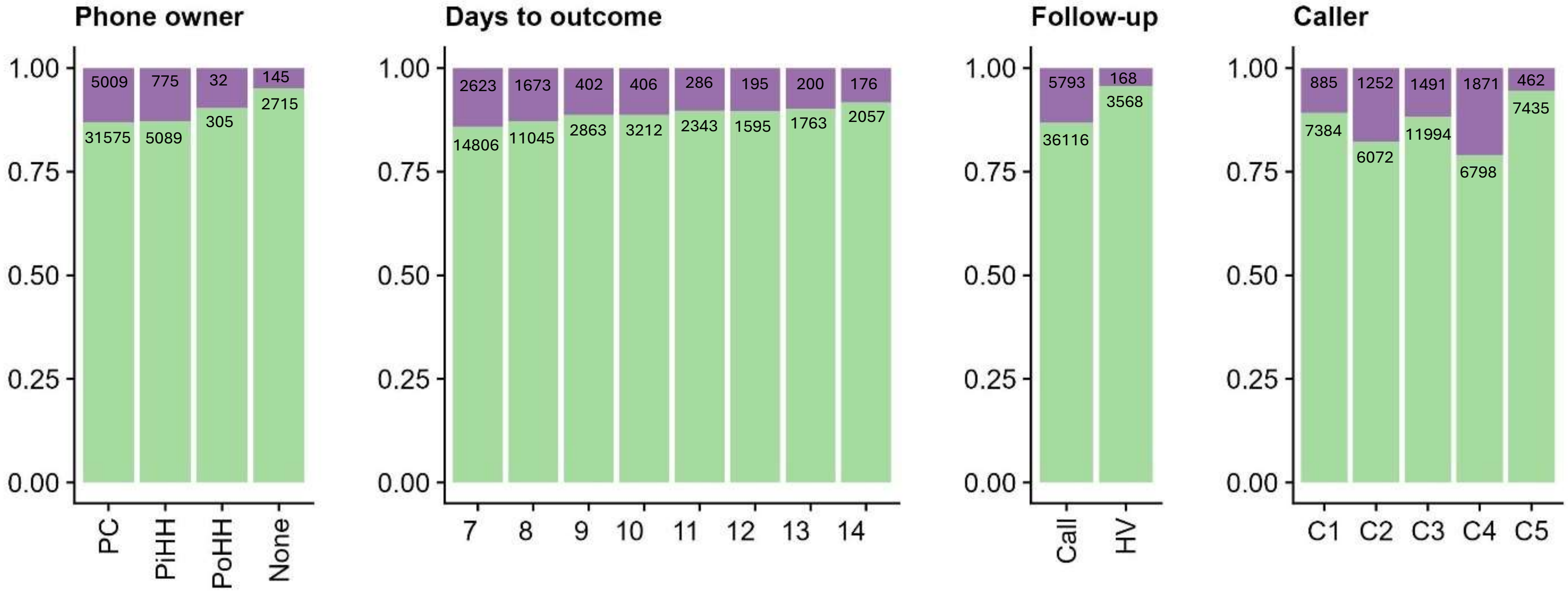

PC = primary caregiver, PiHH = person in household; PoHH = person outside household; HV = home visit.
