# Supplementary material for: Effectiveness of a digital clinical decision support algorithm for guiding antibiotic prescribing in pediatric outpatient care in Rwanda: A pragmatic cluster non-randomized controlled trial
Source: PLoS Med. 2026 Feb 26;23(2):e1004692. doi: 10.1371/journal.pmed.1004692 (PMC12944774; doi:10.1371/journal.pmed.1004692)

S3 Figure: Antibiotic plots for the intention-to-treat population.

Left: Prevalence of antibiotic prescription for all children and across sub-groups for four study periods. Right: Absolute differences in antibiotic prescription for two comparisons: intervention-control comparison (A: early intervention versus B: control) and before-after comparison (B: control versus B: early intervention).

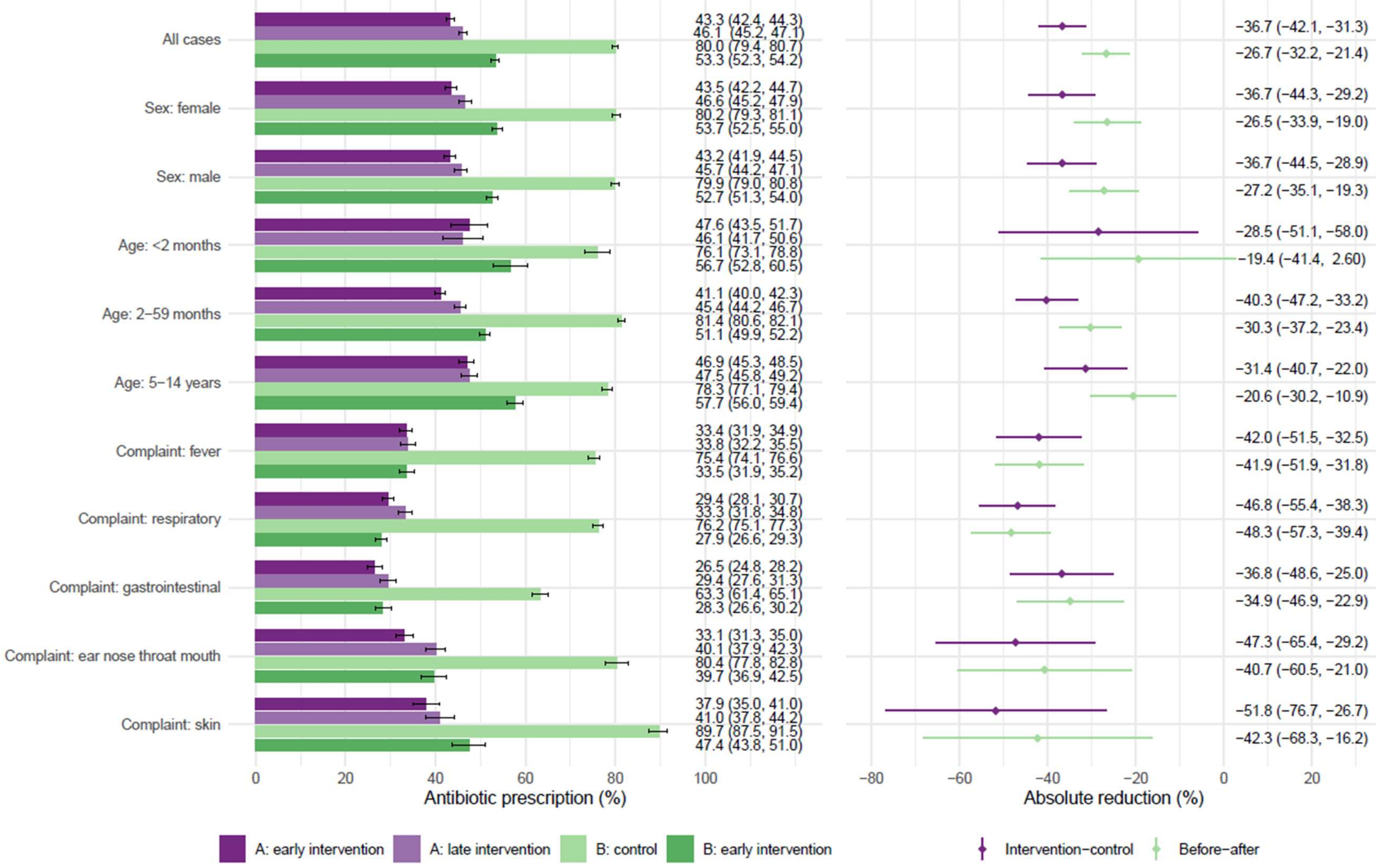

Supplement: S3 Fig — (PDF) [file pmed.1004692.s006.pdf]
