# Supplementary material for: Effectiveness of a digital clinical decision support algorithm for guiding antibiotic prescribing in pediatric outpatient care in Rwanda: A pragmatic cluster non-randomized controlled trial
Source: PLoS Med. 2026 Feb 26;23(2):e1004692. doi: 10.1371/journal.pmed.1004692 (PMC12944774; doi:10.1371/journal.pmed.1004692)

S4 Figure: Longitudinal plots of antibiotic prescription (%) for individual health facilities across implementation blocks.

Shades of purple represent health facilities originally allocated to the ePOCT+ arm (group A) and shades of green represent health facilities originally allocated to the control group (group B). The break in the graphs represents the cross-over period for group B.

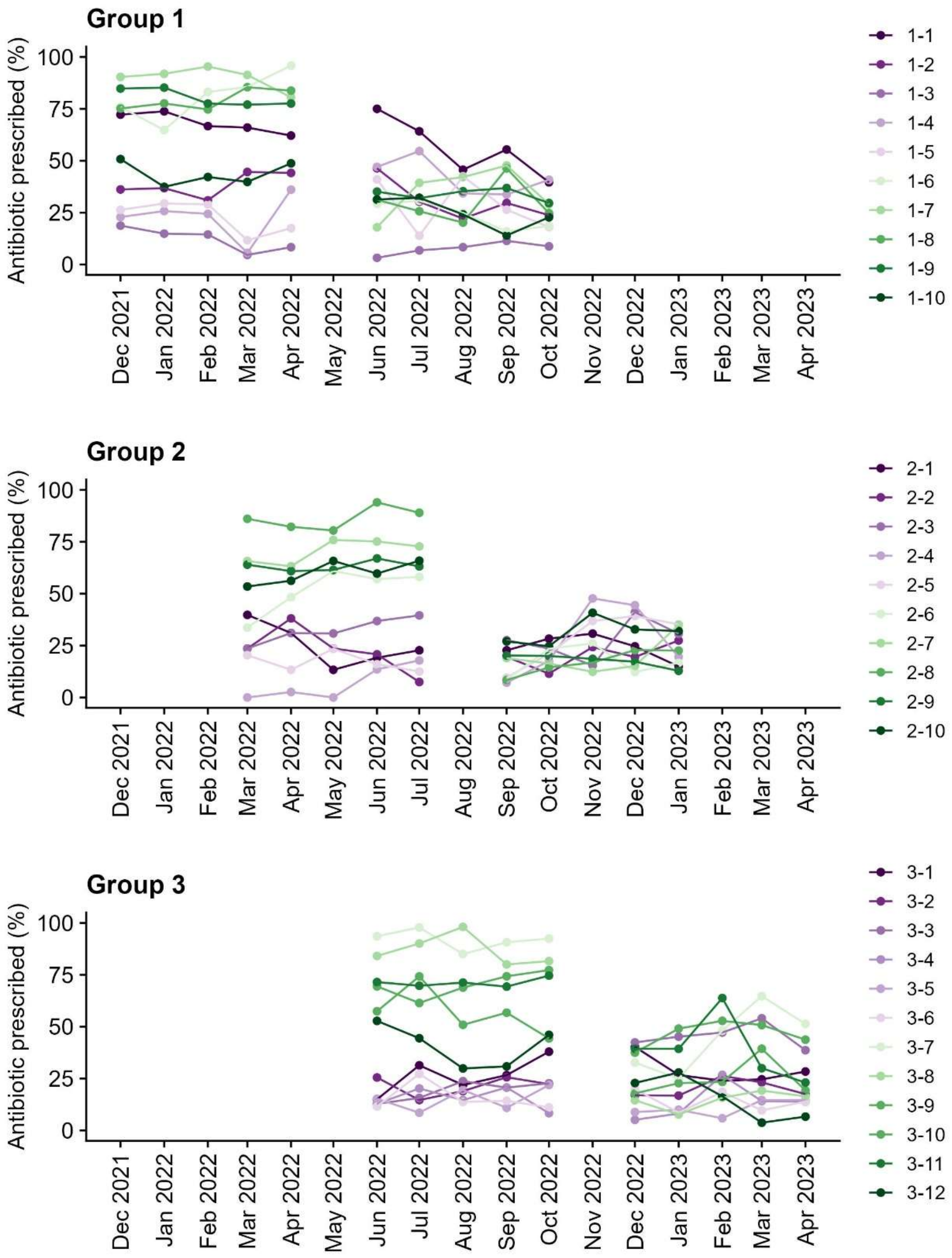

Supplement: S4 Fig — (PDF) [file pmed.1004692.s007.pdf]
