# Supplementary material for: Effectiveness of a digital clinical decision support algorithm for guiding antibiotic prescribing in pediatric outpatient care in Rwanda: A pragmatic cluster non-randomized controlled trial
Source: PLoS Med. 2026 Feb 26;23(2):e1004692. doi: 10.1371/journal.pmed.1004692 (PMC12944774; doi:10.1371/journal.pmed.1004692)

S5 Figure: Adjusted relative risks for the clinical failure outcome.

Point estimates (diamonds) with 95% confidence intervals (horizontal lines) are shown for the intention-to-treat (top) and the per-protocol (bottom) populations. Dashed vertical line represents the non-inferiority margin.

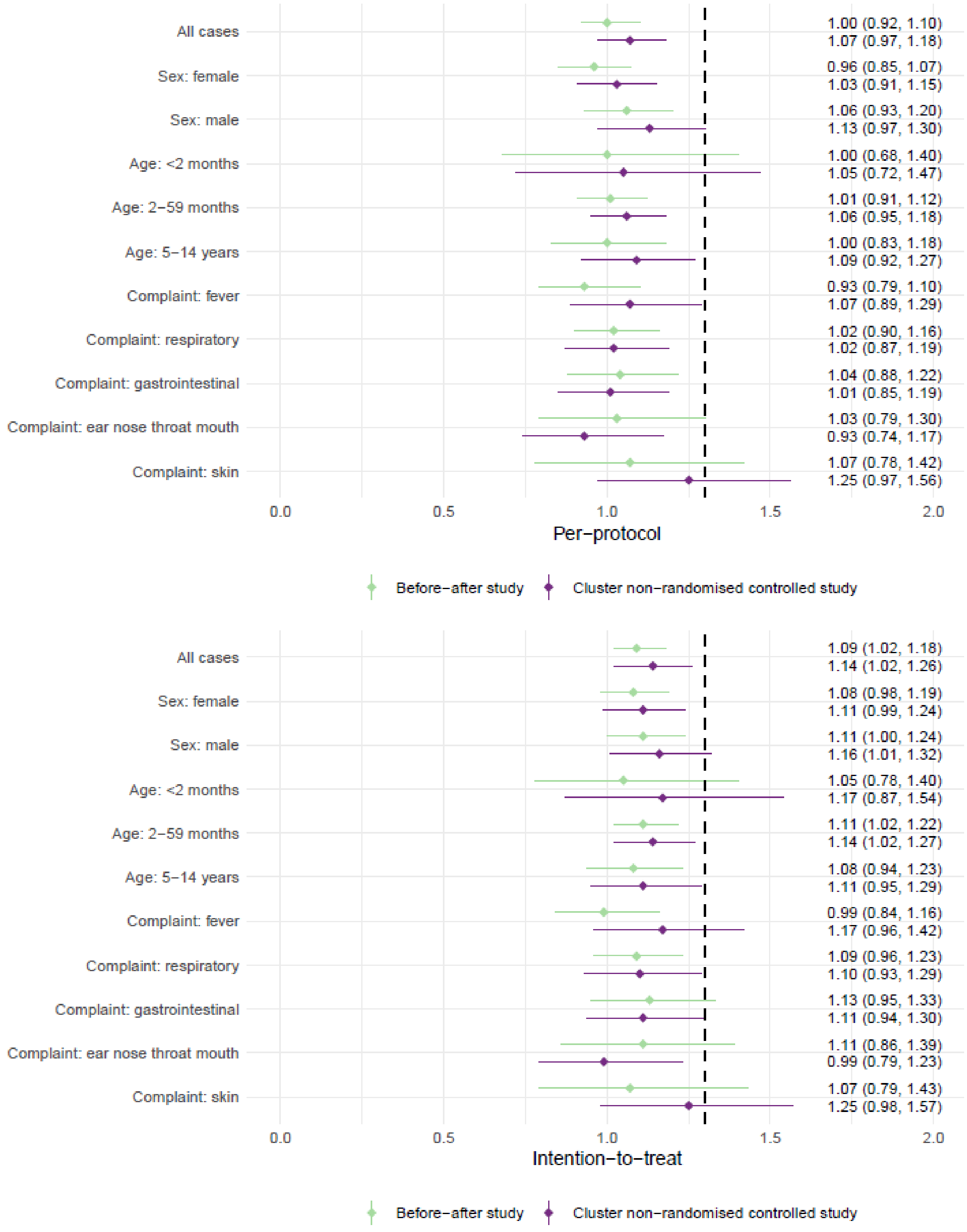

Supplement: S5 Fig — (PDF) [file pmed.1004692.s008.pdf]
