# Supplementary material for: Effectiveness of a digital clinical decision support algorithm for guiding antibiotic prescribing in pediatric outpatient care in Rwanda: A pragmatic cluster non-randomized controlled trial
Source: PLoS Med. 2026 Feb 26;23(2):e1004692. doi: 10.1371/journal.pmed.1004692 (PMC12944774; doi:10.1371/journal.pmed.1004692)
Supplement: S1 Table — (PDF) [file pmed.1004692.s009.pdf]

S1 Table: Sensitivity analysis of antibiotic prescription.

The analysis accounts for calendar time and implementation block as fixed effects in mixed effects logistic regression models.

Comparison	Analysis	Main model* aRR (95% CI)	Sensitivity analysis aRR (95% CI) †	Interpretation
Intervention-control	PP	0.28 (0.17–0.43)	0.31 (0.19–0.47)	Minimal change; estimate robust to temporal adjustment
Intervention-control	ITT	0.64 (0.49–0.78)	0.68 (0.53–0.81)	Minimal change; estimate robust to temporal adjustment
Before-after	PP	0.41 (0.39–0.43)	0.50 (0.45–0.55)	Moderate attenuation; greater susceptibility to temporal confounding
Before-after	ITT	0.83 (0.81–0.84)	0.85 (0.83–0.88)	Minimal change; estimate robust to temporal adjustment

Temporal covariate effects in sensitivity models

Covariate	Analysis	Intervention-control OR (95% CI)	Before-after OR (95% CI)
Calendar month	PP	**1.03 (1.01–1.06)**	1.02 (0.99–1.04)
Block	PP	0.58 (0.28–1.22)	0.55 (0.21–1.46)
Calendar month	ITT	0.99 (0.97–1.01)	1.00 (0.98–1.02)
Block	ITT	0.73 (0.38–1.38)	0.78 (0.30–2.01)

*Main model adjusted for age, sex, district, presenting complaint, and monthly enrollment volume (quintiles)

†Sensitivity analysis additionally adjusted for calendar month (continuous) and implementation block (categorical)

aRR: adjusted relative risk; CI: confidence interval; OR: odds ratio; PP: per-protocol; ITT: intention-to-treat
