# Supplementary material for: Effectiveness of a digital clinical decision support algorithm for guiding antibiotic prescribing in pediatric outpatient care in Rwanda: A pragmatic cluster non-randomized controlled trial
Source: PLoS Med. 2026 Feb 26;23(2):e1004692. doi: 10.1371/journal.pmed.1004692 (PMC12944774; doi:10.1371/journal.pmed.1004692)
Supplement: S2 Table — (PDF) [file pmed.1004692.s010.pdf]

S2 Table: Exploratory analysis of severe outcomes.

Severe outcomes are defined as non-referred secondary hospitalizations and deaths. Summary measures are shown across four study periods (columns).

	A_Early intervention	A_Late intervention	B_Control	B_Early intervention
Non-referred secondary hospitalizations	n = 40	n = 32	n = 24	n = 28
Severe diagnoses identified on day 0	20.0% (8 / 40)	12.5% (4 / 32)	0% (0 / 24)	7.14% (2 / 28)
Follow-up recommended on day 0	40.0% (16 / 40)	15.6% (5 / 32)	37.5% (9 / 24)	21.4% (6 / 28)
Treated with antibiotics on day 0	22.5% (9 / 40)	34.4% (11 / 32)	87.5% (21 / 24)	14.3% (4 / 28)
Cured or improved by day 7	52.5% (21 / 40)	71.9% (23 / 32)	54.2% (13 / 24)	57.1% (16 / 28)
Cured or improved of those treated with antibiotics	55.6% (5 / 9)	72.7% (8 / 11)	57.1% (12 / 21)	50.0% (2 / 4)
Cured or improved of those not treated with antibiotics	51.6% (16 / 31)	71.4% (15 / 21)	33.3% (1 / 3)	54.2% (14 / 24)
Day hospitalized (from day 0)[a]				
Day 2-3	35.0% (14 / 40)	53.1% (17 / 32)	41.7% (10 / 24)	28.5% (8 / 28)
Day 4-6	37.5% (15 / 40)	21.9% (7 / 32)	45.8% (11 / 24)	53.6% (15 / 28)
Day 7+	27.5% (11 / 40)	25.0% (8 / 32)	12.5% (3 / 24)	17.9% (5 / 28)
Length of hospitalization				
1-2 days	42.5% (17 / 40)	46.9% (15 / 32)	41.7% (10 / 24)	50.0% (14 / 28)
3-4 days	30.0% (12 / 40)	34.4% (11 / 32)	16.6% (4 / 24)	42.9% (12 / 28)
5+ days	27.5% (11 / 40)	18.7% (6 / 32)	41.7% (10 / 24)	7.1% (2 / 28)
Deaths	n = 3	n = 3	n = 1	n = 1
Severe diagnoses identified on day 0	2	3	0	1
Referred for inpatient hospitalization on day 0	2[b]	2[c]	0	1
Follow-up recommended on day 0	0	0	1	0
Hospitalized by day 7	2[b]	0	0	1
Treated with antibiotics on day 0	1	1[c]	1	1
Day hospitalized (from day 0)				
Day 0-1	2	--	--	0
Day 2-3	--	--	--	0
Day 4-6	--	--	--	1
Day 7+	--	--	--	0
Length of hospitalization				
1-2 days	--	--	--	1
3-4 days	--	--	--	0
5+ days	2	--	--	0

[a] Hospitalization on day 0-1 is defined as primary hospitalization and therefore not relevant for secondary hospitalizations

[b] Two children who were hospitalized are the same two who were referred on day 0

[c] For the child who was not referred, there is some indication that caregiver refused; this child was treated with antibiotics
