# Supplementary material for: Effectiveness of a digital clinical decision support algorithm for guiding antibiotic prescribing in pediatric outpatient care in Rwanda: A pragmatic cluster non-randomized controlled trial
Source: PLoS Med. 2026 Feb 26;23(2):e1004692. doi: 10.1371/journal.pmed.1004692 (PMC12944774; doi:10.1371/journal.pmed.1004692)
Supplement: S3 Table — (PDF) [file pmed.1004692.s011.pdf]

S3 Table: Results of unadjusted mixed effects logistic regression models.

Unadjusted outcomes comparing ePOCT+ and routine care for intervention-control and before-after analyses. These models are adjusted only for the health facility random effect but none of the other covariates.

	Intervention-control[a]		Before-after[a]	
	RR (95% CI)	p-value[b]	RR (95% CI)	p-value[b]
**Outcomes based on all enrolled cases (ITT for day 0)**				
Antibiotic prescribed at day 0	**0.54 (0.52, 0.56)**	**<0.001**	**0.67 (0.65, 0.68)**	**<0.001**
**Outcomes based on cases completed in the application by the healthcare provider (PP for day 0)**				
Antibiotic prescribed at day 0	**0.35 (0.33, 0.36)**	**<0.001**	**0.39 (0.37, 0.41)**	**<0.001**
Referral recommended at day 0	**2.35 (2.05, 2.69)**	**<0.001**	0.91 (0.76, 1.10)	0.333
Follow-up visit recommended at day 0	**0.86 (0.83, 0.89)**	**<0.001**	**0.39 (0.37, 0.41)**	**<0.001**
**Outcomes based on cases with follow-up done (regardless of application use) (ITT for day 7)**				
Clinical failure by day 7	**1.13 (1.05, 1.21)**	**0.001**	**1.28 (1.19, 1.38)**	**<0.001**
Hospitalization by day 7	**1.98 (1.57, 2.50)**	**<0.001**	**1.53 (1.18, 1.98)**	**0.001**
Re-attendace visit by day 7	1.12 (0.99, 1.26)	0.066	1.12 (0.99, 1.27)	0.076
Additional medications taken by day 7[c]	1.10 (0.98, 1.23)	0.099	**1.13 (1.01, 1.27)**	**0.041**
**Outcomes based on cases completed in the application and with follow-up done (PP for day 7)**				
Clinical failure by day 7	**1.09 (1.00, 1.19)**	**0.042**	**1.13 (1.04, 1.24)**	**0.007**
Primary hospitalization (day 0 or day 1)	**2.26 (1.56, 3.28)**	**<0.001**	**1.64 (1.08, 2.49)**	**0.021**
Secondary hospitalization (day 2+)	**1.89 (1.18, 3.02)**	**0.008**	1.65 (0.99, 2.75)	0.054
Severe outcome by day 7[d]	**1.83 (1.12, 2.98)**	**0.016**	1.62 (0.95, 2.75)	0.078
Non-scheduled re-attendance visit by day 7	**1.43 (1.19, 1.72)**	**<0.001**	**1.56 (1.29, 1.89)**	**<0.001**
Completed referral[e]	1.08 (0.64, 1.79)	0.759	1.55 (0.85, 2.69)	0.150
Completed re-attendance visit[e]	1.15 (0.90, 1.46)	0.267	**1.51 (1.12, 2.01)**	**0.007**
**Malaria outcomes based on febrile cases completed in the application**				
Tested for malaria[f]	**1.24 (1.22, 1.27)**	**<0.001**	**1.17 (1.14, 1.19)**	**<0.001**
Positive treated with antimalarials	1.09 (0.99, 1.12)	0.066	1.00 (0.80, 1.09)	0.964
Negative treated with antimalarials[g]	--	--	--	--
Untested treated with antimalarials	3.81 (0.91, 15.6)	0.066	2.20 (0.44, 10.7)	0.334
Positive treated with antibiotics	1.29 (0.62, 2.42)	0.486	0.53 (0.15, 1.69)	0.305

[a] Intervention-control analysis compares health facilities in Group A: early intervention to Group B: control; Before-after analysis compares health facilities in Group B: control to Group B: early intervention.

[b] P-values from Wald z-tests.

[c] Question about additional medicines was not asked of children who were hospitalized, hence the smaller sample size.

[d] Severe outcomes included non-referred secondary hospitalizations and deaths (3 in A: early intervention, 3 in A: late intervention, 1 in B: control, 1 in B: early intervention).

[e] Sample size reduced because only cases that were referred or for which re-attendance visit was recommended are considered.

[f] Malaria positivity rates were similar in the four groups: A: early intervention (3.2%); A: late intervention (2.8%); B: control (2.9%); B: early intervention (1.9%).

[g] Outcome was too rare to model.
